# Functionalized Wood: A Green Nanoengineering Platform for Sustainable Technologies

**DOI:** 10.1007/s40820-025-01953-4

**Published:** 2026-01-10

**Authors:** Tuo Zhang, Mingwei Gu, Yizhu Liu, Guangyao Chen, Haiyang Zhang, Liguo Chen, Xingwen Zhou, Lining Sun, Zhen Wen, Yunlei Zhou, Haibo Huang

**Affiliations:** 1 Institute of Mechanical and Electric Engineering, Jiangsu Province Key Laboratory of Embodied Intelligence Robotics Technology, Soochow University, Suzhou, Jiangsu 215123 People’s Republic of China; 2 Institute of Functional Nano and Soft Materials (FUNSOM), Jiangsu Key Laboratory for Carbon-Based Functional Materials & Devices, Soochow University, Suzhou, Jiangsu 215123 People’s Republic of China; 3 Hangzhou Institute of Technology, Xidian University, Hangzhou, 311231 People’s Republic of China

**Keywords:** Functionalized wood, Bio-based nanomaterials, Energy storage, Water purification, Energy conversion

## Abstract

Wood, once regarded primarily as a structural material, possesses rich physicochemical complexity that has long been underexplored. In the context of industrialization and carbon imbalance, it is now emerging as a renewable and multifunctional platform for green nanotechnologies. Recent advances in wood nanotechnology have enabled the transformation of natural wood into programmable substrates with tailored nanoarchitectures, establishing it as a representative class of bio-based nanomaterials. This review systematically categorizes wood-specific nanoengineering strategies—including thermal carbonization, laser-induced graphenization, targeted delignification, nanomaterial integration, and mechanical processing—highlighting their mechanisms and impacts on wood’s multiscale structural and functional properties. Importantly, these functionalization strategies can be flexibly combined in a modular, “Lego-like” manner, enabling wood to be reconfigured and optimized for diverse application scenarios. We summarize recent progress in applying functionalized wood to sustainable technologies such as energy storage (e.g., metal-ion batteries, Zn–air systems, supercapacitors), water treatment (e.g., adsorption, photothermal filtration, catalytic degradation), and energy conversion (e.g., solar evaporation, ionic thermoelectrics, hydrovoltaics, and triboelectric nanogenerators). These studies reveal how nanoengineered wood structures can enable efficient charge transport, selective adsorption, and enhanced light-to-heat conversion. Finally, the review discusses current challenges—such as scalable fabrication, material integration, and long-term environmental stability—and outlines future directions for the development of wood-based platforms in next-generation green energy and environmental systems.

## Highlights

- The intrinsic hierarchical, anisotropic, and porous architecture of wood provides a structurally programmable scaffold that supports subsequent nanoengineering strategies, enabling multiscale property modulation for diverse sustainable applications.
- Wood-specific hierarchical nanoengineering strategies—including carbonization, delignification, laser-induced graphene formation, and nanomaterial integration—are systematically categorized to enable tunable structures and properties across multiple length scales.
- Functionalized wood with nanostructures enables sustainable solutions in energy storage (e.g., Zn–air batteries, supercapacitors), water treatment (e.g., adsorption, filtration), and renewable power generation (e.g., solar-thermal, thermoelectric and hydrovoltaic systems).

## Graphical abstract

**Figure:**
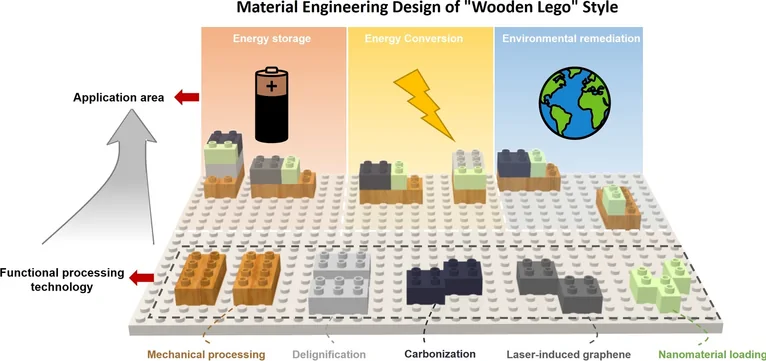

## Introduction

Throughout Earth’s evolutionary history, plants—particularly trees—have acted as fundamental agents in maintaining ecological balance and planetary homeostasis, mediating carbon fluxes, facilitating water transport, and converting solar energy into storable biological forms [1]. As the core structural component of terrestrial vegetation, wood has long contributed to ecological stability through its inherent porosity, carbon fixation capacity, and multiscale architecture. Yet, despite its ecological significance, human understanding and technological utilization of wood have remained limited for much of history. The advent of industrialization and the extensive use of fossil fuels have disrupted this natural balance, intensifying environmental degradation [2]. Wood has been redefined as a versatile platform for ecosystem and energy governance [3]. It embodies both inherent multiscale porosity and a built-in capacity for carbon storage originating from the biological growth processes of trees, and is becoming a multifunctional material that enables the integration of nature and technology [4]. Functionally, functionalized wood can simultaneously incorporate pollutant detection [5], adsorption, and photocatalytic degradation modules [6], or even be configured as a high-specific-energy storage device [7]. Functionalization of wood not only enables high performance beyond the limitations of conventional remediation methods, but also reduces associated pollution generation by promoting closed-loop life cycle management. This material revolution—also inspired by lessons drawn from natural ecosystems—has created a novel synergy between earth restoration and clean energy development, addressing ecological restoration and ensuring technological feasibility to help sustain human advancement [8].

Wood has tremendous ability for functionalization based on its hierarchical structure and chemistry [9]. As a bio-based material, its natural components, architecture, and physicochemical properties also allow wood to undergo adaptive treatments including carbonization, delignification, and incorporation of nanomaterials [10]. Wood is primarily composed of cellulose, hemicellulose, and lignin, wherein cellulose and hemicellulose provide mechanical strength by forming a polymeric network, while lignin imparts both hydrophobicity and mechanical stability. The complex multiscale porous structure—composed of vessels, tracheids, and micropores—provides a high specific surface area and excellent permeability, facilitating the formation of conductive carbon materials during carbonization [11]. In addition, the aromatic structure in lignin aids in forming high-performance carbon during pyrolysis [12]. The abundant surface hydroxyl (–OH) groups allow effective chemical reactions to promote lignin removal during delignification, resulting in enhanced transparency, flexibility, and hydrophilicity. Delignified wood retains the native fibrous and porous structure, enabling it to serve as a substrate for the incorporation of nanomaterials [13]. Its porous matrix, combined with a reactive surface, allows for stable integration with carbon nanotubes, metal nanoparticles, or metal oxides, thereby providing multifunctionality such as photocatalysis, antibacterial properties, and improved electrochemical performance. Functionalization strategies define the future applications of wood in advanced technologies [14–18].

**Fig. 1:**
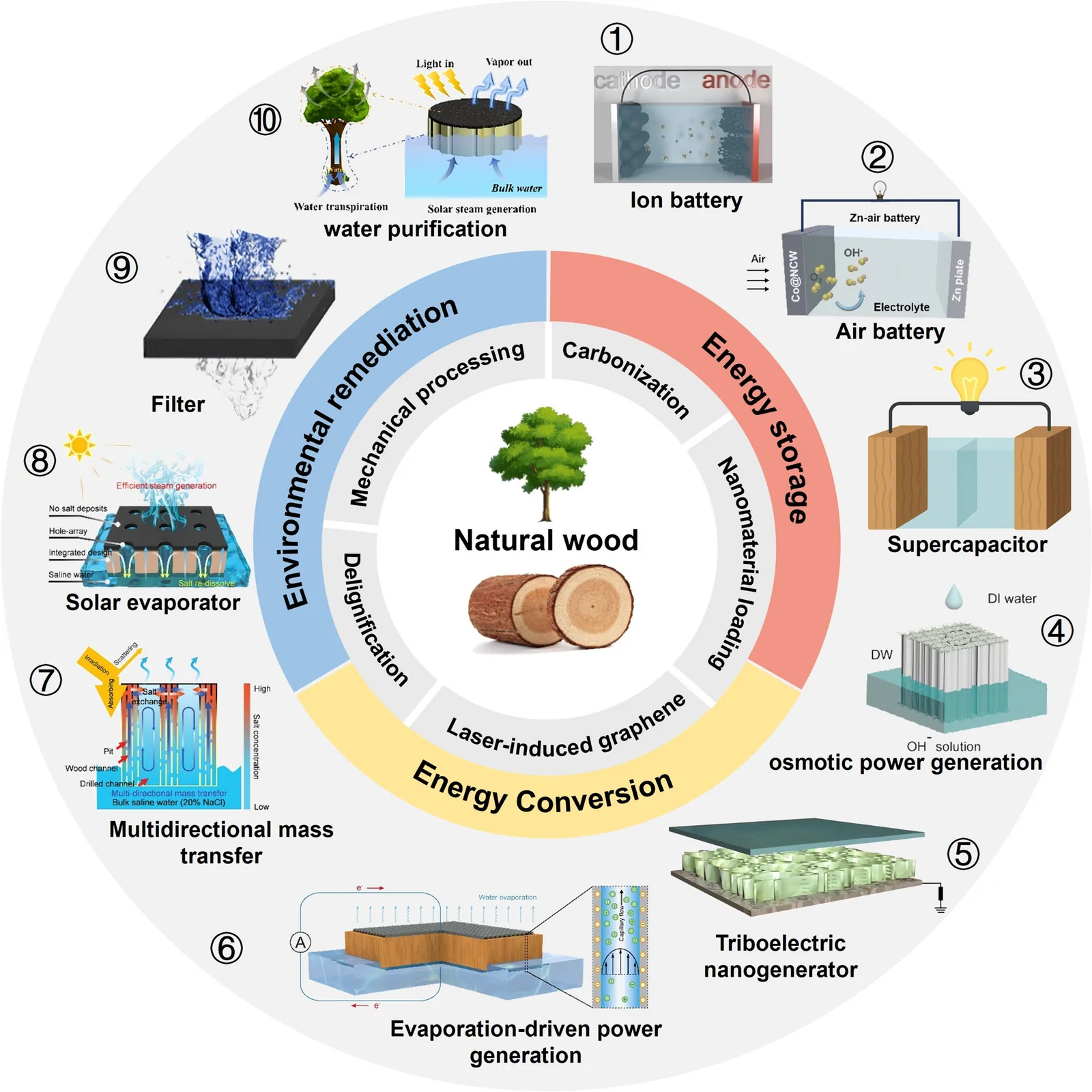
Functionalized natural wood with key processing strategies and representative applications in energy storage, energy conversion and environmental remediation Reproduced with permission from Elsevier, copyright 2024; Elsevier, copyright 2022; American Chemical Society, copyright 2024; American Chemical Society, copyright 2020; Wiley–VCH, copyright 2019; American Chemical Society, copyright 2017; Elsevier, copyright 2024

Functionalized wood has significant applications in green technology, particularly in energy storage, water treatment, and energy conversion [19–22]. Treated wood possesses a native porous structure, high specific surface area, and reactive functional groups. These characteristics enable further functionalization through processes such as carbonization, delignification, and nanomaterial integration. As a nanoengineering platform, wood's native structure is further enhanced with nanoscale features, enabling more precise control over its properties [23]. In practice, these functionalization strategies can be flexibly assembled in a modular, “Lego-like” manner, allowing researchers to reconfigure and tailor wood’s properties toward specific performance demands across diverse applications. Building upon these intrinsic structural merits and tunable functionalities, functionalized wood has emerged as a versatile platform in green technology, with particularly significant applications in energy storage and conversion as well as water purification. Its native porous architecture, high specific surface area, and abundant reactive functional groups enable functionalization approaches such as carbonization, delignification, and nanomaterial integration, which can be further enhanced by introducing nanoscale features for precise property modulation [24]. In energy storage and conversion, recent studies have demonstrated that carbonized wood frameworks [25–28], when combined with strategies such as heteroatom doping, single-atom catalysis, and hybrid nanomaterial incorporation, can serve as high-surface-area conductive electrodes for zinc–air batteries, metal-ion batteries, and supercapacitors, delivering improved electrical conductivity, catalytic activity, and cycling stability, while benefiting from the intrinsic mechanical robustness and lightweight nature of wood [29]. However, challenges remain in enhancing long-term durability under practical operating conditions, overcoming sluggish oxygen reaction kinetics, and achieving scalable, cost-effective manufacturing, with future research trends emphasizing multifunctional electrode architectures and integrated solid-state systems [30–33]. In water purification, functionalized wood has been applied in adsorption of heavy metals and dyes, photothermal desalination, membrane filtration, and catalytic degradation, with performance enhancements achieved through pore structure optimization, surface chemistry tuning, and incorporation of photocatalytic or antibacterial agents; nevertheless, issues such as maintaining high flux and selectivity under fouling conditions, enabling efficient regeneration, and integrating these materials into scalable modular systems persist, and emerging directions focus on developing hybrid photothermal–catalytic systems and adopting green fabrication strategies. Through targeted structural modification and hybridization, functionalized wood is expected to address critical challenges in both energy and water domains, supporting the development of next-generation sustainable technologies. The purpose of this review is to provide a comprehensive summary of the main functionalization approaches to wood and their applications in energy storage, water purification, solar energy utilization, and hydropower generation. The review begins with an introduction to the structure and composition of wood, examining how its porous architecture and mechanical strength create favorable conditions for functionalization. Subsequently, the review discusses several key functionalization methods, including chemical modification, physical treatment, and nanocomposite integration, and describes how these processes endow wood with new attributes. Finally, the review systematically explores the application of functionalized wood in green technologies, focusing on energy storage devices such as supercapacitors, metal-air batteries, and ion batteries; water treatment strategies including catalytic degradation, filtration, and adsorption; and solar-driven wastewater treatment and seawater desalination. In addition, the review offers a prospective view of future development directions for functionalized wood as a green technology. Given the urgent demand and accelerating pace of progress in sustainable technologies, functionalized wood is expected to demonstrate tremendous potential in supporting global sustainable development (Fig. 1).

## Structural and Chemical Constituents of Natural Wood

**Fig. 2:**
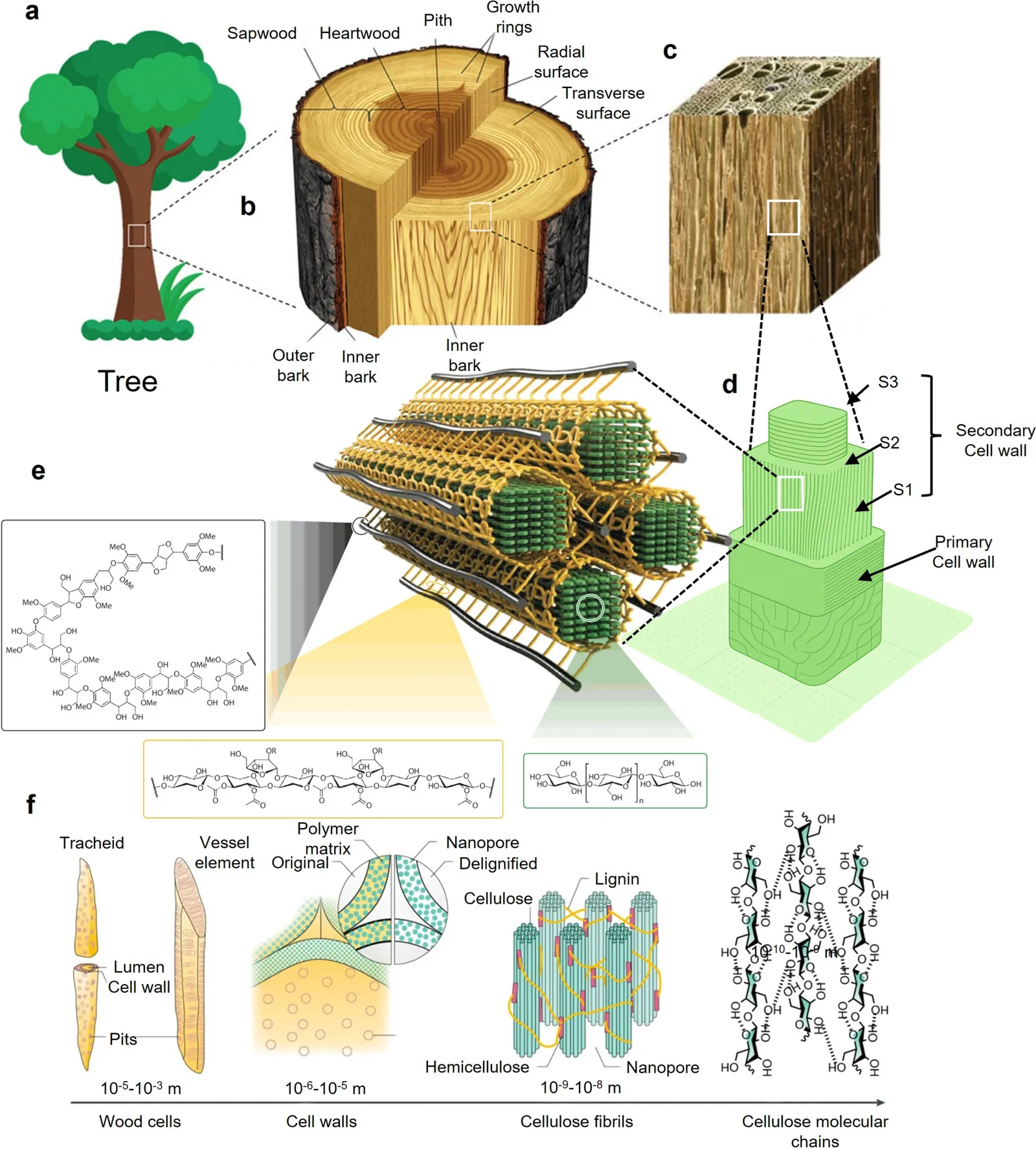
Multiscale structural analysis of wood from tree anatomy to molecular architecture. **a** Tree structure diagram illustrating the distribution and biological roles of sapwood, heartwood, pith, and growth rings. This overview highlights the functional zones within a tree trunk that contribute to mechanical support, water transport, and metabolic activity. Reproduced from Ref. [39] with permission from Wiley–VCH, copyright 2023. **b** Cross-sectional view of the trunk showing anatomical landmarks including the radial and transverse planes, outer and inner bark, and cambium layer. This view provides essential context for understanding how wood is processed and analyzed along different grain orientations. Reproduced from Ref. [39] with permission from Wiley–VCH, copyright 2023. **c** Magnified view of wood structure showing the arrangement of cell walls, reproduced from Ref. [39] with permission from Wiley–VCH, copyright 2023. **d** Schematic diagram of the ultrastructure of wood cells, depicting the hierarchical structure of the primary and secondary cell walls. **e** Chemical structures of key wood components, including cellulose and lignin, adapted from Ref. [40] with permission from The Author(s), copyright 2020, under Creative Commons CC BY license. **f** Comparative illustration of wood cells at the tracheid and vessel element scales, and the molecular arrangements of cellulose, hemicellulose, and lignin within the cell wall, reproduced from Ref. [9] with permission from Springer Nature, copyright 2020

Owing to its hierarchical structure spanning from the macro to the nanoscale, wood possesses native nanofeatures—such as aligned cellulose microfibrils and nanoscale pores—that facilitate surface interactions, molecular transport, and energy conversion. These characteristics make wood an ideal substrate for nanoengineering strategies. As shown in Fig. 2a–c, wood exhibits hierarchical porous and anisotropic structures across scales ranging from the macro-tree form (approximately 1–30 m) to the nanoscale architecture of the cell wall [2]. Its multiscale porous system comprises vessels (approximately 100 μm), tracheids (approximately 20–40 μm), pits (approximately 5 μm), and nanoscale gaps between cellulose microfibrils, supporting efficient multiphase transport and energy transfer among ions, molecules, fluids, and photons [9, 34]. Significant structural differences exist between softwoods and hardwoods: tracheids dominate in softwoods for transportation and mechanical support, whereas hardwoods utilize vessels and fibers for fluid conduction and mechanical strength. The macroscopic structures, including bark, cambium, sapwood, heartwood, and pith, collectively form a concentric network characterized by high permeability and water uptake capacity, making wood highly amenable to functional modification [11]. At the cellular level, vessels, fibers, and rays, respectively, facilitate longitudinal transport, mechanical reinforcement, and radial conduction [35]. As illustrated in Fig. 2d, e, the cell wall consists of a primary wall and a multilayered secondary wall (S1–S3), with the S2 layer—rich in cellulose microfibrils (approximately 3–5 nm) aligned at approximately 0°–30°—being primarily responsible for mechanical properties. Within these microfibrils, alternating crystalline (approximately 100–250 nm) and amorphous domains contribute synergistically to the stiffness, flexibility, and hydrophilicity of the cell wall[36]. This sophisticated hierarchical construction underpins the mechanical integrity, transport capability, and multifunctional potential of wood [37, 38].

As shown in Fig. 2d, wood is a naturally occurring composite composed of fibers and a cellulose–hemicellulose–lignin matrix, with cellulose being the principal structural component that forms hydrogen-bonded microfibrils. Within these microfibrils, there are crystalline regions that confer strength and rigidity, and amorphous regions that confer flexibility and extensibility, thus endowing wood with mechanical durability and resistance to biodegradation [41, 42]. As presented in Fig. 2f, natural cellulose exists predominantly as cellulose I, mainly in the Iβ form found in woody plants; upon regeneration or alkali treatment, it converts to cellulose II, which is more thermally stable, exhibits enhanced mechanical strength, and demonstrates improved biocompatibility [43]. The hierarchical structure, reinforced by hydrogen bonding and van der Waals forces, provides wood with outstanding durability, while the abundance of exposed hydroxyl groups enables chemical modification. Chemical modification of cellulose permits the introduction of various functional groups, such as carboxyl, amino, or sulfonic acid groups, onto the polymer backbone, thereby modulating its surface charge, hydrophilicity, and reactivity. These modifications broaden the applicability of cellulose-based materials in sustainable technologies [44, 45]. Functionalized cellulose can serve as a flexible substrate, binder, or ion-conducting membrane in energy storage devices such as batteries and supercapacitors, thereby enhancing mechanical integrity and electrolyte compatibility. In the context of water treatment, cellulose derivatives featuring large surface areas and active binding sites exhibit a high adsorption capacity for heavy metal ions, dyes, and organic contaminants, while also providing antimicrobial functionality [46–48]. Regarding solar energy and hydrovoltaic electricity generation, cellulose-based composites can act as lightweight, porous scaffolds that are thermally insulating and capable of harvesting light, evaporating water, and facilitating ion transport [49–51]. Hemicellulose, a branched polysaccharide, binds cellulose and lignin together within the plant cell wall, forming a flexible matrix stabilized by hydrogen bonds and van der Waals interactions. Hemicellulose is composed of polysaccharides such as xylan, xylose, mannose, and glucomannan, which are interspersed between cellulose microfibrils and contribute to enhancing toughness and elasticity, regulating moisture retention, and limiting wood decay [52].

Lignin is the second most abundant component in wood and forms a complex three-dimensional network with cellulose and hemicellulose, contributing to the compressive strength and structural integrity of the wood matrix. It also regulates water and nutrient transport within the wood cell wall and organizes wood density and cellular architecture through lignin–carbohydrate complexes (LCCs) [53, 54]. The dense and hydrophobic lignin matrix suppresses accessibility to chemical solutions and restricts subsequent modifications or material infusions into the wood structure. To enhance modifiability and accessibility, delignification—the effective and selective removal of lignin—has emerged as a key pretreatment strategy to enable greater tunability and functionality of the wood framework [13, 55]. Delignification displaces lignin from cellulose by dissolving the contiguous micropores formed by lignin deposition, enhances porosity, increases hydrophilicity, and exposes the cellulose templating structure [56]. These disruptive changes create a highly porous and reactive scaffolding architecture that optimizes the immobilization and insertion of materials to replicate or expand functional capabilities in composite applications. Delignified wood, originally part of wood-based biomaterials, exhibits improved ion transport properties and provides a suitable matrix for the incorporation of conductive fillers, making it applicable for constructing electrodes and separators in conventional batteries and supercapacitors [57]. Similarly, the enlarged surface area and enhanced wettability of delignified wood facilitate the adsorption of contaminants and the immobilization of photocatalysts or antimicrobial agents for water treatment applications [58]. Delignification is also advantageous for solar energy utilization and hydrovoltaic electricity generation, as it enhances light transmission, water transport, and charge transfer efficiency, while simultaneously reducing optical scattering and strengthening capillary action [59, 60]. Overall, delignification represents a transformative strategy for wood, establishing it as a versatile and sustainable platform for high-performance applications in energy conversion, environmental remediation, and advanced material systems [36].

## Functionalization Treatment Strategies of Wood

Natural wood architectures can be tailored through functionalization strategies owing to the anisotropic and porous nature of wood. As a nanoengineering platform, wood's inherent structure is enhanced with nanoscale features, enabling precise control over its properties. Carbonization transforms wood into ideal carbon frameworks that are both electrically conductive and mechanically robust [61]; laser-induced graphene (LIG) enables the localized transformation of wood surfaces into graphene-like conductive networks with strong interfacial bonding to the wood substrate [62]; delignification enhances porosity, hydrophilicity, and light transmittance by selectively removing lignin; and the integration of nanomaterials, such as metal oxides, carbon nanotubes, or single-atom catalysts, imparts specific electrochemical, catalytic, or photothermal functionalities to wood-based materials [53]. These methods frequently overlap and, when combined, can fulfill the multiple functional requirements demanded by various advanced applications. For instance, carbonized or LIG-patterned wood facilitates efficient charge transport as electrode materials for energy storage devices [63]; delignified structures enhance light absorption and water interaction for solar steam generation and water purification [64]; and nanomaterial-functionalized wood not only improves ionic transport and reaction kinetics for hydrovoltaic energy harvesting, but also enhances redox activity, selectivity, and catalytic efficiency across diverse hydrovoltaic systems [65, 66]. These functionalization strategies significantly expand the tunability of wood properties and broaden its application landscape.

### Wood Machining and Cutting Techniques

**Fig. 3:**
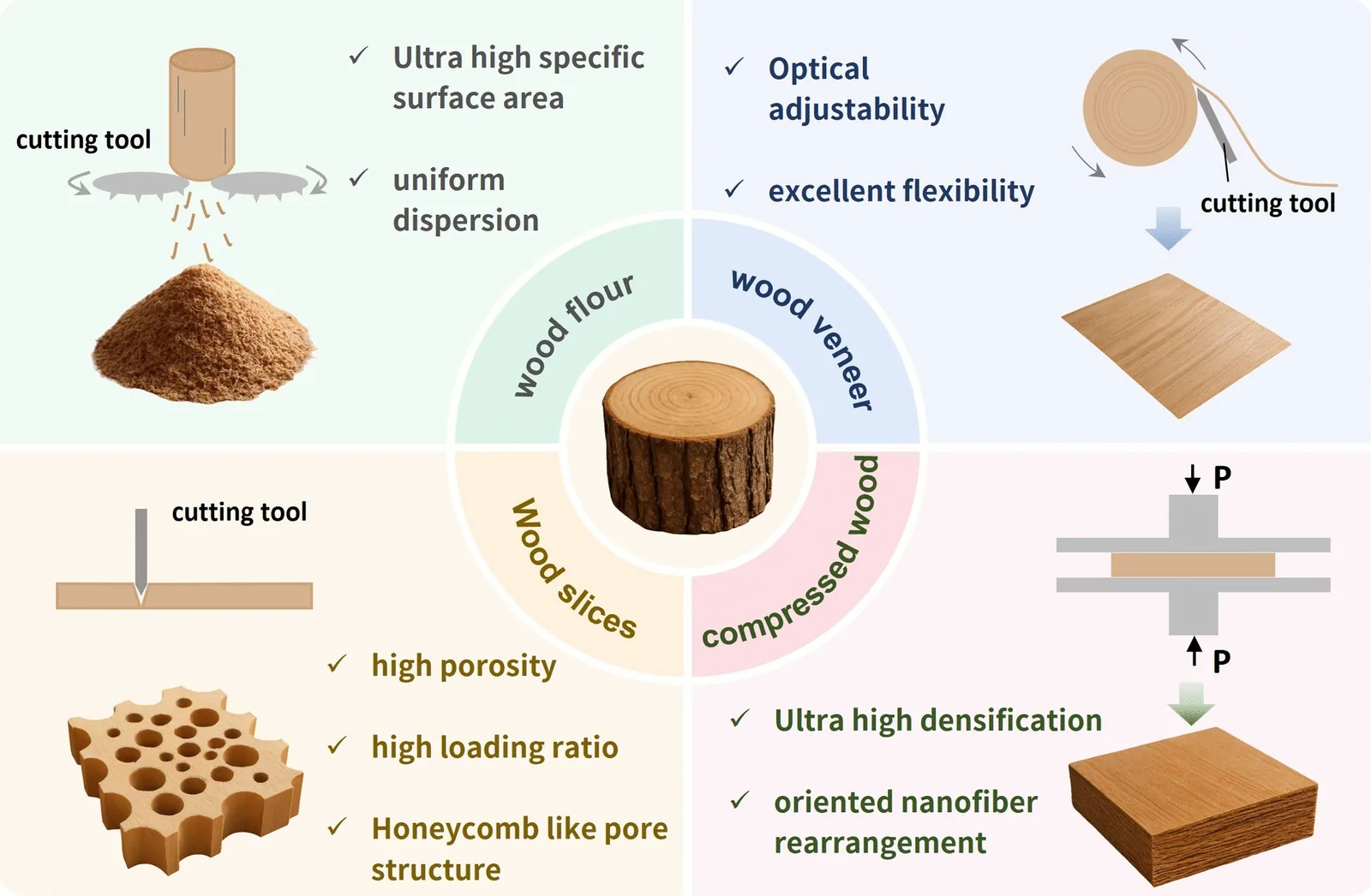
Mechanical processing of wood into four forms—wood flour, veneer, slices, and compressed wood

**Fig. 4:**
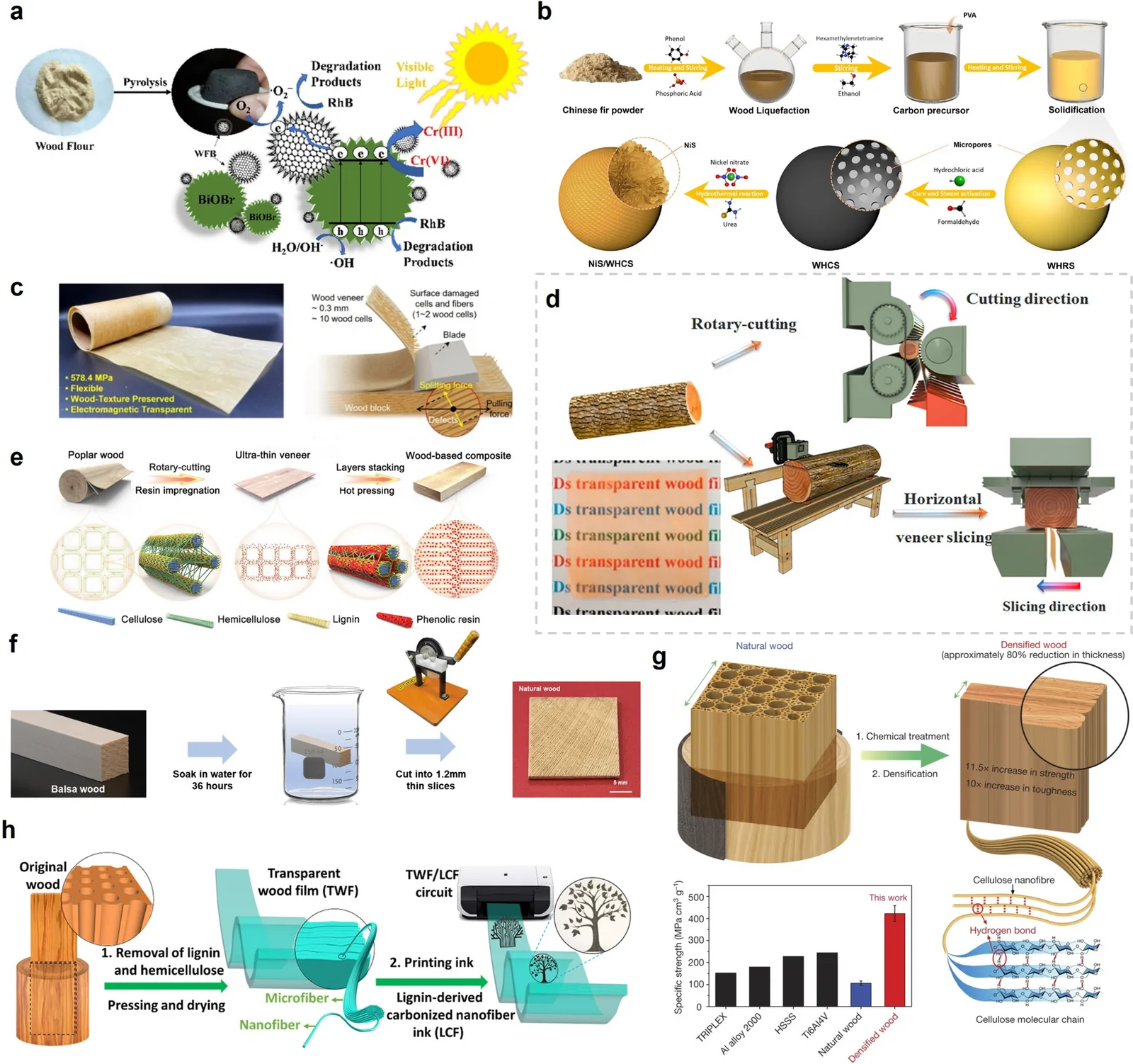
Representative examples of wood mechanical processing and applications. **a** Biochar (WFB) derived from industrial wood flour waste combined with BiOBr for visible-light-driven photocatalysis, reproduced from Ref. [67] with permission from Elsevier, copyright 2020. **b** Hollow carbon spheres (WHCS) from liquefied wood powder decorated with NiS nanosheets for high-performance supercapacitors, reproduced from Ref. [68] with permission from American Chemical Society, copyright 2023.** c** Molecular-level defect repair of ultraflexible wood veneer for sustainable structural applications, reproduced from Ref. [69] with permission from American Chemical Society, copyright 2025. **d** Transparent wood films impregnated with quantum dots for photoluminescent applications, reproduced from Ref. [70] with permission from American Chemical Society, copyright 2022. **e** Biomimetic lignification and resin infusion for fabricating ultra-hard wood-based composites, reproduced from Ref. [71] with permission from The Author(s), copyright 2025, under Creative Commons CC BY license.** f** Preparation of thin wood slices via water immersion and blade cutting for gas sensing devices, reproduced from Ref. [72] with permission from John Wiley and Sons, copyright 2025.** g** Chemical pretreatment followed by hot pressing to obtain ultra-densified structural wood, reproduced from Ref. [73] with permission from Springer Nature, copyright 2018. **h** Fabrication of all-wood-based flexible electronic circuits through compression and lignin-derived carbon nanofiber ink printing, reproduced from Ref. [55] with permission from American Chemical Society, copyright 2022

Mechanical processing is an essential key step in the process of transforming natural wood into functional device substrates or components. Its core goal is to shape the original wood into a specific form, laying the foundation for subsequent functional treatments such as chemical modification, composite, and deposition. As shown in Fig. 3, wood mechanical processing can be categorized into four types based on the target morphology. By strong grinding (ball milling, hammer milling, etc.), wood is crushed into micrometer sized powders, significantly increasing the specific surface area and suitable for high adsorption materials or composite fillers. As shown in Fig. 4a, Cui et al. [67] used industrial wood flour waste to prepare biochar (WFB) and combined it with bismuth oxybromide (BiOBr) as a carbon carrier to develop an efficient visible light driven photocatalyst (WFB/BiOBr) for environmental remediation. As shown in Fig. 4b, Li et al. [68] used liquefied wood powder to prepare hollow carbon sphere carriers (WHCS) and constructed high-performance core–shell structure supercapacitor electrodes (NiS/WHCS) by loading nickel sulfide (NiS), achieving a breakthrough application of biomass carbon materials in the field of energy storage. Using precision rotary cutting technology, the raw wood segments are rotated and cut into large-area thin wood sheets (veneer) with uniform thickness, suitable for flexible electronic substrates or transparent wood precursors. As shown in Fig. 4c, Zhu et al. [69] transformed low-strength wood veneer into a super flexible material comparable to synthetic materials through molecular level defect repair, while balancing environmental protection and electromagnetic performance, opening up a high-end application path for biomass materials. As shown in Fig. 4d, Tang et al. [70] developed an environmentally friendly quantum dot photoluminescent film (QDs wood film) using ultra-thin flexible transparent wood film (instead of traditional plastic) as the substrate for the first time, achieving the unity of high performance and sustainability. As shown in Fig. 4e, Xu et al. [71] developed ultra-hard wood-based composite materials (WBC) with extreme mechanical properties using natural veneer (thin wood flakes) through biomimetic lignification strategy and resin composite technology.

By relying on cutting techniques such as sawing, planning, and milling, sheet or block shaped units of specific sizes/shapes are prepared as macroscopic structural components or functional unit carriers. This technology can preserve the natural pores and cellulose orientation of wood to the greatest extent possible, making it the preferred processing method for functional devices such as sensors and electrodes. As shown in Fig. 4f, Gu et al. [72] used water immersion pretreatment combined with blade cutting technology to prepare thin wooden sheets with smooth surfaces, and successfully applied them to wooden gas sensors, significantly improving device performance. Moisture has a plasticizing effect on lignin, softening the cell wall and effectively reducing fiber tearing and burr formation during the cutting process. By using high pressure to cause the collapse of wood pores and plastic deformation of cell walls, the density and mechanical strength are significantly improved. As shown in Fig. 4g, Song et al. [73] reported a revolutionary wood strengthening technology that combines chemical pretreatment with hot pressing to transform natural wood into ultra-high strength structural materials. As shown in Fig. 4h, Fu et al. [55] innovatively applied compressed wood technology to the field of flexible electronics and developed all wood based flexible electronic circuits. The above four types of processing forms provide diverse material foundations for the multifunctional application of wood in energy, electronics, sensing, construction and other fields, promoting the transformation of renewable materials into high-performance devices.

### Thermal Carbonization of Wood Substrates

**Fig. 5:**
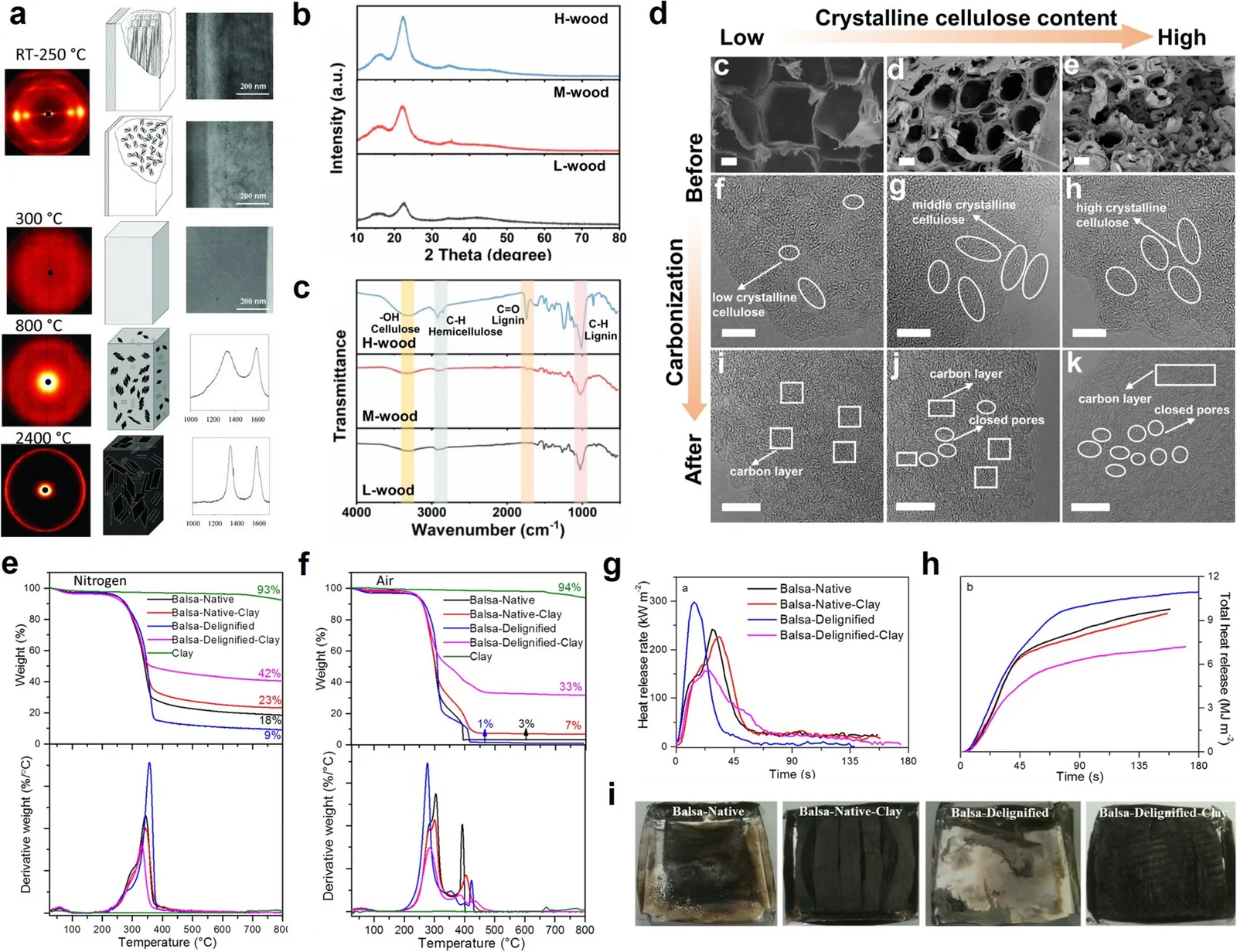
Pyrolysis of wood. **a** X-ray diffraction patterns of wood exposed to temperatures ranging from 250 to 2400 °C, demonstrating structural transformations, reproduced from Ref. [11] with permission from The Author(s), copyright 2023, under Creative Commons CC BY license. **b–d** X-ray diffraction, Fourier transform infrared (FTIR) spectroscopy, scanning electron microscopy (SEM), and high-resolution transmission electron microscopy (HRTEM) images of LIG-wood, M-wood, and H-wood, highlighting changes in cellulose crystallinity, reproduced from Ref. [74] with permission from Wiley–VCH, copyright 2021. **e–f** Thermogravimetric (TG) and derivative thermogravimetric (DTG) curves of natural wood and wood/clay nanocomposites measured under nitrogen and air atmospheres, reproduced from Ref. [75] with permission from American Chemical Society, copyright 2017. **g–i** Cone calorimetry results and corresponding photographs of residual samples from untreated and treated wood specimens, reproduced from Ref. [75] with permission from American Chemical Society, copyright 2017

The pyrolytic carbonization of wood occurs under limited-oxygen or inert atmospheres (e.g., nitrogen gas), where the organic constituents decompose at elevated temperatures to yield primarily carbonaceous or carbon-rich materials [76]. Key parameters—temperature, heating rate, and residence time—strongly affect the resulting carbon's microstructure, porosity, and electrical conductivity [11]. Advances in pyrolysis control enable the production of nanostructured, porous activated carbon with large specific surface areas. The pyrolysis process leads to distinct structural transformations, progressively evolving toward anisotropic carbon architectures [77]. Thermal degradation involves characteristic transformation stages depending on the underlying microstructure. As shown in Fig. 5a, below 200 °C, moisture evaporation dominates. Between 200 and 350 °C, mild pyrolysis decomposes major biopolymers, forming intermediate char-like structures. Between 300 and 400 °C, amorphous carbon appears without ordered morphology. Short-range ordering begins around 800 °C and continues beyond 1000 °C, yielding increasingly ordered carbon frameworks [74]. Notably, cellulose crystallinity appears to play a critical role in predicting carbon structure after pyrolysis, as reflected in Fig. 4b–d. The measured cellulose crystallinities of L-wood, M-wood, and H-wood were 49.9%, 53.2%, and 68.4%, respectively. Higher crystallinity during carbonization was correlated with more organized structures: L-wood preserved a tubular morphology resembling a honeycomb, whereas M-wood and H-wood exhibited voids with thicker walls and more compact fibrous architectures. It is therefore inferred that woods with greater crystallinity tend to form long-range graphitized carbon layers and closed-pore structures, whereas woods with lower crystallinity yield disordered carbon architectures with fewer closed pores. These findings clearly demonstrate that cellulose crystallinity significantly impacts the microstructure and pore structure of hard carbon materials derived from wood, with direct implications for performance characteristics in applications such as energy storage and adsorption. Hard carbon (i.e., carbonized wood) featuring an organized, highly graphitized microstructure exhibits high potential as an anode material due to its superior electrical conductivity, structural stability, and ion transport efficiency, particularly for energy storage devices including lithium-ion and sodium-ion batteries [78, 79]. The presence of closed pores additionally enhances performance by providing mechanical buffering during volume expansion and contraction in cycling, thereby improving cycle life. For instance, closed-pore hard carbon was fabricated by Gao et al. [80] through carbonization at 750 °C with ZnCl_2_ activation of pine wood, subsequently combined with micron-sized silicon and graphite to produce a high-performing anode. Similarly, Chen et al. [81] prepared hard carbon by hot-pressing poplar fibers at 1300 °C under nitrogen, yielding a closed-pore structure with increased interlayer spacing. This thermal-chemical process allowed cellulose reorganization and lignin cross-linking, resulting in enhanced chemical stability. Beyond structural considerations, it is critical to evaluate carbonized wood for thermal stability and combustion behavior under operational conditions. Under nitrogen atmosphere, as depicted in Fig. 5e–i, the thermogravimetric (TG) and derivative thermogravimetric (DTG) analyses reveal that the organic components—hemicellulose, cellulose, and lignin—decompose over different temperature intervals, with raw wood retaining approximately 18% residual mass after 800 °C, predominantly consisting of carbonized material [75]. Structural changes accompanying pyrolytic carbonization under inert conditions, particularly in substrate-temperature-sensitive environments, highlight the role of cellulose crystallinity in promoting graphitization and closed-pore formation, thereby enhancing conductivity, mechanical integrity, and thermal resistance.

### Laser-induced Graphene

**Fig. 6:**
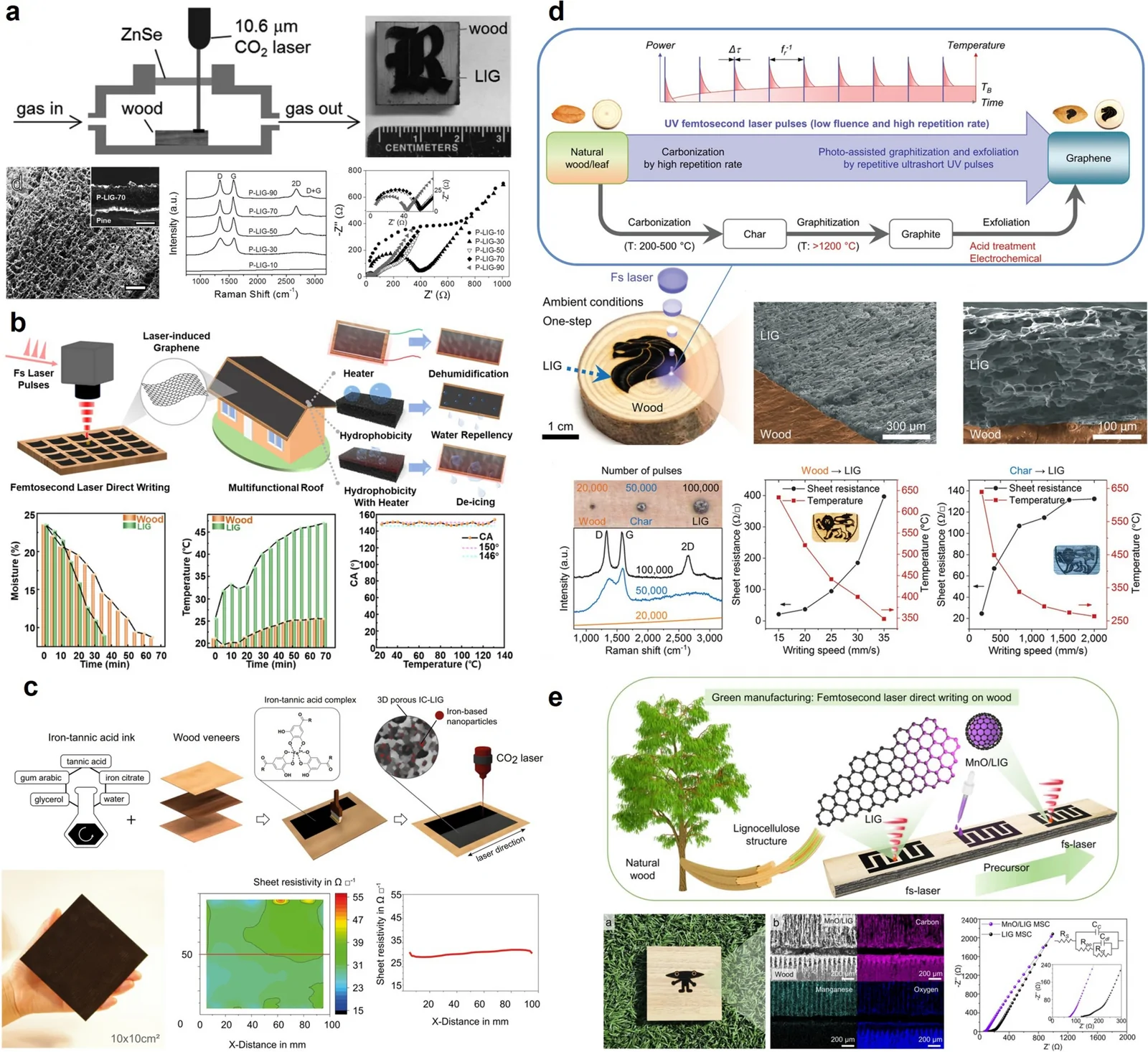
Fabrication and multifunctional applications of laser-induced graphene (LIG) on wood. **a** Fabrication of laser-induced graphene (LIG) on wood using a 10.6 μm CO_2_ laser, demonstrating a rapid and direct carbonization process. Raman spectroscopy confirms successful graphene formation, and electrochemical analysis reveals promising conductivity and capacitance characteristics for energy applications. Reproduced from Ref. [62] with permission from Wiley–VCH, copyright 2017. **b** Femtosecond laser direct writing on wood for the development of multifunctional roofing materials, evaluating moisture absorption, temperature regulation, and water repellency, reproduced from Ref. [88] with permission from Elsevier, copyright 2024. **c** LIG fabrication via CO_2_ laser writing on wood in the presence of iron ions, with analysis of writing speeds and their effects on electrical properties, reproduced from Ref. [89] with permission from The Author(s), copyright 2022, under Creative Commons CC BY license. **d** UV femtosecond laser pulses employed to produce LIG on wood, showing the effects of pulse number and laser power. Reproduced from Ref. [90] with permission from Springer Nature, copyright 2024. **e** Application of femtosecond lasers and wood-derived biomass for sustainable LIG production, highlighting its potential for environmentally friendly manufacturing practices, reproduced from Ref. [93] with permission from Wiley–VCH, copyright 2019

LIG was initially studied through laser-induced reduction of graphite oxide on polyimide (PI) substrates, and due to its rapid, scalable fabrication of transparent conductive films and other conductive products, LIG has subsequently been widely explored in lignocellulosic materials such as wood [62]. Under laser irradiation, lignin can decompose and rearrange into a three-dimensional conductive porous graphene framework. Laser-induced graphene (LIG) is produced through a pyrolytic process in which an ultrafast laser beam locally heats the surface of a precursor material at high speed, inducing carbonization and forming micron-sized graphene structures [82–84]. LIG offers precise control over the carbonization process, in contrast to conventional methods such as chemical vapor deposition (CVD) and hydrothermal synthesis, enabling the production of highly conductive graphene materials with large surface areas. The conversion from wood to carbon via photothermal processes occurs in ambient conditions at room temperature, providing a low-cost, simple, and sustainable alternative to traditional three-dimensional graphene fabrication methods [85]. Lin et al. [86] first demonstrated the production of LIG from PI substrates using a CO₂ laser, where *sp*^3^-hybridized carbon was converted to *sp*^2^-hybridized carbon via a photothermal mechanism. As indicated in Fig. 6a, Ye et al. [62] achieved direct LIG formation on wood surfaces, with increased laser power promoting C=C bond formation and enhancing conductivity. Chyan et al. [87] demonstrated that increasing the number of pulsed laser scans could incrementally improve LIG quality across various substrates. Lengger et al. [12] found that wood species with high soluble lignin content and uniform porosity were more readily converted into LIG, whereas those with variable density and distinct growth rings showed reduced conversion efficiency. As observed in Fig. 6b, Wang et al. [88] fabricated LIG electrodes from natural wood in a single-step process, suitable for hydrophobic and highly conductive applications such as green smart roofing devices. As observed in Fig. 6c, Dreimol et al. [89] developed iron–tannin coatings to create a one-step graphitizable wood precursor. Surface pretreatments, such as boric acid soaking or metal salt impregnation, further improved electrical conductivity, facilitating applications in energy storage and sensing. Femtosecond laser technology provides significant advantages for LIG fabrication on wood, as the short pulse width enables precise energy delivery with minimal substrate damage, resulting in high fabrication efficiency, reduced heat-affected zones, and compatibility with diverse wood and biomass materials. As evidenced in Fig. 6d, Le et al. [90] in 2019 achieved direct patterning of LIG onto wood and leaves using ultraviolet(UV) femtosecond lasers, producing relatively low surface resistance (~ 10 Ω sq^−1^) and fine structural resolution (line width ~ 40 µm). Miyakoshi et al. [91] fabricated an environmentally friendly supercapacitor by patterning LIG directly onto bamboo surfaces using femtosecond lasers, subsequently covering the surfaces with a NaCl-containing agarose gel to form conductive structures with high surface area and excellent rate capabilities. Nam et al. [92] employed femtosecond lasers to fabricate LIG electrodes on medium-density fiberboard (MDF), achieving a reported conductivity of 2.781 Ω sq^−1^. As observed in Fig. 6e, Kim synthesized LIG on wood and subsequently fabricated MnO/LIG heteronanostructures by drop-casting a manganese precursor followed by a second laser treatment.

Laser-induced graphene facilitates the expeditious fabrication of conductive carbon structures, which exhibit superior electrical conductivity, energy storage, and sensing properties. Moreover, the inherent porosity of wood-derived LIG enables ion transport in batteries. Consequently, LIG has the potential to enhance adsorption and catalytic processes in water treatment and to support solar energy harvesting in the future. The combination of sustainability, scalability, and versatility inherent in LIG on wood suggests the potential for a highly promising platform for next-generation green technologies [94].

### Delignification Approaches for Wood Microstructure Engineering

#### Traditional Chemical Delignification

**Fig. 7:**
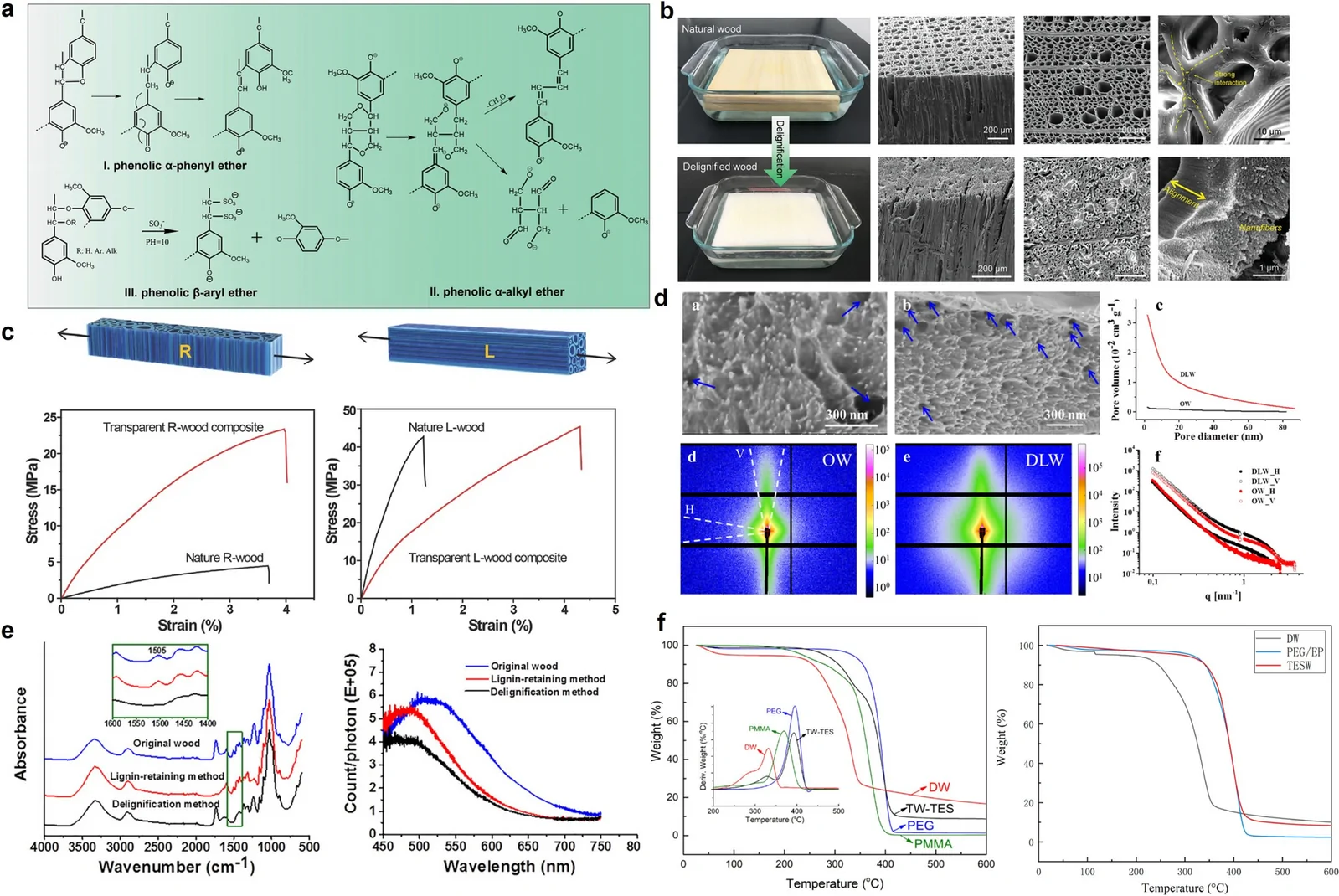
Structural and property characterization of wood before and after delignification. **a** Delignification mechanism involving different phenolic derivatives, reproduced from Ref. [13] with permission from Elsevier, copyright 2021. **b** Comparison of the appearance and SEM images of natural and delignified wood, emphasizing changes in pore structure, reproduced from Ref. [13] with permission from Elsevier, copyright 2021. **c** Stress–strain curves comparing the mechanical properties of natural wood and transparent composite wood, reproduced from Ref. [95] with permission from Wiley–VCH, copyright 2020. **d** Comparison of nanoporous structures, illustrating pore size distribution and volume differences, reproduced from Ref. [96] with permission from Wiley–VCH, copyright 2019. **e** FTIR spectra demonstrating the impact of various treatments on the functional groups of wood, reproduced from Ref. [97] with permission from Wiley–VCH, copyright 2020. **f** TGA curves comparing the thermal stability of natural and treated wood, reproduced from Ref. [56, 98] with permission from Wiley–VCH, copyright 2016, and from Elsevier, copyright 2021

Delignified wood serves as a versatile nanoengineering platform due to the emergence of nanoscale structural features and the tunable properties induced by controlled lignin removal. Partial or complete delignification represents a wood-specific nanostructuring strategy that leverages the intrinsic alignment of cellulose microfibrils and generates nanopores throughout the hierarchical matrix. Partial removal of lignin opens up cellulose fibers, forms nanopores, and improves the porosity, surface area, and optical properties of wood, while also maintaining its internal stratification and anisotropic structure [53]. This process is generally achieved through alkaline treatment, typically applied to wood chips, involving NaOH in combination with Na_2_SO_3_. The alkaline treatment disrupts the ether bonds in lignin, forming soluble lignin sulfonates and enabling selective removal of lignin while retaining the structural dimensionality, provided that the lignin content remains above 10 wt%. As shown in Fig. 7a–c, partial lignin removal separates cellulose from the middle lamella—the region richest in lignin—exposes the cellulose microfibrils, and improves light scattering, thereby enabling the development of high-performance materials such as “super wood,” which exhibits superior strength compared to steel, and elastic wood, which possesses large deformation capabilities suitable for soft robotics and flexible composites [95]. In contrast, complete removal of lignin is accomplished using chemical bleaching agents such as NaClO_2_ or H_2_O_2_. Following chemical bleaching, the residual lignin content is reduced to below 2 wt%, and the microstructural characteristics of the wood are significantly altered, with full exposure of cellulose microfibrils and the formation of nanoporosity. The reduced lignin content imparts transparency, enhances chemical reactivity, and significantly broadens the potential applications in areas such as transparent wood, energy storage, and green electronics. Nevertheless, severe oxidation during bleaching can compromise the mechanical strength of wood and raise environmental concerns, highlighting the need for more environmentally friendly delignification methods. Delignification-induced microstructural variation can be observed in Fig. 7d. SEM and SAXS analyses indicated the formation of nanoporous structures, with most pore diameters being less than 50 nm, confirming the generation of nanopores. SEM imaging revealed a more open porous architecture, while two-dimensional SAXS analysis showed markedly increased scattering in both horizontal and vertical directions, reflecting the expanded nanoporous network [96]. These findings demonstrate that enhanced porosity in wood improves surface area and opens up new opportunities for functional applications. As shown in Fig. 7e, chemical and optical property changes also accompany delignification. FTIR analysis confirmed lignin removal through the decreased intensity of the absorption peak at 1505 cm⁻^1^ [97]. As illustrated in Fig. 7f, TGA results demonstrated that treated woods (TW-TES, PEG, PMMA) exhibited considerably improved thermal stability compared to delignified wood (DW), with decomposition temperatures exceeding 400 °C (PEG) and 300 °C (TES) [56, 98]. Therefore, such treatments enhance high-temperature performance by modifying the chemical composition and broadening potential application areas. The resulting structural and functional changes induced by different delignification methods are further summarized in Table 1, highlighting their impact on the physicochemical properties and potential applications of wood.

**Table 1:** Effects of delignification methods on the physicochemical properties of wood and its functional applications

Application	Wood Species	Delignification Method	Lignin Residue Content	Process Greenness	References
Energy storage and power generation	Ochroma lagopus Swartz	2.5 wt% NaClO_2_ + acetic acid buffer (pH 4.6), 80 °C, 6 h	mostly lignin removed	Moderate treatment	[99]
	Balsa wood/ Basswood/ Poplar wood/ Pine wood	2 wt% NaClO_2_ + acetate buffer (pH 4.6), 80 °C, 6 h	mostly lignin removed	Moderate treatment	[100]
	Birch	1 wt% NaClO_2_ solution (pH adjusted to 4.6 with CH₃COOH), reacted at 80 ℃ until wood chips turned white	mostly lignin removed	Moderate treatment	[101]
	Basswood	2 wt% NaClO_2_ solution (prepared from 80% NaClO_2_ powder, pH adjusted to 4.6 with glacial acetic acid), 95 °C water bath for 9 h → rinsed 3 × with 1:1 ethanol/deionized water → freeze-dried	mostly lignin removed	Moderate treatment	[102]
	Natural wood	5 wt% NaClO_2_ (pH 4.6, adjusted with CH₃COOH), boiled for 2 h	mostly lignin removed	High Risk	[103]
	Balsa wood	2 wt% NaClO_2_ + CH_3_COOH (pH 4–5), 80 °C, 24–48 h	mostly lignin removed	High Risk	[104]
	Balsa wood	1 wt% NaClO_2_ (pH 4.6, 80 °C, 24 h) → liquid nitrogen freezing + freeze-drying; 6 wt% NaOH (RT, 8 h) for cellulosic wood	mostly lignin removed	Moderate treatment	[105]
Water treatment	Balsa wood	1 wt% NaClO_2_ + acetate buffer (pH 4.6), 80 °C, until completely white	Reduced from 24.9% to 2.9%	Moderate treatment	[106]
	Balsa wood	5 wt% NaClO_2_ solution (pH adjusted to 4 with CH₃COOH) at 80 ℃ for 8 h, repeated 3 cycles	mostly lignin removed	Moderate treatment	[107]
	Wood strips	8 wt% NaOH solution at 60 ℃	mostly lignin removed	High Risk	[108]
	Balsa wood	2.5 M NaOH + 0.4 M Na_2_SO_3_, 80 ℃ 4 h → 2.5 M H2O2, 80 ℃ 4 h	mostly lignin removed	High Risk	[109]
	Balsa wood	2.5 M NaOH + 0.4 M Na_2_SO_3_, 80 °C, 3 h; H2O2, 80 °C, 3 h	mostly lignin removed	Moderate treatment	[110]
	Balsa wood	1 wt% NaClO_2_ + CH₃COOH (pH 4.6), 80 °C, refreshed every 6 h, hot water wash, freeze-dry	mostly lignin removed	Moderate treatment	[111]
	Balsa wood	1.5 wt% NaClO_2_ + NaAc buffer, pH 4.6, 80 °C, 8 h → Wash → 8 wt% NaOH, 80 °C, 8 h → Wash → Freeze at − 25 °C > 12 h → Freeze-dry 36 h	mostly lignin removed	Moderate treatment	[112]

Classification criteria of Process Greenness. Moderate treatment: Conducted with moderate reagent concentrations (e.g., 1–5 wt% NaClO_2_ or NaOH), medium temperature (60–90 °C), and reaction time of 6–12 h; waste liquids require neutralization or dilution before disposal. High Risk: Involves high reagent concentrations (> 5 wt% oxidants or strong acids/alkalis), high temperature (> 90 °C), extended reaction time (> 12 h), or the use of toxic reagents (e.g., organochlorines); waste disposal requires specialized treatment to avoid environmental hazards

#### Green Solvent-Based Delignification

Deep eutectic solvents (DES) offer distinct advantages over conventional chemical delignification methods, such as strong acid/base treatments or ionic liquid systems [113]. They are more environmentally benign, composed of less toxic, typically biodegradable and renewable ingredients. Furthermore, DES presents a sustainable option for biomass valorization owing to their high biodegradability and renewability. DES are classified as green solvents, formed by combining a hydrogen bond donor (HBD) and a hydrogen bond acceptor (HBA) in specific molar ratios, resulting in extensive hydrogen bonding networks that create a relatively stable liquid phase [114]. Choline chloride and betaine are typical HBAs, while lactic acid, oxalic acid, and glycerol serve as common HBDs, all successfully applied in green pretreatment of lignocellulosic biomass [115]. In terms of physicochemical properties, DES typically exhibit low melting points (< 50 °C), moderate viscosity, tunable viscosity, and low electrical conductivity, each adjustable through composition or by adding co-solvents [116]. Their mass transfer behavior and solvation properties depend strongly on hydrogen bonding strength and functional group composition, both of which can be readily modified by system conditions. The synthesis of DES is convenient and considerably simpler than that of traditional or ionic liquids [117]. Common preparation techniques include thermal stirring, vacuum evaporation, grinding, freeze-drying, twin-screw extrusion, ultrasonic treatment, and microwave-assisted synthesis [118]. While thermal stirring is the most widely used method, ultrasonic and microwave-assisted techniques enable faster, greener synthesis, offering promising scalability for biomass pretreatment applications [119].

**Fig. 8:**
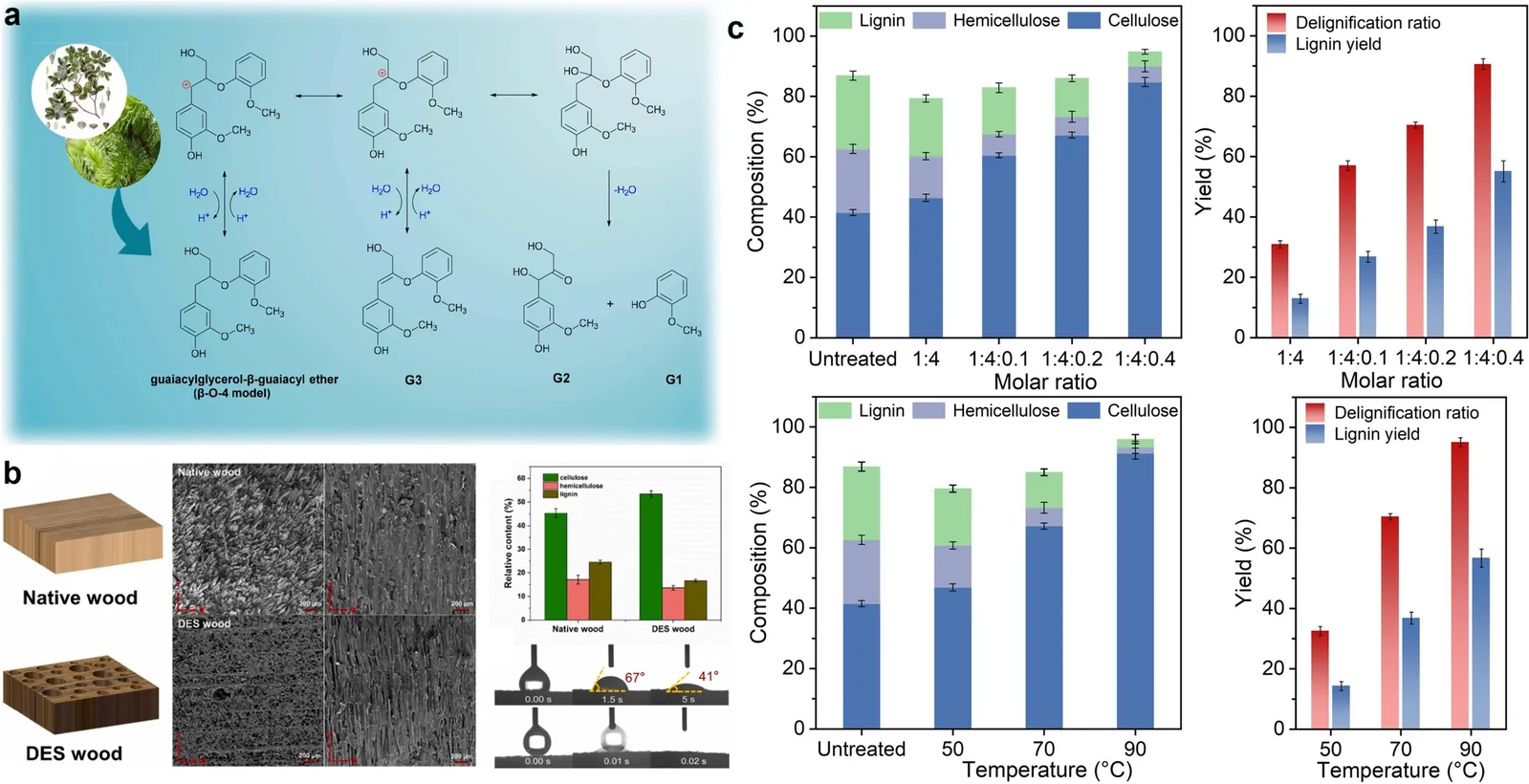
Effects of deep eutectic solvent (DES) delignification on wood structure, composition, and performance. **a** Schematic of the proposed delignification mechanism in DES, involving cleavage of β-O-4 ether linkages in lignin and sequential formation of guaiacyl-type phenolic monomers (G1–G3), reproduced from Ref. [113] with permission from Elsevier, copyright 2025. **b** Comparison of native and DES-treated wood through 3D models, SEM images, and mechanical testing, highlighting changes in microstructure and mechanical properties, reproduced from Ref. [120] with permission from Elsevier, copyright 2024. **c** Analysis of the effects of different DES molar ratios and treatment temperatures on wood composition and delignification efficiency, showing variations in lignin, hemicellulose, and cellulose contents, delignification ratio, and lignin yield, reproduced from Ref. [121] with permission from Elsevier, copyright 2023

The delignification mechanisms of deep eutectic solvents (DES) for lignocellulosic biomass are illustrated in Fig. 8a [113]. As indicated, DES function through cleavage of key chemical linkages and exhibit strong solubility for lignin. DES are capable of cleaving ester and ether bonds between lignin and hemicellulose, with the predominant cleavage of β-O-4 linkages, leading to the production of phenolic hydroxyl groups and depolymerization of lignin into lower-molecular-weight fragments [122]. Additionally, organic acid-based DES can simulate acid-catalyzed mechanisms under mildly acidic conditions to further enhance bond cleavage and depolymerization [123]. Chloride ions present in DES can also disrupt hydrogen bonding networks in lignin–carbohydrate complexes (LCCs), promoting the removal of lignin and hemicellulose [124]. Lignin displays significantly higher solubility in DES compared to cellulose, attributed to its less compact hydrogen-bonding network relative to the dense intra- and intermolecular hydrogen bonding of cellulose [125]. The Kamlet–Taft parameters (α, β, and π*) are commonly employed to evaluate DES polarity and hydrogen-bonding capabilities, where higher β and π* values indicate greater affinity for lignin [126]. In addition, molecular dynamics (MD) simulations suggest that DES with low density, low cohesive energy, high molar volume, and optimal hydrogen-bonding properties are most effective in disrupting lignocellulosic structures, thereby facilitating lignin extraction and separation. As provided in Fig. 8b, Shen et al. [120] used ChCl-LA DESs to extract lignin while preserving the cellulose framework, enhancing water transport and thermal insulation. A photothermal polypyrrole (PPy) coating prepared in situ further enabled the construction of a solar-driven wood evaporator achieving an average evaporation rate of 1.94 kg m⁻^2^ h⁻^1^ under one-sun irradiation with 83.4% photothermal efficiency. As illustrated in Fig. 8c, Wang et al. [121] proposed a novel deep eutectic solvent system (PEA-DESs), composed of pyridine hydrochloride, ethylene glycol, and AlCl_3_, enabling mild pretreatment of lignocellulose at a low temperature of 70 °C. This method effectively achieved delignification while preserving lignin structure, yielding lignin with a high β-O-4 linkage content (42.1 per 100 aromatic units) and abundant hydroxyl groups (4.97 mmol g⁻^1^). The cellulose-rich residues exhibited a maximum glucose yield of 88.2% through enzymatic hydrolysis. Moreover, modulation of the hydrogen bond acidity (α value) of PEA-DES permitted precise control over lignin structural evolution. This dual-pathway approach offers an efficient strategy for the integrated valorization of both lignin and carbohydrates, advancing the sustainable utilization of lignocellulosic biomass (Table 2).

**Table 2:** Wood-derived electrocatalysts and their performance in zinc–air batteries

References	Catalyst Type	Performance	Main Contribution
[139]	FeN_3_ single-atom catalyst	P_max_ = 152 mW cm^−2^ (liquid); 128.3 mW cm^−2^ (solid); Cycle life: 225 h / 82 h	Cascade protection of Fe–N_3_ active sites on defect-rich wood aerogel for flexible ZABs
[167]	N,S co-doped wood-derived carbon	E_onset_ = 0.93 V; E_1/2_ = 0.832 V; P_max_ = 149 mW cm^−2^; Cycle life: 125 h	Single-step N,S doping of wood carbon for high-performance metal-free ORR electrocatalysis
[142]	CoM (M = Ni, Fe, Mn, Cu) bimetallic nanoparticles	P_max_ = 168.9 mW cm^−2^; Cycle life: 400 h	Asymmetric bimetallic NPs anchored in CNT-grafted wood carbon for enhanced ORR kinetics
[171]	Co@D-NCNT (Co and N-doped carbon nanotubes)	ORR/OER gap: 0.67 V; P_max_: 245.3 mW cm^−2^ (liquid); Cycle life: 500 h	Defect-engineered chainmail electrode for seawater Zn–air battery
[99]	FeP nanoparticles + N,P co-doped carbon	E_onset_ = 0.95 V; E_1/2_ = 0.84 V; Current density: 5.20 mA cm^−2^; Capacity: 775.5 mAh g^−1^	Efficient ORR catalyst via FeP in porous N,P-doped wood carbon aerogel
[152]	Fe_x_/FeN_3_S_1_–C (Fe cluster-enhanced single atom catalyst)	E_1/2_ = 0.90 V; P_max_ = 249 mW cm^−2^; Cycle life: 425 h @ 20 mA cm^−2^; − 60 to 50 °C	Fully wood-based wide-temperature ZAB with sustainable SAC and organohydrogel electrolyte
[170]	Fe_3_O_4_ nanoparticles + single Fe–N_4_ atom sites	E_1/2_ = 0.85 V; P_max_ = 102 mW cm^−2^; Cycle life: > 100 h	Synergistic Fe_3_O_4_ and Fe SAs on wood-derived nanosheets with LS hydrogel for flexible ZABs
[172]	Fe_5_Ni_4_S_8_ nanosheets + confined carbon framework	Overpotential: 0.32 V@10 mA cm^−2^; Excellent OER performance	Confinement in carbonized wood boosts intrinsic activity of ternary sulfide catalyst
[173]	NiFe-layered hydroxide grown on biochar		In situ growth of nickel–iron layered double hydroxide (NiFe LDH) improves bifunctional activity of wood-derived carbon for Zn–air batteries
[174]	Wood-derived carbon catalyst with water activation sites		Wood template provides active water molecule activation capability for ORR enhancement
[168]	Graphitic carbon-encapsulated Co NPs on N-doped carbonized wood	0.89 V half-wave potential; 410 mV overpotential; 47.5 mW cm^−2^; 240 h stability	Self-supported chainmail electrode with excellent ORR/OER and long-term operation
[175]	Fe_3_C nanoparticles + N-doped paulownia wood carbon	0.87 V half-wave potential; 804.4 mAh gZn^−1^; 780 cycles; 78 mW cm⁻^2^	Double active-site catalyst strategy for simultaneous O_2_ and H_2_O activation
[164]	Fe–N single-atom catalyst on wood-derived carbon	0.85 V half-wave potential; 70.2 mW cm^−2^; long-term stability	Lewis acid pretreatment enables large-scale fabrication of atomically dispersed Fe–N–C SACs on porous wood carbon
[163]	Co/CoO nanoparticles on N-doped wood carbon	800 mAh gZn^−1^; 270 h cycling; voltage gap 0.84 V	3D integral electrode design with abundant triple-phase boundaries enhances ORR/OER

With adequate engineering strategies, deep eutectic solvents (DES) offer a green, efficient, and sustainable alternative to traditional acid/base or ionic liquid-based methods. DES enable targeted cleavage of critical linkages (e.g., β-O-4) in lignin while preserving the cellulose structure, facilitating effective lignin depolymerization and separation. Additionally, DES exhibit low toxicity, high biocompatibility, and are simple to synthesize, positioning them as prime candidates for the pretreatment of lignocellulosic biomass [121, 127]. Future developments will focus on designing DES formulations that selectively and efficiently extract lignin, as well as engineering multifunctional DES systems that simultaneously act as catalysts or inhibitors during hydrothermal processing. Moreover, attention will be directed towards the advancement of integrated biorefinery platforms and energy-efficient, scalable transformation technologies capable of converting both lignin and carbohydrate fractions into high-value heterogeneous products [128].

### Wood–Nanomaterial Hybrid Systems

With the growing demand for functionalization, wood–nanomaterial hybrid systems have evolved into a promising nanostructured platform and have been developed along two primary modification strategies: top-down and bottom-up [129, 130]. The top-down approach leverages the inherent hierarchical structure of natural wood, which inherently contains aligned cellulose microfibrils and nanoscale porosity, imparting new functionalities through structural reconstruction, surface modification, or nanomaterial integration—a wood-specific nanoengineering strategy that emphasizes structural control and interface design. In contrast, the bottom-up strategy builds functional materials from the molecular or nanoscale level, using lignin, cellulose, or other wood-derived units as building blocks through polymerization, assembly, or in situ growth—forming tailored nanostructures with well-defined functionalities [131, 132]. Together, these nano-functionalization strategies establish wood as a multiscale nanoengineering platform, where both strategies complement each other in terms of design scale, structural hierarchy, and performance tuning, jointly enabling the precise construction and high-performance expansion of functionalized wood-based systems.

#### Top-Down Integration Strategies for Wood–Nanomaterial Composites

**Fig. 9:**
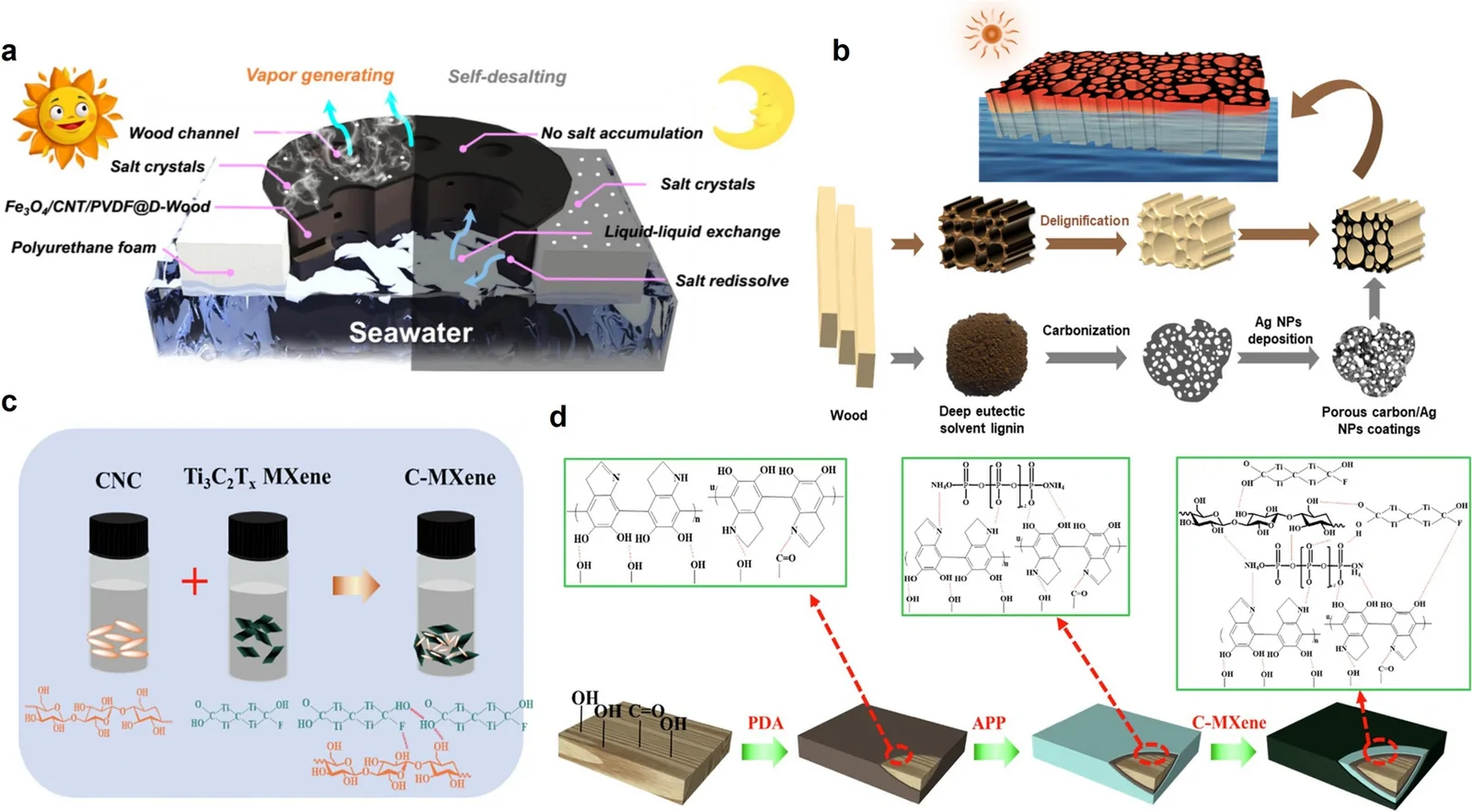
Development of wood-based composites for solar-driven water purification and integration of functional nanomaterials. **a** Honeycomb-like structure observed in chemically treated wood following freeze-drying (left) and subsequent coating with an Fe_3_O_4_/CNT/PVDF composite to enhance vapor release and water transport for seawater desalination, reproduced from Ref. [107] with permission from American Chemical Society, copyright 2024. **b** Processing of wood through delignification, carbonization, and deposition of silver nanoparticles (AgNPs), resulting in the formation of porous carbon/AgNPs coatings, reproduced from Ref. [135] with permission from Elsevier, copyright 2024. **c** Preparation of C-MXene composites via physical entanglement between cellulose nanocrystals (CNC) and Ti_3_C_2_Tₓ MXene, reproduced from Ref. [137] with permission from Elsevier, copyright 2023. **d** Surface treatment of wood through polymerization of L-Dopa, incorporation of ammonium polyphosphate (APP) and C-MXene composites, followed by structural reorganization at the molecular level, reproduced from Ref. [137] with permission from Elsevier, copyright 2023

Top-down strategies for wood functionalization involve the direct modification of bulk wood substrates, leveraging their hierarchical porous structures to accommodate and stabilize nanomaterials [41]. These strategies encompass approaches ranging from traditional surface coatings to sophisticated nanoscale engineering, aimed at enhancing physical, chemical, and interfacial properties [133]. Surface modification represents a primary method within the top-down functionalization spectrum, providing a platform for the attachment of nanomaterials at the wood interface to modulate its physicochemical characteristics. Nanostructures can be uniformly deposited through various techniques, imparting functionalities such as controlled wettability, photothermal conversion, and chemical responsiveness. As illustrated in Fig. 9a, Fe_3_O_4_/CNT nanomaterials combined with polyvinylidene fluoride (PVDF) via brushing were utilized to fabricate a magnetic photothermal wood film exhibiting asymmetric wettability for solar-driven desalination [107]. Similarly, chitosan–silica immersion coatings have been applied to enhance wood durability under marine exposure [134]. Advanced deposition techniques allow for more precise nanomaterial integration; for instance, as shown in Fig. 9b, Ag nanoparticles were loaded onto lignin-derived porous carbon pretreated with DES via a photoreduction method, enabling the creation of a bilayer solar steam generator [135]. Furthermore, plasma magnetron sputtering was employed to deposit aluminum nanoparticles onto ultrasonicated poplar substrates, achieving superhydrophobicity through the formation of nanoclustered air-trapping structures [136]. As demonstrated in Fig. 9c, d, layer-by-layer (LBL) assembly utilizing polydopamine as an adhesive facilitated the stabilization of CNC/MXene coatings, imparting multifunctional properties including fire alarm activation, smoke suppression, and volatile organic compound (VOC) removal [137].

As strategies for wood functionalization, in situ growth processes involve the direct formation of functional materials within the wood matrix, leveraging its highly porous architecture and reactive surface chemistry to generate new functional materials [10]. A wide variety of nanomaterials have been developed through this approach, including both metal and metal oxide nanoparticles (e.g., CoFeO_x_, Fe_3_O_4_), conductive polymers (e.g., PPy, polythiophene), metal–organic frameworks (MOFs), covalent organic frameworks (COFs), including atomically dispersed catalysts such as the Fe–N₃ site [138]. The above formation of all the nanostructures underwent mechanisms related to redox deposition, polymerization, coordinated assembly, and pyrolysis with a precursor that can create tightly bonded hybrid interfaces with improved structural integrity.

**Fig. 10:**
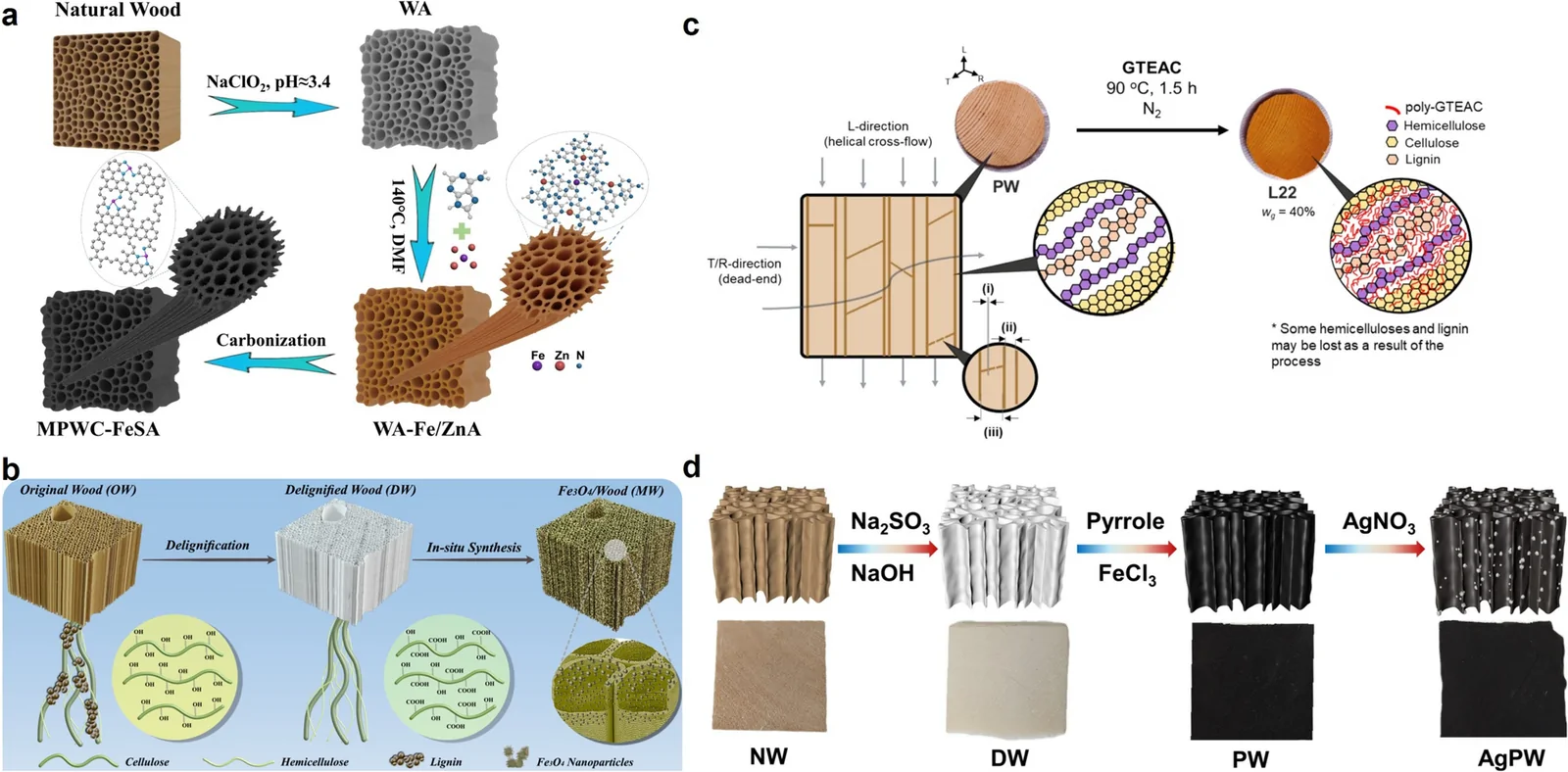
Representative in situ strategies for embedding functional nanomaterials into wood structures. **a** Construction of mesoporous wood carbon with atomically dispersed Fe–N₃ sites (MPWC–FeSA) and WA–Fe/ZnA composites via adenine-assisted metal coordination and carbonization, reproduced from Ref. [139] with permission from Elsevier, copyright 2025. **b** In situ synthesis of Fe_3_O_4_ nanoparticles within delignified wood, forming magnetic cellulose frameworks, reproduced from Ref. [106] with permission from Elsevier, copyright 2025. **c** Formation of poly-GTEAC networks through in situ polymerization of reactive ionic liquids in pine wood, enabling partial lignin/hemicellulose removal and polymer embedding, reproduced from Ref. [140] with permission from Elsevier, copyright 2025. **d** Fabrication of photothermal Ag/PPy-functionalized wood via sequential silver ion reduction and pyrrole polymerization, reproduced from Ref. [141] with permission from Elsevier, copyright 2025

Single-atom anchoring offers high-quality catalytic sites, while anchoring isolated metal species to wood-derived carbon with defects is advantageous as it utilizes its highly hierarchical porosity and the availability of vacancies to ultimately obtain metal–carbon coordination structures with strong interactions. In Fig. 10a, Sun et al. [139] have implemented a cascade strategy to anchor Fe single atoms as Fe–N_3_ sites in a delignified wood-derived carbon aerogel, through adenine-assisted coordination and defect engineering induced by Zn during pyrolysis. In situ deposition of metal oxides is valuable for hybridization of patterned nanostructures in wood-derived carbon. As shown in Fig. 10b, Long et al. [106] implemented in situ deposition of Fe_3_O_4_ nanoparticles that provided a uniform loading of nanoparticles into the lumens and cell walls of delignified wood to obtain an embedded magnetic composite of MW that had a high loading content of 35.89 wt%. The embedded MW composite indicated high Pb^2+^ adsorption capacity (537.63 mg g^−1^) and excellent magnetic responsiveness. The mechanism of adsorption proposed a synergistic effect of the –COOH, –OH, and Fe–O groups through the pseudo-second-order kinetic model and Langmuir isotherm model. Bimetallic alloy embedding can be useful for improving electrocatalysis by providing asymmetric electronic structures. The in situ embedding of CoM (M = Ni, Fe, Mn, Cu) nanoparticles, which Xu et al. [142] performed, was able to simultaneously grow carbon nanotubes to also provide a conductive 3D network and demonstrated high conductivity and charge transfer. The introduction of reactive ionic liquid (RIL) polymerizations is an ecofriendly and efficient methodology for the functionalization of wood. The RILs are polymerizable quaternary ammonium compounds which can be introduced to the wood matrix and can grow cationic polyelectrolytes in situ via nucleophilic addition to the hydroxyl groups of the hemicellulose. As shown in Fig. 10c, Ahmed et al. [152. developed GTEAC as an RIL to quaternize pine-derived wood membranes in a one-pot, solvent-free reaction conducted at 90 °C for 1.5 h, producing in situ poly-GTEAC chains covalently bound to cellulose, hemicellulose, and lignin. Composite in situ growth introduces several functional materials into wood structures, using polymerization and metal reduction for synergistic assembly. As observed in Fig. 10d, Lu et al. [141] developed a hydrogen-bond-stabilized uniform photothermal layer on delignified balsa wood by in situ polymerizing PPy. Then PPy was used to reduce silver ions to Ag nanoparticles in situ, forming the Ag/PPy composite photothermal layer.

Overall, these collectively illustrate the structural versatility and chemical compatibility of wood as a reactive scaffold for in situ nanomaterial growth. The aforementioned studies highlight the possibility of embedding reaction pathways through single-atom anchoring, oxide nanoparticle deposition, bimetallic alloy inclusion, ionic liquid polymerization, and composite polymer–metal inclusion, which demonstrate highly controlled spatial distribution, strong interfacial binding, and multifunctional coupling throughout wood. It is expected that the interweaving of atomic-precision design, defect engineering, and green synthesis pathways will propel the development of in situ routes towards programmable, scalable, and sustainably manufactured wood-based nanocomposites for next-generation energy, environmental, and electronic applications.

#### Bottom-Up Assembly of Functional Nanocomposites in Wood Matrix

In contrast to top-down methods that change the overall bulk wood, bottom-up methods create functional composites using the sub-units within wood, namely cellulose, hemicellulose, and lignin, by extracting, chemically modifying, and reorganizing those sub-units [143, 144]. These nanoscale methods create a more flexible way to control component identities, morphologies, and interfacial interactions during the addition of nanomaterials like nanoparticles, nanosheets, or molecular frameworks into synthesized or novel biomass-derived matrices. As such, using bottom-up strategies, wood-derived materials can be synthesized and designed to personalize and improve mechanical, electrical, and catalytic properties through environmentally sustainable and scalable processes that integrate advanced processing capabilities together with greater compatibility [145, 146].

**Fig. 11:**
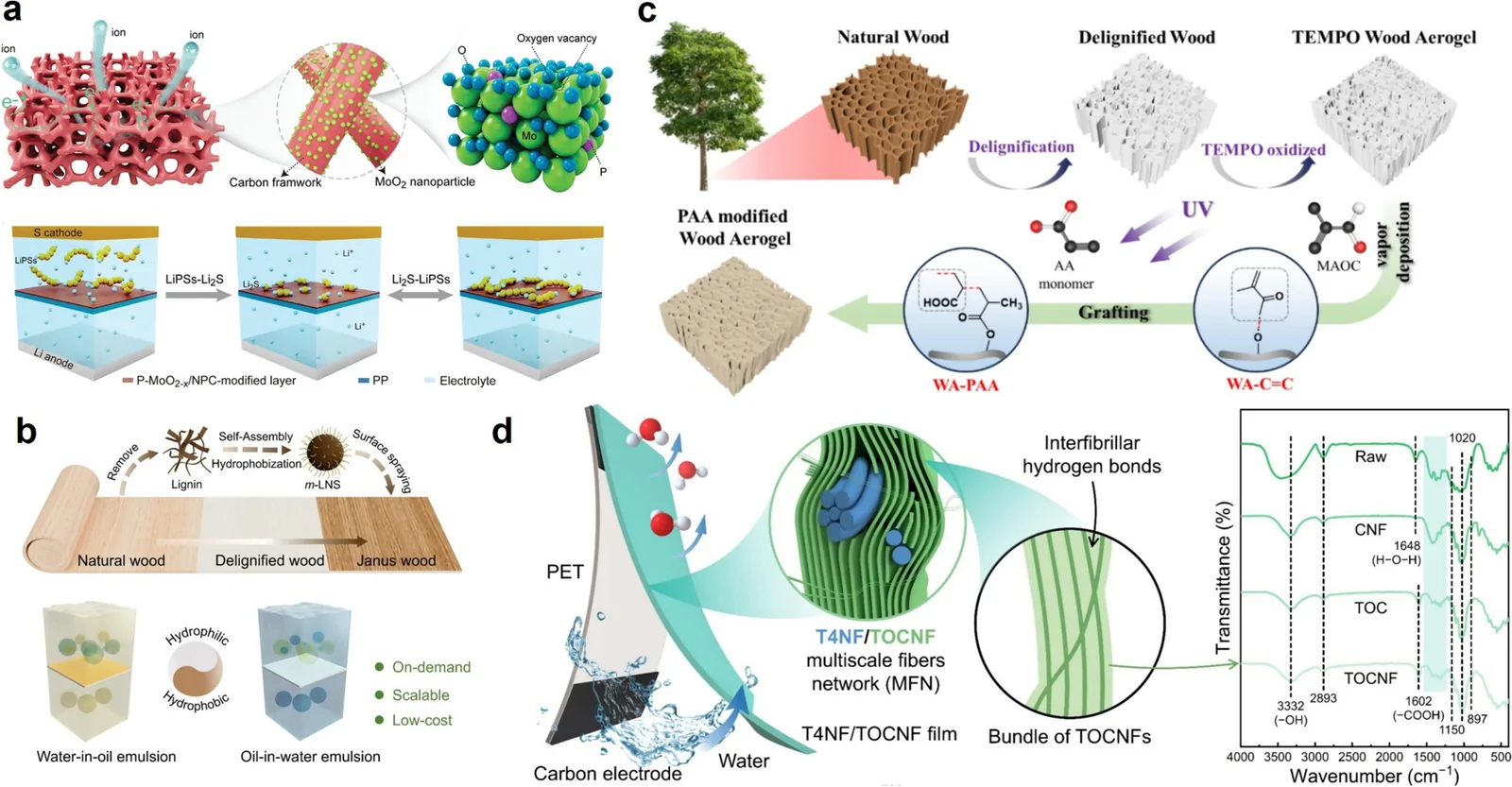
Representative bottom-up strategies for wood–nanomaterial composites. **a** Construction of a nanocellulose-templated three-dimensional carbon skeleton integrated with in situ grown phosphorus-doped MoO_2_ nanoparticles, enabling enhanced conductivity and sulfur retention for high-performance lithium–sulfur batteries, reproduced from Ref. [147] with permission from Wiley–VCH, copyright 2025. **b** Emulsion-based separation approach utilizing interfacial engineering between natural and delignified wood, achieving efficient oil–water separation by exploiting anisotropic wettability and surface energy contrasts, reproduced from Ref. [109] with permission from Elsevier, copyright 2025. **c** Fabrication of wood-derived aerogels via UV-induced polymerization, resulting in lightweight, porous structures with tunable surface functionalities suitable for applications such as thermal insulation and environmental remediation, reproduced from Ref. [148] with permission from Elsevier, copyright 2024. **d** Development of Janus wood membranes through delignification, plasticization, and asymmetric surface treatments, offering directional water transport and multifunctional separation capabilities, reproduced from Ref. [149] with permission from Elsevier, copyright 2025

Nanofiber building block methods are among the most well-known bottom-up strategies and most commonly use wood-derived nanocellulose as the building block [150]. Nanofibers can enhance functional composites by offering higher crystallinity, providing a large surface area, and possessing abundant hydroxyl groups that allow for the attachment of various functional materials (such as conductive polymers or metal oxides) to construct 3D conductive frameworks [151]. These features create numerous anchor sites for nanomaterial dissolution and dispersion, while the engineered spatial constraints promote uniform distribution and improved interfacial performance. Shi et al. [147] illustrated a process in Fig. 11a, where natural cellulose, sourced from large wood planks, was downsized into nanocellulose and subsequently templated with polymerized aniline. A precursor containing molybdenum was then introduced, followed by hydrothermal treatment to generate MoO_2_ nanoparticles. Subsequent phosphorization yielded a P-doped form of MoO_2_ anchored on N,P-codoped porous carbon (P-MoO_2-x_/NPC), a hierarchical structure that effectively enhanced particle dispersion and catalytic efficiency. The incorporation of heteroatoms further modulated the electronic structure, resulting in improved conductivity, higher capacity, superior rate performance, and enhanced cycling stability for use in lithium–sulfur batteries. Lignin-based reactions and subsequent carbonization of lignin are additional examples of bottom-up strategies, wherein lignin serves as a stabilizer for electron-rich metal ions through its functional groups. Pyrolyzing lignin leads to the formation of stable carbon-based transition metal dopants or single-atom catalysts, extending applications beyond wood materials. The critical outcome of this approach is to control metal dispersion or prevent agglomeration while simultaneously valorizing lignin as a sustainable carbon source [152].

In bottom-up wood functionalization utilizing functionalization design methodologies such as graft polymerization, reactive ionic liquid (RIL) polymerization, and multicomponent interfacial assembly, experimental procedures often have the unique advantage of allowing reconstitution of the wood-derived interface at the molecular scale. In graft polymerization, there has been an emphasis on adding functional chains to biomass substrates that can modify surface reactivity and selectivity. While RIL polymerization represents a more sustainable and efficient alternative to generate cationic polyelectrolyte networks in wood microstructures. Multicomponent assembly can produce hierarchal assemblages, or Janus architecture, composed of various components based on lignin and cellulose. As shown in Fig. 11b, Liu et al. [109] prepared hydrophobic lignin-based nanospheres (m-LNS) through self-assembly and fluorosilane modification, which were subsequently spray-coated onto delignified wood surfaces. The end result yielded a Janus membrane exhibiting a superhydrophobic side and a superhydrophilic side. Also in Fig. 11c, He et al. [148] reported a nanostructured adsorptive membrane produced from grafting poly(acrylic acid) (PAA) onto wood-derived aerogels possessing a high surface area and a high density of carboxyl functional groups. It was indicated that in situ PAA was polymerized in the wood cell walls and the functionality of the adsorptive membranes with regards to the removal of heavy metal ions (e.g., Cu^2+^ and Pb^2+^) was determined to be maximized following the polymerization of PAA due to the balanced capacity for adsorption and cycling stability. As shown in Fig. 11d, Kong et al. [149] constructed a multiscale fiber network (MFN) by embedding Ti_4_O_7_ nanofibers into a TOCNF matrix, enhancing conductivity, porosity, and water transport. The multiscale interfacial design expanded solid–liquid contact and facilitated ion and charge transport, significantly boosting evaporation-driven electricity generation.

These examples exemplify the versatility of bottom-up strategies for wood-derived material engineering through molecular-level interface engineering. Functional composites have been developed by employing graft polymerization, RIL polymerization, and multicomponent assembly, enabling control over wettability, adsorption, and reactivity. As the area of bottom-up wood functionalization progresses towards scaled-up integrated systems based on bio-derived polymers, stimuli-responsive interfaces, and engineered nanostructures, wood polysaccharides are advancing rapidly as functional materials. Such advancements are broadening the application landscape of wood-based materials, particularly in environmental remediation, energy storage, and smart membranes, all areas that require multifunctional performance and structural versatility.

## Applications of Functionalized Wood Materials

### Energy Storage

Functionalized wood serves as a structurally adaptive and chemically versatile platform that enables its integration into both battery and supercapacitor systems—two electrochemical technologies distinguished by fundamentally different charge storage mechanisms [153]. Batteries typically rely on diffusion-limited, faradaic redox reactions involving bulk ion intercalation or conversion processes, yielding high energy densities suitable for long-duration energy supply. In contrast, supercapacitors operate via non-faradaic or surface-confined faradaic processes, offering ultrafast charge–discharge dynamics and superior power density [154]. Despite these mechanistic distinctions, both systems benefit from architectures that promote efficient ion transport, structural stability, and interfacial reactivity. The hierarchical porosity, aligned channels, and modifiable surface chemistry of wood provide a unifying scaffold that can be selectively tuned to accommodate the kinetic demands of supercapacitors or the capacity requirements of batteries, thereby bridging their functional divergence through rational material design.

#### Wood-Based Electrodes for Metal-Ion Batteries

Metal–air batteries operate by utilizing metal oxidation and oxygen reduction reactions for energy storage, and in theoretical evaluations exhibit energy densities that are 3–30 times higher than those of lithium-ion batteries [155]. The advantage of metal–air batteries is that they do not require a fully closed architecture and can scavenge for oxygen from the macro-environment [156, 157]. They are similar to lithium-ion batteries with respect to their exceedingly low cost in terms of weight, size, and material costs [158]. There are numerous types of metal–air batteries, and rechargeable zinc–air batteries are considered commercially viable due to their low cost, abundance of resources, and intrinsic safety [159]. The practical application of zinc–air batteries is largely limited by the slow kinetics of the oxygen reaction occurring at the air electrode [160]. As a result, the energy efficiency associated with round-trip charges is measured at 55–65 percent, limiting the overall power output. The cycling stability is also limited due to corrosion that occurs within the moving alkaline environment, generally correlating to fewer than 500 cycles [161]. Greater attention has been placed on the development of advanced air electrode architectures as sustainability concerns increase [162]. In this case, the application of natural wood resources has been recognized as a favorable alternative given their renewable characteristics and intrinsic hierarchical porous structure supporting electron transport and gas/ion diffusion in the air electrode [163–166]. Upon carbonization of either the phase change material or electroactive component of the system, wood-derived frameworks can exhibit high surface area and a large number of active sites [79]. This makes conductive wood an attractive spatial structure host for metal nanoparticles, heteroatom doping (nitrogen, phosphorus, and sulfur), and anchoring single-atom catalysts. Functional alterations greatly improve the catalytic activity of the air electrode, leading to clean, lightweight, efficient, and resilient metal–air battery systems.

**Fig. 12:**
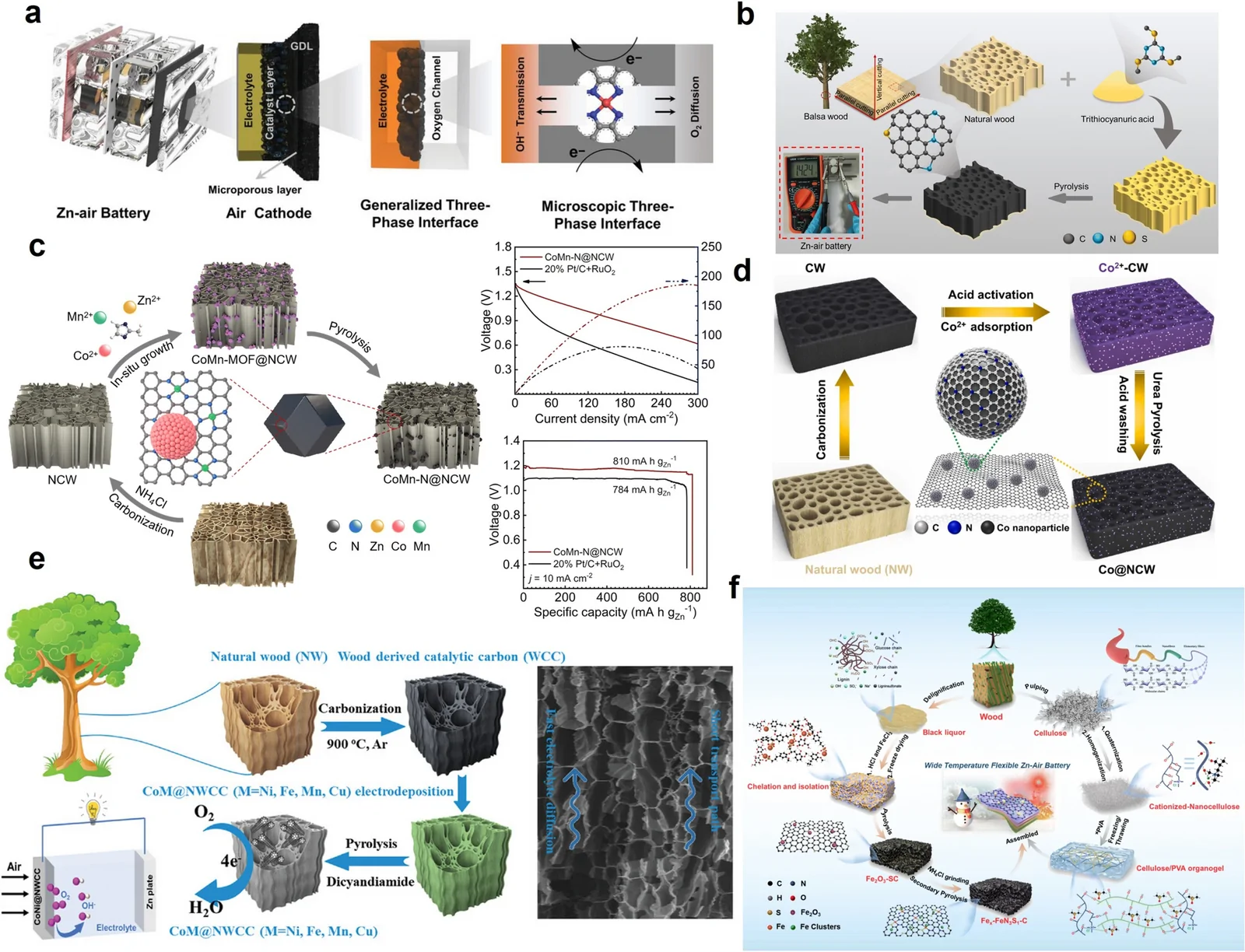
Schematic illustrations of bottom-up strategies for wood–nanomaterial composites. **a** Constructing carbonized wood electrodes with in situ CoM (M = Ni, Fe, Mn, Cu) nanoparticles for enhanced ORR in zinc–air batteries, reproduced from Ref. [158] with permission from John Wiley and Sons, copyright 2024. **b** Incorporating zinc-based MOFs into regenerated wood via solution regeneration and TEMPO oxidation for improved CO_2_ capture, reproduced from Ref. [167] with permission from Royal Society of Chemistry, copyright 2024. **c** Fabricating flexible X-ray scintillator films by vacuum impregnating Cs_3_Cu_2_I_5_ into delignified compressed wood, reproduced from Ref. [166] with permission from John Wiley and Sons, copyright 2024. **d** Forming Co/CoO heterostructures on nitrogen-doped carbonized wood for efficient zinc–air battery electrodes, reproduced from Ref. [168] with permission from John Wiley and Sons, copyright 2024. **e** Synthesizing MoNi_4_-loaded porous carbonized wood via hydrothermal treatment and carbonization for water splitting, reproduced from Ref. [142] with permission from John Wiley and Sons, copyright 2022. **f** Designing photothermal wood evaporators with hydrophobic high-entropy alloy layers for solar steam generation and salt rejection, reproduced from Ref. [152] with permission from Royal Society of Chemistry, copyright 2024

Wood is an attractive and sustainable medium for fabricating high-performance air electrodes in Zn–air batteries. Its natural channel alignment, hierarchical porosity, and renewability provide carbon frameworks for hosting heteroatom dopants, metal nanoparticles, and single-atom catalysts, enhancing oxygen reduction reaction (ORR) performance [164]. These porous structures enable rapid gas/ion transport and support catalytic stability, activity, and durability [169], making wood-derived carbon a promising lightweight and flexible electrode for next-generation metal–air batteries [165]. Optimizing catalytic interfaces and using scalable, energy-efficient synthesis can further improve performance [170]. Enhancing cycling stability and integrating with solid-state electrolytes will promote high-performance, sustainable systems. As illustrated in Fig. 12a, a zinc–air battery (ZAB) converts chemical to electrical energy via zinc oxidation and oxygen reduction, and typically consists of a zinc anode, air cathode, electrolyte, and separator [158]. The air electrode contains a current collector, gas diffusion layer (GDL), and active catalyst layer in contact with the electrolyte. Even after carbonization, natural balsa retains ordered longitudinal channels and hierarchical porosity, enabling fast electrolyte and oxygen transport.

**Fig. 13:**
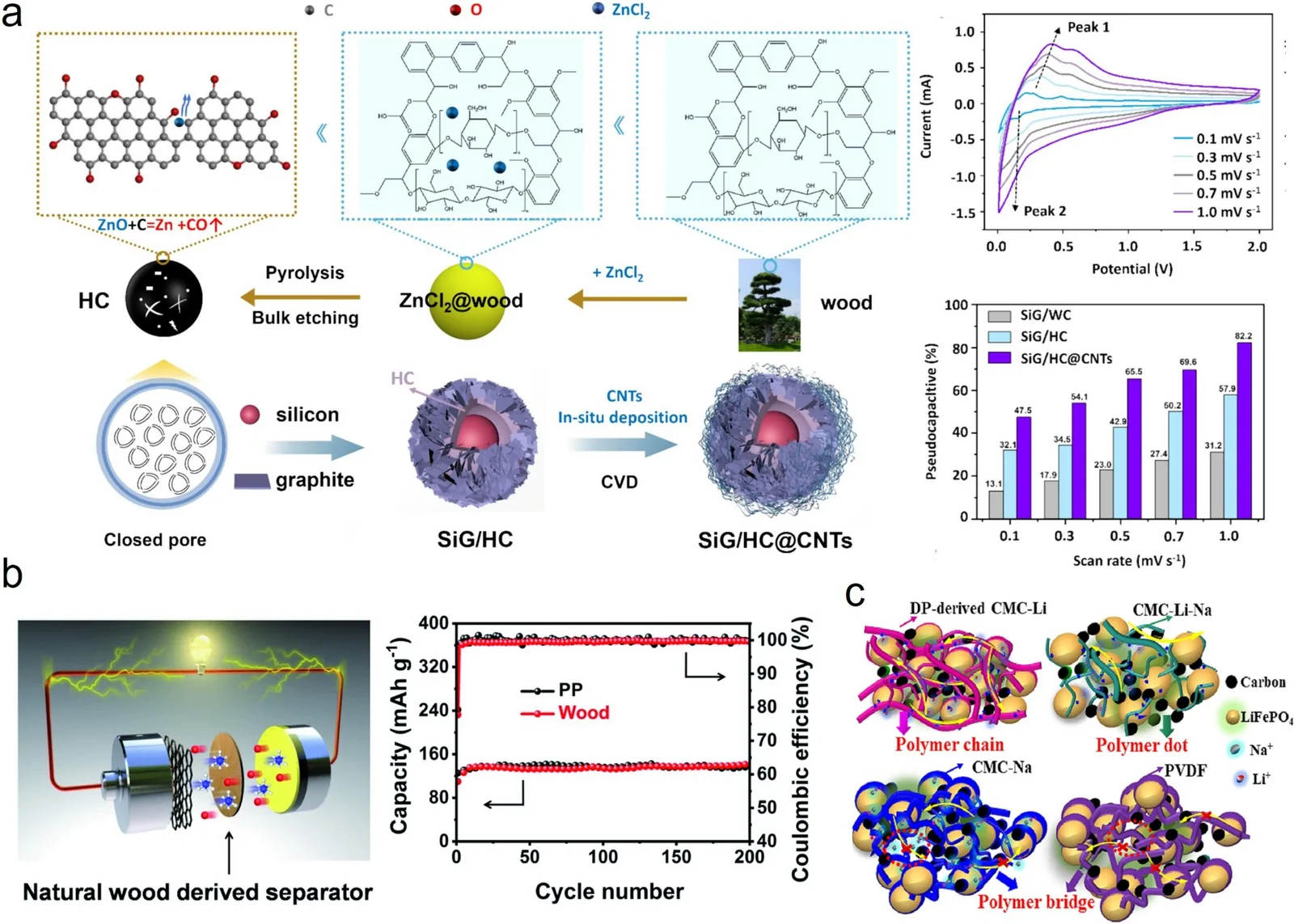
Functional applications of wood-derived materials in lithium-ion batteries. **a** Design of a composite anode of wood-derived closed-cell hard carbon and CNT wrapped micron-sized silicon (SiG/HC@CNTs) to alleviate the volume expansion of silicon and improve conductivity, reproduced from Ref. [80] with permission from Elsevier, copyright 2025. **b** Self-supporting wood-derived membrane was used as a lithium-ion battery separator, exhibiting improved mechanical stability and cyclability over commercial polypropylene separators, reproduced from Ref. [176] with permission from Royal Society of Chemistry, copyright 2025. **c** Schematic of CMC-Li binder based on wood dissolving pulp with polymeric network that coordinates the lithium ions increasing the electrochemical performance of LiFePO_4_ cathodes, reproduced from Ref. [177] with permission from Elsevier, copyright 2024

The transition from natural wood to carbonized material was achieved by simultaneously incorporating nitrogen and sulfur dopants, creating a blend of heteroatoms within the carbonized material that altered the electronic structure of the carbon matrix, thus creating distinct active catalytic sites such as pyridinic nitrogen, graphitic nitrogen, and thiophene sulfur, leading to improved ORR activity. As demonstrated in Fig. 12b, Zhang et al. [167] constructed a nitrogen and sulfur co-doped wood-derived carbon material (NSCW-900) via one-step carbonization, resulting in the incorporation of pyridinic N, graphitic N, and thiophene S into the structure, resulting in excellent ORR performance, with an onset potential of 0.93 V, a half-wave potential of 0.832 V, a diffusion current density of 4.9 mA cm^−2^, a Tafel slope of 80.54 mV dec^−1^, and excellent methanol tolerance. Beyond heteroatom doping, the presence of metal nanoparticles (NPs) on the carbonized wood-derived carbon yielded more available active sites and enabled enhanced electron transfer through the metal mixture, thereby achieving synergistic effects. As highlighted in Fig. 12c, Zhang et al. [166] produced a dual-function electrocatalyst, CoMn-N@NCW, through the anchoring of Mn-doped Co NPs onto nitrogen-doped carbon derived from cedar wood (NCW). The catalyst was produced using a co-pyrolysis process at 900 °C under an argon atmosphere, with the porous carbon skeleton originating from wood and an in situ metal–organic framework (MOF) precursor, which facilitated the uniform dispersion of metal nanoparticles. In Fig. 12d, Li et al. [168] designed a self-supporting chainmail-like electrode that contained cobalt nanoparticles encapsulated within graphitic carbon shells and uniformly embedded in nitrogen-doped carbonized wood. The ordered porous structure of the wood carbon is conducive to rapid transport of oxygen and electrolyte species, while the cobalt nanoparticles and carbon shells produce a synergistic effect that allows for enhanced electron transfer and an abundance of three-phase reaction sites. In Fig. 12e, Xu et al. [142] developed wood-derived carbon (WCC) with a hierarchical porous structure by carbonizing natural pine wood. Carbon nanotubes (CNTs) were in situ grown within the hierarchical porous carbon framework via electrochemical deposition and nitrogen-assisted pyrolysis, and asymmetric bimetallic CoM (M = Ni, Fe, Mn, Cu) nanoparticles were embedded in situ to create an integrated self-supporting electrode (CoM@NWCC). In Fig. 12f, Chen et al. [152] proposed an all-wood-based, wide-temperature flexible zinc–air battery strategy by incorporating an Fe cluster-enhanced asymmetric single-atom catalyst (Fex/FeN_3_S_1_-C) and a weather-resistant organic hydrogel electrolyte (CNF@PVA-SSE). By utilizing lignin-derived, cellulose-rich constituent species from black liquor, the researchers developed a sustainable single-atom catalyst and solid-state electrolyte. The Fe catalyst exhibited exceptional oxygen reduction performance. Metal–air batteries, such as zinc–air and lithium–air systems, are open electrochemical cells that use a metal anode and ambient oxygen as the cathodic reactant [80, 147, 178]. Their energy storage and release processes are governed by gas–electrolyte–solid three-phase reactions, with the discharge driven by the oxygen reduction reaction (ORR) and the charge involving the oxygen evolution reaction (OER). This design offers exceptionally high theoretical energy densities, but the systems often face challenges including sluggish oxygen reaction kinetics, limited cycle life, and complex air–electrode engineering [178]. In contrast, metal–ion batteries—including lithium-ion, sodium-ion, and potassium-ion batteries—are closed systems in which energy conversion occurs through the reversible intercalation and deintercalation of metal ions between the anode and cathode. Unlike metal–air batteries, no atmospheric oxygen participates in the reactions, resulting in different electrode architectures, electrolyte choices, and sealing requirements. Lithium-ion batteries (LIBs), in particular, have become dominant in electric vehicles and renewable energy storage due to their high round-trip efficiency, long cycle life, and mature manufacturing infrastructure [179]. Lithium-ion batteries (LIBs) are critical components in electric vehicles and renewable energy storage systems [180–182]. Wood-based hard carbon materials are gaining interest in LIB applications due to their excellent electrochemical performance and sustainability [183]. Specifically, wood-derived closed-cell hard carbon limits the volume expansion of silicon-based anodes during charge–discharge cycles via its pore structure. Carbon nanotubes (CNTs) can further improve the structural stability and conductivity of silicon composites, thus enhancing the overall performance of the battery [184]. As shown in Fig. 13a, Gao et al. [80] designed an anode composed of wood-derived closed-cell hard carbon and CNT-wrapped micron-sized silicon (SiG/HC@CNTs) to mitigate the volume expansion of silicon and achieved a reversible capacity of 750 mAh g^−1^ at 0.2 A g^−1^ with 91.21% capacity retention after 500 cycles. As shown in Fig. 13b, Yang et al. [176] developed a wood-derived self-supporting membrane that served as an efficient separator, exhibiting excellent mechanical strength, high ionic conductivity, and thermal stability. As shown in Fig. 13c, Li et al. [177] described a direct synthesis process for a carboxymethyl cellulose-lithium (CMC-Li) binder derived from wood dissolving pulp that improved the electrochemical performance of LiFePO_4_ cathodes. The role of wood products is transitioning in lithium-ion batteries from a simple carbon source to a multiuse structural tuning vehicle. Wood-derived closed-cell hard carbon helps provide stable anchoring positions and limit the volume expansion of silicon, while facilitating stable electron and ion transport through reconstructed conductive networks. Wood separators and binders, by regulating the orientation of the fibers and the configuration of the functional groups, provide good thermal stability and interfacial compatibility. Further research is needed to elucidate programmable transformation mechanisms of wood microstructures and develop multi-scale electrode systems for synergistic functionality in structurally integrated high-energy–density batteries [185].

**Fig. 14:**
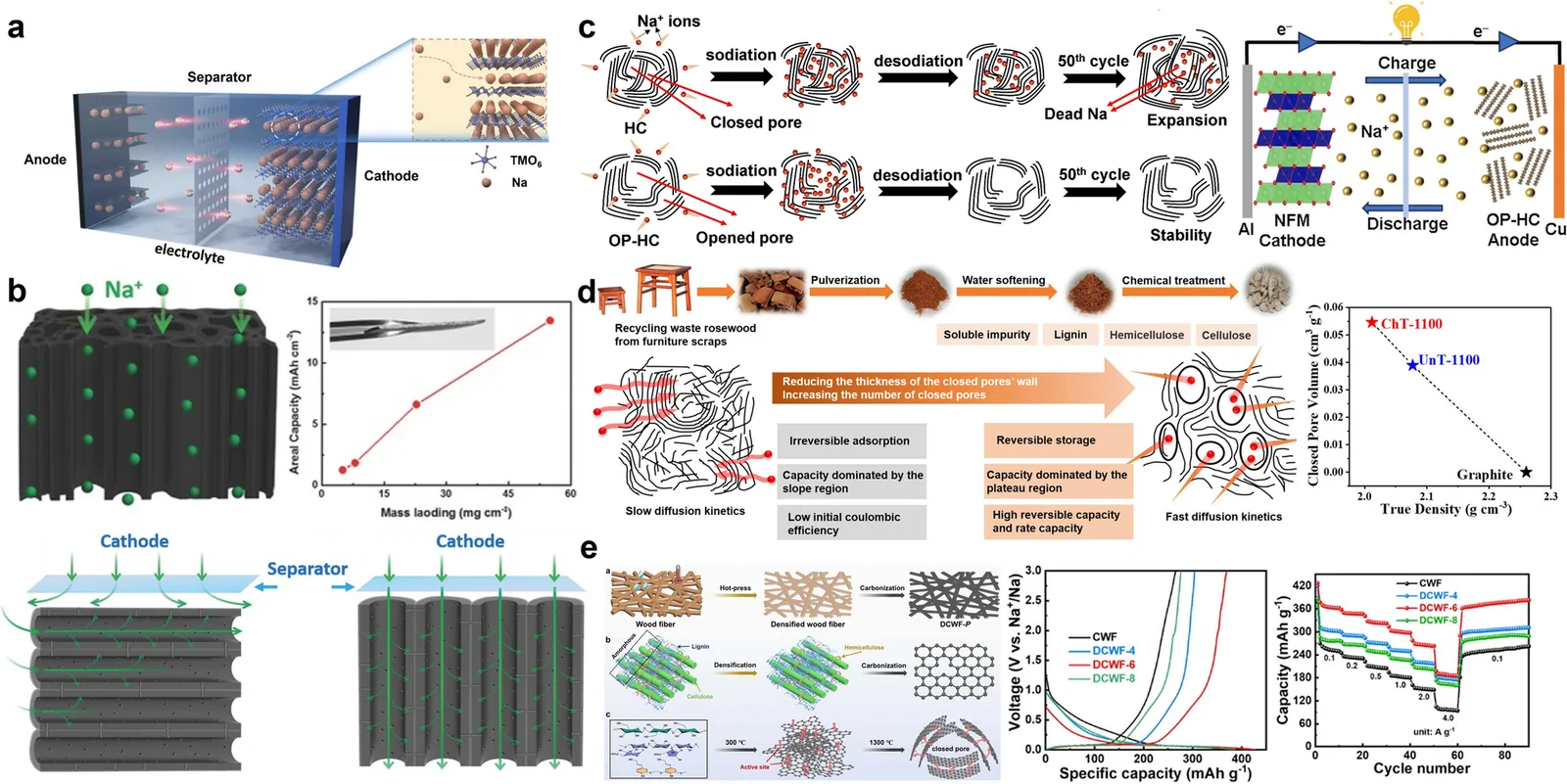
Applications of wood-derived carbon materials in sodium-ion batteries. **a** Schematic structure of a sodium-ion battery using wood as carbon-derived anode material showing ion transport across the separator and electrode interfaces, reproduced from Ref. [178] with permission from Elsevier, copyright 2025. **b** Hierarchical porous structure of carbonized wood that promotes rapid sodium ion diffusion, taken alongside the rate performance, reproduced from Ref. [76] with permission from Royal Society of Chemistry, copyright 2024. **c** Impact of closed-pore and open-pore hard carbon architectures of wood on cycling stability and sodium storage performance, reproduced from Ref. [4] with permission from John Wiley & Sons, copyright 2022. **d** Respect to cellulose crystallinity changes the carbonized wood microstructures ion storage performance, reproduced from Ref. [186] with permission from Elsevier, copyright 2025. **e** Structural evolution of wood-derived carbon through treatments and their effect on performance in electrochemistry in specific capacity and cycling stability, reproduced from Ref. [81] with permission from Royal Society of Chemistry, copyright 2024

Sodium-ion batteries (SIBs) offer a low-cost and sustainable option compared to lithium-ion batteries (LIBs) [187]. This is due to the abundant resources of sodium with the use of aluminum current collectors [188]. Although sodium has a larger ionic radius, its chemistry is similar to lithium allowing for a rocking-chair mechanism [189–191]. Wood-derived hard carbon has become a promising anode material in SIBs based on its tunable pore structure, increase in interlayer spacing, and good conductivity [192]. Closed pores can improve the low-voltage plateau capacity, open pores can enhance ion diffusion, and natural wood channels could allow for low tortuosity, thick electrodes [4]. Together, this offers green pathway towards high-performance SIBs. As shown in Fig. 14a, sodium-ion batteries (SIBs) work in a rocking-chair manner by utilizing sodium ions shuttling between a hard carbon anode film and a sodium-containing cathode film typically consisting of layered transition metal oxides (TMO_6_) [178]. The carbon anode can store sodium ions within its matrix and the electrolyte simulates ion transport while the separator separates the anode and cathode and prevents short circuits.

As depicted in Fig. 14b, Shen et al. [76] built an ultra-thick, low tortuosity mesoporous carbon anode straight from the thermal treatment of natural wood and obtained its final film state followed the vertically aligned natural wood channel structure. This design greatly improved the performance associated with sodium-ion batteries. The resultant electrode that exhibited a thickness of 850 μm and areal mass loading of 55 mg cm^−2^ reported a high areal capacity of 13.6 mAh cm^−2^. The film maintained excellent capacity and stability at a current density of 0.55 mA cm^−2^ that far exceeded the performance of commercially available graphite anodes. As shown in Fig. 14c, a derived open-pore hard carbon (OP-HC) anode material was synthesized by using waste wood and PVP-assisted carbonization to form a porous structure with increased interlayer spacing (d₀₀₂ = 0.414 nm) [4]. This structure allowed for a high reversible capacity of 350.7 mAh g^−1^ at 0.05 C and a high initial Coulombic efficiency (ICE) of 94.9%. The OP-HC also showed a good capacity of 204.8 mAh g^−1^ when cycled at a high rate of 3.0 C, and after 500 cycles at 1.0 C, the OP-HC had a good capacity of 245.2 mAh g^−1^ with good cyclic stability. The OP-HC was found to have much improved rate capability and cycling performance compared to a conventional closed-pore hard carbon (HC). As shown in Fig. 14d, Zhou et al. [186] also created a closed-pore structured hard carbon through selective removal of lignin and hemicellulose, allowing for low-temperature pyrolysis (1100 °C). The optimized sample (ChT-1100) contained closed pores that were thinner-walled as well as a larger interlayer spacing of 0.386 nm, and a greater pore volume (0.055 cm^3^ g^−1^), leading to excellent sodium storage properties with a capacity of 326 mAh g^−1^ at 20 mA g^−1^ and 230 mAh g^−1^ at 5000 mA g^−1^. Figure 14e shows Chen et al. [81] incorporated a heat-pressing method to modify the wood structure before carbonization to produce a hard carbon containing an abundance of closed pores and increased interlayer spacing. The optimized sample (DCWF-6) gave a reversible capacity of 427.1 mAh g^−1^, an ICE of 86% and a retained capacity of 197.7 mAh g^−1^ at a rate of 4.0 A g^−1^. The improved capacity was linked to cellulose reorganization and carbon layer alignment, which increased plateau capacity.

Functionalized wood, as a platform with both programmable structural architecture and tunable chemical properties, demonstrates remarkable performance advantages and sustainability potential across diverse electrochemical energy systems [4, 178, 189]. In metal–air batteries, carbonized wood frameworks preserve the natural longitudinal channels and hierarchical porosity of raw wood, enabling simultaneous rapid gas/liquid/ion transport and the construction of high-surface-area catalytic sites [152]. Nitrogen–sulfur co-doping introduces catalytically active sites such as pyridinic N, graphitic N, and thiophene S (e.g., onset potential of 0.93 V, half-wave potential of 0.832 V, and diffusion current density of 4.9 mA cm⁻^2^), while the in situ anchoring of Co, Mn, Ni nanoparticles or single-atom catalysts significantly enhances oxygen reduction reaction (ORR) kinetics, catalytic durability, and round-trip efficiency, resulting in lightweight, high-power-density, and durable Zn–air battery systems. For lithium-ion batteries, wood-derived closed-pore hard carbon effectively buffers the volumetric expansion of silicon-based anodes during cycling, while providing stable electron/ion transport pathways; its integration with carbon nanotubes forms a three-dimensional conductive network that delivers a reversible capacity of 750 mAh g^−1^ with 91.21% capacity retention after 500 cycles at 0.2 A g^−1^. Furthermore, wood-based separators and wood-derived binders, through control of fiber alignment and functional group chemistry, achieve excellent thermal stability, electrolyte wettability, and interfacial compatibility. In sodium-ion batteries, wood-derived hard carbon enables optimization of ion diffusion kinetics and low-voltage plateau capacity via control of open/closed pore ratios and interlayer spacing (e.g., 0.386–0.414 nm for closed-pore structures); closed pores improve low-voltage plateau capacity and cycling stability, while open pores enhance rate capability. Low-tortuosity, ultra-thick electrodes templated directly from natural wood channels (thickness up to 850 μm, areal capacity of 13.6 mAh cm^−2^) exhibit superior areal capacity and cycling stability compared to commercial graphite anodes. Collectively, functionalized wood enables precise structure–property synergy across different electrochemical platforms, providing a feasible pathway for the deep integration of sustainable materials with high-performance energy storage devices. Future efforts should focus on scalable, low-energy manufacturing strategies, atomic-level construction of catalytic interfaces, and integration with solid-state electrolyte systems to achieve long-term stability, cost-effectiveness, and environmental compatibility, thereby accelerating the transition of wood-based electrochemical technologies from laboratory prototypes to commercial applications.

#### Functional Wood Electrodes for Supercapacitors

**Fig. 15:**
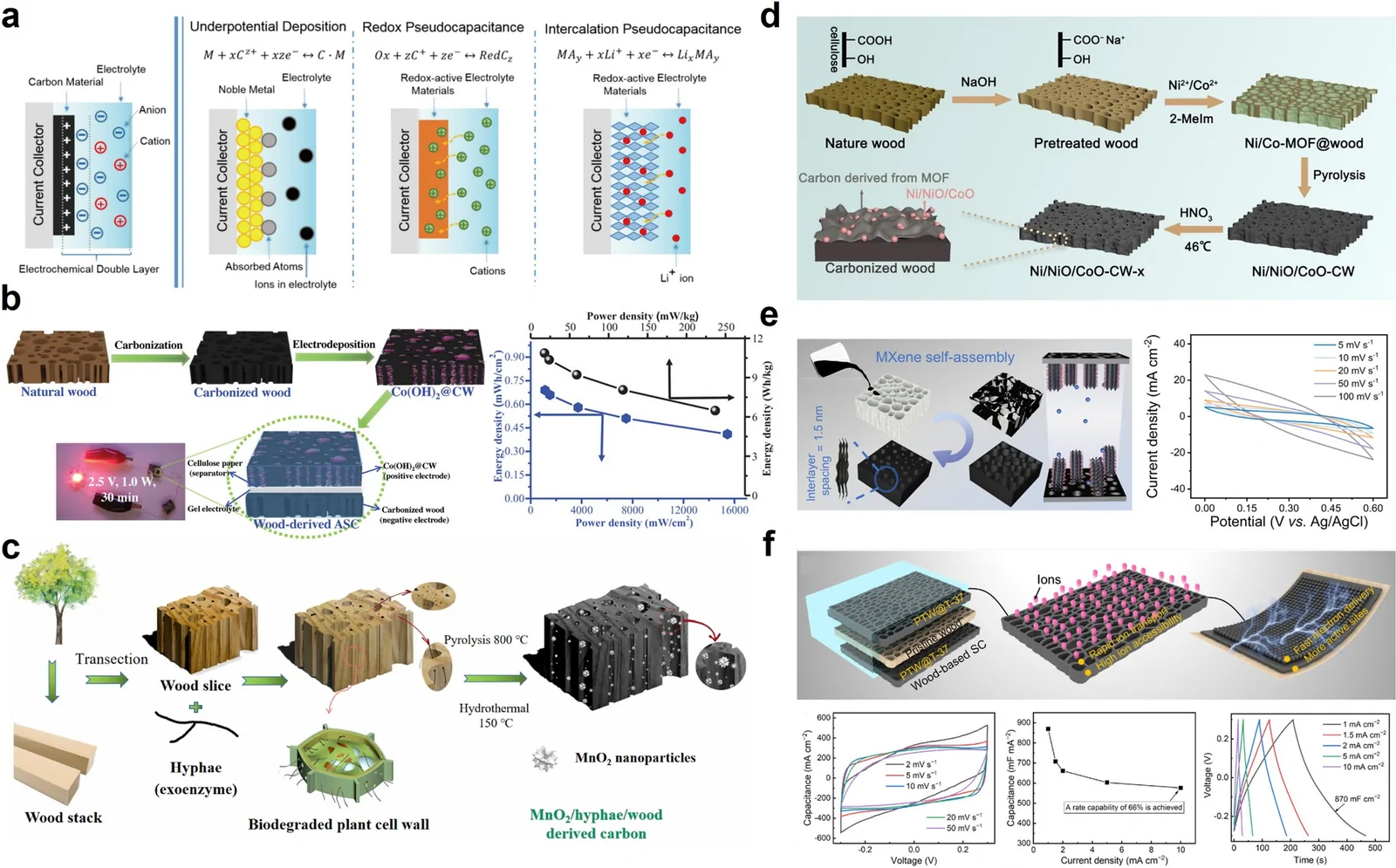
Wood-derived carbon architectures for supercapacitor and hybrid energy storage applications. **a** Electric double-layer capacitance, redox pseudocapacitance, and intercalation pseudocapacitance achieved by wood-derived carbon electrodes, reproduced from Ref. [193] with permission from Elsevier, copyright 2023. **b** Formation of hierarchical porous carbon through carbonization and ZnCl_2_ activation, followed by MnO_2_ deposition, reproduced from Ref. [195] with permission from Elsevier, copyright 2022. **c** Production of MoO_2_/hyphae/wood-derived carbon via fungal biodegradation and pyrolysis, reproduced from Ref. [196] with permission from Elsevier, copyright 2023. **d** Construction of Ni/Co bimetallic oxide–MOF composites on chemically pretreated wood to enhance conductivity and active site density, reproduced from Ref. [197] with permission from Elsevier, copyright 2024. **e** Self-assembly of MXene nanosheets on carbonized wood to fabricate high-rate electrodes with improved redox activity, reproduced from Ref. [100] with permission from Wiley–VCH, copyright 2022. **f** Fabrication of flexible asymmetric supercapacitors using wood carbon for high cycling stability and mechanical flexibility, reproduced from Ref. [198] with permission from Wiley–VCH, copyright 2018

A supercapacitor combines the best features of a conventional capacitor and a conventional battery into a single device that has high capacitance, fast charge/discharge rates, long cycle life, and is environmentally friendly [7]. Supercapacitors fall into diverse classifications of supercapacitor types—including electric double layer capacitors (EDLCs), pseudocapacitors (PCs), and hybrid supercapacitors (SCs)—as shown in Fig. 15a. Each type of supercapacitor employs distinct mechanisms for energy storage, but shares the same basic components: active electrodes, ion-conducting electrolytes, and separators [193]. EDLCs store charge via physical adsorption at the electrode–electrolyte interface, forming an electric double layer. In contrast, PCs (pseudocapacitors) utilize fast and reversible redox reactions or ion intercalation/de-intercalation to store charge, while hybrid SCs (supercapacitors) combine carbon-based electrodes from EDLCs and metal oxide or conducting polymer electrodes from PCs, achieving superior energy and power densities [194]. A supercapacitor consists of electrodes, a separator, and an electrolyte. The electrodes contain active materials and a current collector for charge transfer. The separator is porous and optimally sized to enhance ion transport while preventing short-circuiting. The electrolyte facilitates charge exchange between ions and active materials and must be chemically compatible with the current collector. The hierarchical porous structure of wood provides channels for efficient ion transport and spaces for ion storage. During biochar production or carbonization, wood retains mechanical strength from the cellulose framework, allowing transformation into conductive electrode materials via carbonization and/or functionalization. Carbonization and pyrolysis, involving controlled thermal decomposition, spatially transform natural wood into graphitized carbon. In situ graphitization reduces the internal resistance of the electrode. Additional porosity, resulting from internal pressure during charring and polymer decomposition, enhances ion transport, indicating that carbonized wood has significant potential as an electrode material for energy storage devices. As shown in Fig. 15b, a high-performance all-solid-state asymmetric supercapacitor was fabricated using wood-derived multilayer porous electrodes [195]. Carbonized wood was used as the negative electrode, and Co(OH)_2_ was electro-deposited to form the positive electrode. Figure 15c demonstrates the use of white-rot fungus biological treatment to optimize the pore structure of wood-based carbon and improve supercapacitor electrode performance [196]. Following carbonization and MnO_2_ hydrothermal deposition, the HWC-3 M electrode achieved an areal specific capacitance of 3395 mF cm^−2^, a gravimetric specific capacitance of 138.3 F g^−1^, and good cycling stability with a capacitance retention of 88.6%.

In addition to carbonization, heteroatom doping, either with O, N, P, or S, can enhance the performance of wood-derived carbon through polar covalent bonding and intentional introduction of structural defects (due to varying electronegativities). This results in improved wettability at the electrode–electrolyte interface, thereby enhancing specific capacitance. Inorganic compounds, through redox reactions and phase changes, can also function as pseudocapacitive materials, thus improving energy density, particularly metal oxides. Metal oxides are widely employed in supercapacitors due to their high electrochemical reversibility, excellent cycling stability, morphological tunability, and high theoretical specific capacitance, superior to many conventional electrode materials. Besides metal oxides, conductive polymers (such as polyaniline [PANI], PPY, polythiophene [PTH], and poly(3,4-ethylenedioxythiophene) [PEDOT]) are commonly used and are often combined with metal oxides. Their conjugated π-bond structures enable reversible redox reactions (doping–de-doping) along the polymer chains, allowing efficient charge storage and providing high theoretical specific capacitance.

Wood can still serve as a high-performance supercapacitor electrode without requiring high-temperature carbonization [199]. As revealed in Fig. 15d, Xiong et al. [197] developed the Ni/NiO/CoO-CW-4 electrode via carbonization, oxidative activation, and acid etching, achieving a high specific surface area, optimized pore structure, and excellent conductivity. It delivered an areal capacitance of 16.76 F cm^−2^ at 5 mA cm^−2^, while the assembled symmetric all-solid-state supercapacitor exhibited an energy density of 0.67 mWh cm^−2^ and a cycling stability of 96.21%. As illustrated in Fig. 15e, Yu et al. [100] employed a steam-driven self-assembly strategy to deposit MXene onto carbonization-free delignified balsa wood (DBW), constructing a three-dimensional conductive electrode and assembling a symmetrical all-wood supercapacitor. The electrode exhibited a specific capacitance of 580.55 F g^−1^ (5.16 mg cm^−2^) with stable performance over 10,000 cycles. The all-solid-state device achieved an energy density of 19.22 μWh cm^−2^ and a power density of 0.58 mW cm^−2^.As illustrated in Fig. 15f. Chen et al. [198] developed a water evaporation-induced self-assembly strategy to integrate Ti_3_C_2_ (MXene) nanosheets into non-carbonized wood, leveraging its aligned channels for high-quality MXene deposition (mass ratio up to 50%). Dopamine microspheres prevented MXene restacking, enhancing surface area and electrochemical activity. The freestanding electrode achieved an areal capacitance of 1060 mF cm^−2^ at 0.5 mA cm^−2^.

Wood-based materials for supercapacitor electrodes leverage their inherent multiscale porous architecture, excellent ion transport capability, and sustainability advantages. Carbonized wood, which retains the mechanical strength of its cellulose framework, can be engineered via pyrolysis, graphitization, and chemical activation with ZnCl_2_ to yield a high specific surface area and hierarchical porosity. Subsequent deposition of metal oxides such as MnO_2_ and Co(OH)_2_ enables high specific capacitance (e.g., 3395 mF cm^−2^, 138.3 F g^−1^) and outstanding cycling stability (88.6% capacitance retention). Heteroatom doping (N, O, P, S) enhances electrode–electrolyte interfacial wettability by introducing polar covalent bonds and structural defects, thereby improving capacitance, while the integration of metal oxides with conductive polymers (e.g., PANI, PPY, PEDOT) imparts pseudocapacitive behavior, significantly boosting energy density and rate performance. Furthermore, non-carbonized wood, modified through biodegradation, multi-step chemical treatments, or vapor-driven self-assembly, and composited with two-dimensional conductors such as MXenes, can achieve high conductivity and abundant active sites while maintaining excellent mechanical flexibility, enabling the fabrication of free-standing, flexible electrodes (e.g., 580.55 F g^−1^ with > 10,000 cycle stability). These findings demonstrate that through carbonized/non-carbonized pathways, precise pore structure regulation, heteroatom doping, and functional material integration, wood-derived electrodes can achieve high specific capacitance, high power density, long cycle life, and environmental compatibility, offering a sustainable strategy for high-performance supercapacitors and hybrid energy storage devices.

### Wastewater Treatment and Environmental Remediation

Wood's multifunctional potential for water treatment is very high and highly correlated, with many targeted applications including catalytic degradation, filtration–adsorption, and solar-driven evaporation [3]. As a natural nanoengineering platform, wood combines intrinsic micro- and nanoscale structures—including aligned cellulose microfibrils, nanofibrillar pores, and modifiable surface chemistries—that enable diverse nano-functionalization strategies tailored for water treatment [200]. In catalytic degradation, wood's embedded three-dimensional porous architecture has made it particularly suitable as a supporting material for Fenton systems, utilizing transition metals such as Fe, Cu, and Mn to promote the development of effective Fenton, Fenton-like, and photo-Fenton systems to generate reactive oxygen species (ROS, e.g., ·OH and SO_4_^−^) from H_2_O_2_ or PMS, enabling deep oxidation of challenging organic pollutants [201]. The introduction of metal-based nanocatalysts into wood's porous network represents a core nano-functionalization approach, leveraging nanoscale dispersion and strong interfacial contact [58]. Wood can serve as a metal redox cycling site, enhancing ROS production under visible and UV light through photo-excited electron generation. In the dark, wood-derived biochar with bimetallic active sites can efficiently activate PMS, enabling rapid removal of complex contaminants including antibiotics and dyes [59, 202]. For filtration–adsorption, wood's innate channel morphology provides effective physical sieving potential, enabling high-flux and selective separation of multiphase pollutants such as oil–water emulsions. Additionally, nanoscale surface engineering allows for the introduction of functional groups such as carboxyl, amino, ethylenediaminetetraacetic acid (EDTA), or MOFs, facilitating the selective chemical adsorption of heavy metals and cationic dyes [60]. Further surface functionalization with quaternary ammonium salts or cationic polyelectrolytes would impart positive surface charges that allow for efficient removal of anionic contaminants (NO_3_^−^, SO_4_^2−^), with highly acceptable fluid permeability and selectivity, enabling potential recovery and reuse of chemical entities [203].

#### Catalytic Decomposition of Pollutants Using Wood-Derived Systems

**Fig. 16:**
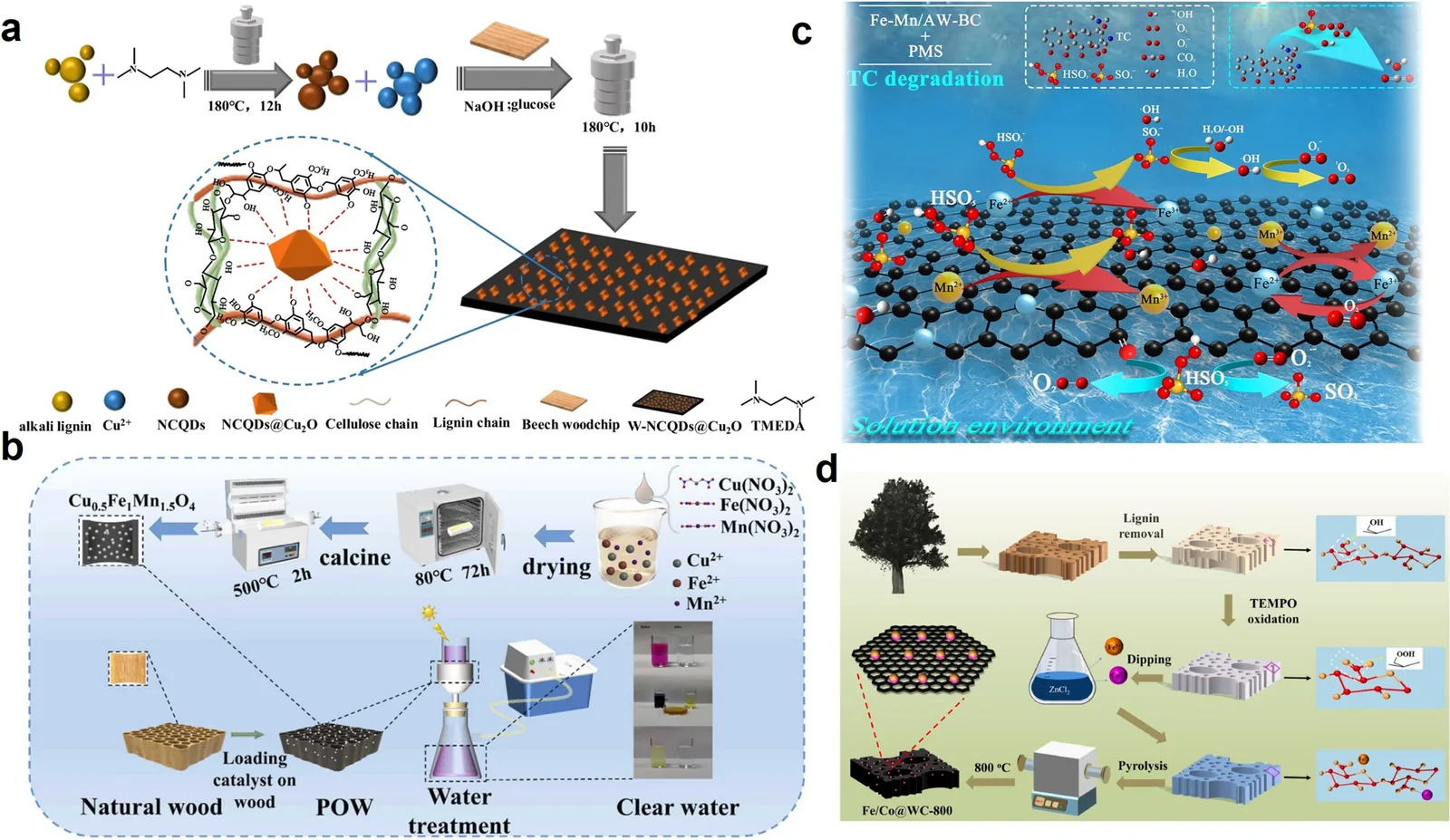
Wood-derived composite catalysts for water purification and advanced oxidation processes. **a** Hydrothermal synthesis of a lignin-based wood composite with CuFe_2_O_4_ nanoparticles for efficient oxidation of organic pollutants via Fenton-like reactions, reproduced from Ref. [210] with permission from Elsevier, copyright 2023. **b** Fabrication of porous oxidized wood loaded with CoMn_2_O_4_ and CuMn_2_O_4_ catalysts, enabling the combination of catalytic oxidation and filtration for integrated water treatment, reproduced from Ref. [211] with permission from Springer Nature, copyright 2024. **c** Construction of a Fe–Mo–Mn/wood-activated carbon composite applied in PMS-based advanced oxidation processes for efficient dye degradation through synergistic mechanisms, reproduced from Ref. [212] with permission from Elsevier, copyright 2024. **d** Preparation of a FeCo@NC/BC multifunctional catalyst derived from wood components by delignification, heteroatom doping, and carbonization, enhancing overall remediation performance, reproduced from Ref. [213] with permission from Elsevier, copyright 2023

In the area of wood-derived catalytic degradation of water pollutants, photo-Fenton and Fenton-like mechanisms are efficient advanced oxidation processes (AOPs) [204], using transition metal catalysts (Fe, Cu, Mn) supported on porous wood to degrade recalcitrant organic contaminants [193, 205–209]. With oxidants such as hydrogen peroxide (H_2_O_2_) or peroxymonosulfate (PMS), these metals generate reactive oxidative species (ROS) like hydroxyl (·OH) and sulfate (SO_4_·^−^) radicals and sustain their regeneration cycles. Light irradiation accelerates the Fe^3+^ → Fe^2+^ reduction, enhancing ROS generation and the photo-Fenton effect. As shown in Fig. 16a, Fang et al. [210] developed a wood-based membrane filtration–photo-Fenton system with CuFeMn oxide catalysts, synergistically producing ·OH and SO_4_·^−^ radicals with H_2_O_2_, while light promoted the metal redox cycle and electron–hole pair excitation. In contrast, photocatalysis generates ROS from light-excited charge carriers without external oxidants. As shown in Fig. 16b, Liu et al. [211] synthesized a W-NCQDs@Cu_2_O composite by combining nitrogen-doped carbon quantum dots (NCQDs) with Cu_2_O on wood. NCQDs reduced charge recombination and enhanced ·OH and ·O_2_^−^ generation, enabling effective methylene blue degradation.

Advanced oxidation processes in wood systems do not rely on photolysis but instead use transition metals (e.g., Fe, Mn, Cu) to activate peroxymonosulfate (PMS) or hydrogen peroxide (H_2_O_2_) and produce reactive oxygen species (ROS) under dark conditions (e.g., SO4·^−^, ·OH, ·O_2_^⁻^, and ^1^O_2_) for effective degradation of organic pollutants via redox cycling of metal ions. This form of AOP would be useful for removing a variety of contaminants (e.g., antibiotics, dyes) and requires no external light. As shown in Fig. 16c, Liang et al. [212] developed a NaOH-modified biochar-supported Fe–Mn bimetallic catalyst (Fe–Mn/AW-BC) for PMS activation, successfully removing tetracycline (TC) in the absence of light with a removal efficiency of 97.9% within 60 min. The catalyst operated by taking advantage of Fe/Mn redox cycles and surface C = O functional groups on biochar to generate multiple ROS via both radical and non-radical degradation pathways. The system exhibited great catalytic activity, broad pH adaptability, and good reusability, providing an effective strategy for non-photocatalytic wood-based advanced oxidation processes. In Fig. 16d, Pang et al. [213] fabricated a non-photocatalytic degradation system from natural wood-derived biochar. Using bimetallic Fe/Co sites embedded in NaOH-functionalized wood biochar, they created a Fe/Co@WC-800 composite that effectively activated PMS for the rapid degradation of ciprofloxacin (CIP) in the dark, achieving 100% removal within 4 min.

#### Wood-Based Filtration and Adsorption Interfaces

**Fig. 17:**
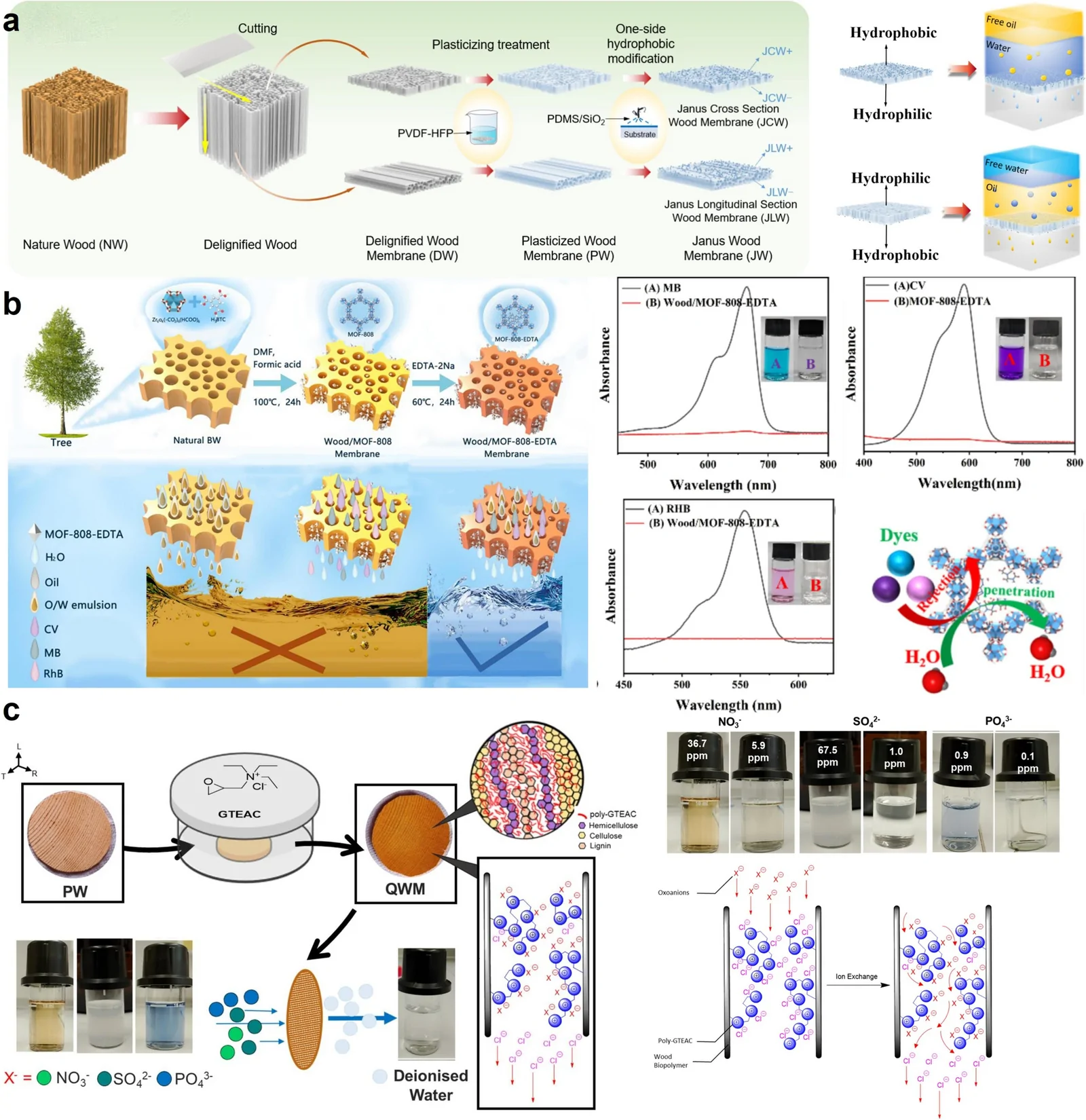
Functionalized wood membranes for water purification and ion separation. **a** Janus wood membranes fabricated via delignification, plasticization, and hydrophobic modification (PDMS or PVDF-HFP), enabling directional liquid transport and efficient oil–water separation, reproduced from Ref. [109] with permission from Elsevier, copyright 2025. **b** MOF-808–EDTA-functionalized wood membranes for dye removal and emulsion demulsification, operating via Fenton-like catalytic degradation, reproduced from Ref. [218] with permission from Elsevier, copyright 2025. **c** Quaternized wood membranes (QWM) prepared by grafting quaternary tetraethylammonium chloride(QTEAC) onto natural wood for selective anion exchange and effective removal of NO_3_^⁻^, SO_4_^2−^, and PO_4_.^3−^ from aqueous solutions, reproduced from Ref. [140] with permission from Elsevier, copyright 2025

Wood is a natural filter medium that is highly suitable for water filtration and related applications due to its three-dimensional porous architecture, derived from vessels, fiber lumens, and pits [214–216]. In the “filtration–adsorption” model, three-dimensional porous media incorporate hierarchical features, creating size-selective sieving and extended diffusion pathways for contaminants. Hence, it can serve as an efficient and effective filtering medium to separate complex oil–water emulsions. The delignification process accelerates the porosity of wood, thereby enhancing water flux and providing a substrate for coatings or functionalization [214, 217]. Wood can be transformed into Janus structures, exhibiting asymmetric wettability, with the hydrophilic side dispersing water while the hydrophobic side collects oil [216]. This structural transformation provides opportunities for selective separation of O/W and W/O emulsions. By phase separation, strategies to improve flux, selectivity, and stability can be further implemented to meet the requirements of field applications, whether for oil–water emulsion separation or contaminated industrial wastewater treatment, as demonstrated in Fig. 17a. Liu et al. [109] used delignified balsa wood as a substrate, onto which hydrophobic lignin nanospheres (m-LNS), derived from waste lignin in the pulp industry, were sprayed to fabricate a Janus membrane with asymmetric wettability, featuring one superhydrophobic surface and one superhydrophilic surface. This all-wood Janus membrane (JW membrane) achieved a separation efficiency of 99.3% and maintained over 98.9% efficiency after 10 cycles, while demonstrating directional and switchable separation for six oil-in-water (O/W) and four water-in-oil (W/O) emulsions.

The physical filtration and emulsion separation mechanisms based on the porous structure of wood which rely on size exclusion and wettability modulation, allow for the physical isolation of droplets or particles, thus enabling the removal of multiphase pollutants such as emulsions. Another example is the selective adsorption of heavy metals via modification with functional groups, whereby coordination groups (i.e., carboxyl, amino, and EDTA) form stable complexes with heavy metal ions, enabling chemical adsorption in a selective and efficient manner [219, 220]. Functionalized wood elements provide enhanced adsorption capacity and selectivity, and exhibit a high degree of pH tolerance. Typically, active sites can be introduced through oxidation, graft polymerization, or the grafting of metal–organic frameworks (MOFs). Functionalization, combined with the existing channel structure of wood, facilitates ion transport and improves adsorption performance. As illustrated in Fig. 17b, Yue et al. [218] developed a wood-based bifunctional membrane modified with MOF–EDTA by leveraging the high surface area of MOF-808 and the multidentate coordination properties of EDTA. This membrane also exploited the innate porous architecture of wood to provide abundant negatively charged sites for the efficient adsorption of cationic dyes such as methylene blue (MB), crystal violet (CV), and rhodamine B (RhB), demonstrating a functional-group-mediated selective adsorption process. The membrane achieved dye removal efficiencies above 94% while maintaining a water flux of 1360 L m^−2^ h^−1^, indicating an excellent compromise between adsorption capacity and permeability.

For anion removal based on charge regulation and ion exchange, cationic groups, such as quaternary ammonium salts or cationic polyelectrolytes, are grafted onto the wood surface to impose a positive surface charge, allowing selective adsorption of common anions present in water, including NO_3_^−^, SO_4_^2−^, and PO_4_^3−^, through electrostatic attraction and ion exchange. Taking advantage of the natural hierarchical porous structure of wood, which augments adsorption kinetics and water flow rate, the charge regulation and ion-exchange approach offers a highly efficient, renewable, and viable material for anionic removal in sustainable water treatment. As documented in Fig. 17c, Ahmed et al. [140] prepared a quaternized wood membrane (QWM) by grafting glycidyltriethylammonium chloride (GTEAC) onto pine wood to introduce quaternary ammonium groups for efficient anion removal. The membrane effectively removed SO_4_^2−^ with a removal efficiency of 98.3%, while maintaining a reasonable water flux (385 to 440 L m^−2^ h^−1^). Moreover, the membrane exhibited good reusability, demonstrating that charge-regulated wood-based materials can be effectively utilized for sustainable water purification.

Wood demonstrates high relevance and multifunctionality in water treatment, enabled by its hierarchical porous structure and tunable surface chemistry. Its 3D architecture supports catalytic degradation via Fenton, Fenton-like, and photo-Fenton systems, where transition metals (e.g., Fe, Cu, Mn) generate reactive oxygen species (ROS) from H_2_O_2_ or PMS, achieving deep oxidation of organic pollutants under light or dark conditions. In parallel, wood's anisotropic channels facilitate high-throughput separation of multiphase pollutants, while functionalization with groups such as EDTA, MOFs, or quaternary ammonium salts enables selective adsorption of heavy metals, dyes, and anions. These features render wood a versatile and regenerable platform for integrated water purification.

#### Solar-Driven Interfacial Water Evaporation

The transpiration mechanism exhibited in natural trees has inspired the use of natural wood as a substrate for solar evaporators, as it possesses good hydrophilicity, low thermal conductivity, and a porous structure facilitating evaporation [108]. Photothermal conversion efficiency can be further enhanced by depositing light-absorbing materials such as plasmonic metals, semiconductor materials, carbonized coatings, polydopamine, or graphite onto the wood surface [64, 221, 222]. Evaporators made from wood substrates functionalized in this manner have been widely used for water purification applications, achieving very good operational parameters for removing heavy metal ions (Cr^3+^, Cu^2+^, Pb^2+^) and organic dyes (methylene blue, methyl orange). However, these pollutants do not vaporize and may remain in the condensed water along with the vapor; therefore, complete separation cannot be achieved solely through water evaporation. This creates a potential risk of secondary contamination, highlighting the need for synergistic designs that integrate bulk photothermal conversion with additional functionalities such as pollutant retention or degradation.

**Fig. 18:**
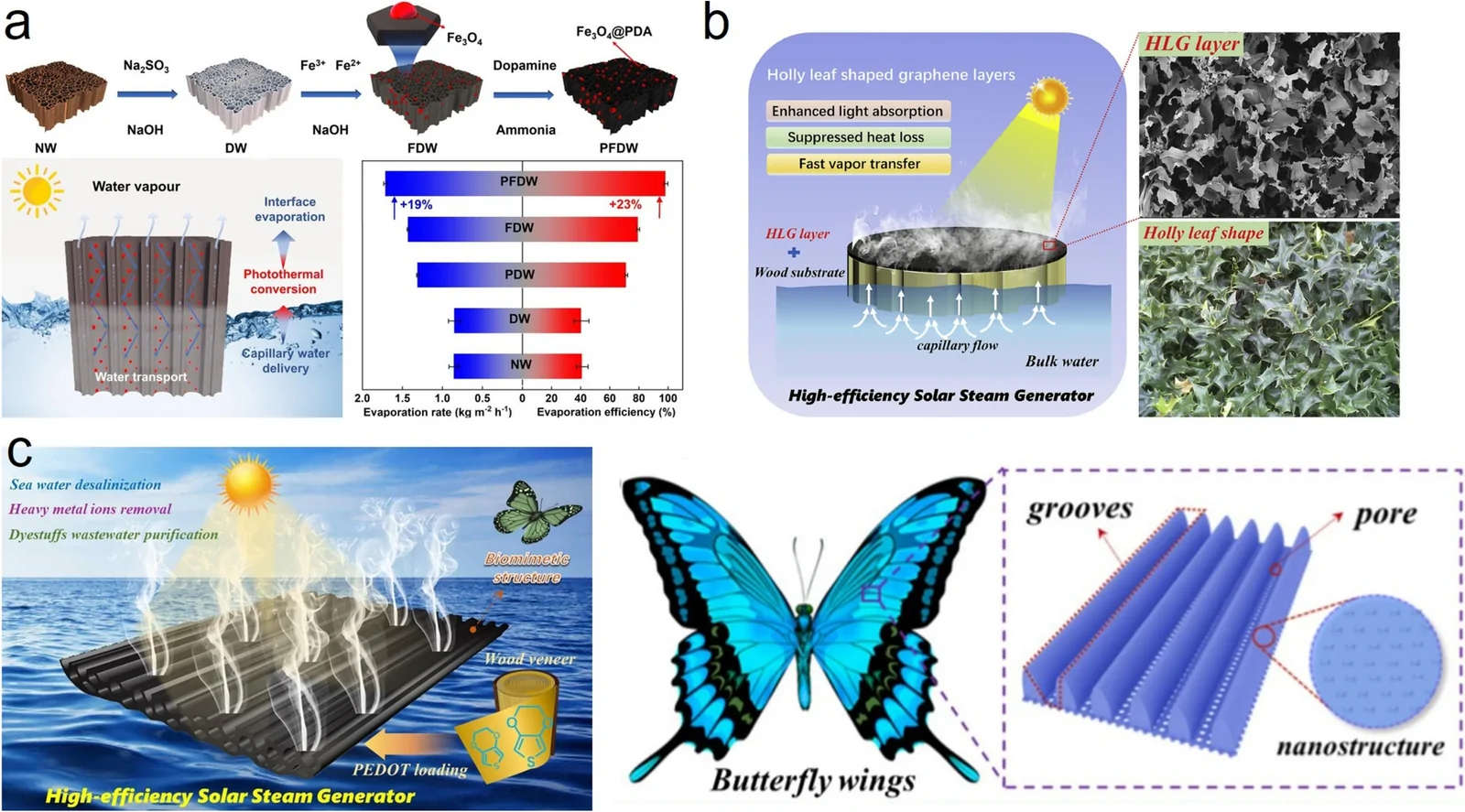
Bioinspired wood-based photothermal materials for solar steam generation. **a** Wood modified with Fe_3_O_4_@PDA via alkali treatment, metal coordination, and dopamine coating enhances photothermal conversion and evaporation rates, reproduced from Ref. [223] with permission from Elsevier, copyright 2023. **b** Holly leaf-like graphene (HLG) layers mimic natural leaf textures, improving light absorption and vapor escape, reproduced from Ref. [224] with permission from Elsevier, copyright 2024. **c** Butterfly wing–inspired MXene structures on wood surfaces boost solar absorption and enable effective salt and heavy metal removal through interfacial evaporation, reproduced from Ref. [221] with permission from Elsevier, copyright 2024

As illustrated in Fig. 18a, Jiang et al. [223] achieved a synergistic effect of photothermal conversion and pollutant adsorption by in situ growth of Fe_3_O_4_ nanoparticles within the channels of delignified wood (DW), followed by coating with polydopamine (PDA). The catechol and amine functional groups in the PDA provided strong adsorption sites for heavy metals and organic pollutants, enabling simultaneous purification of dyes, oils, water, and metal ions. Additionally, Yu et al. [224] demonstrated the development of a hierarchical-like graphene (HLG) layer on the cross-section of lightweight delignified wood (DW, Fig. 18b). Fe^3+^ addition improved the interfacial bonding between graphene oxide and the wood substrate, and the formation of slanted HLG layers was achieved through vacuum impregnation, freeze-drying, and light-assisted reduction. This structure enhanced light absorption, suppressed thermal diffusion, and functioned as a “filtration layer” which substantially suppressed the upward diffusion of dyes and heavy metal ions into condensed water. Their device achieved an evaporation rate of 1.96 kg m^−2^ h^−1^ with a remarkable solar-to-vapor conversion efficiency of 94.2% under 1 kW m^−2^ solar flux. As depicted in Fig. 18c, Li et al. [221] developed an ultrathin wood-based interfacial solar steam generator inspired by the structural morphology of butterfly wings, where poly(3,4-ethylenedioxythiophene) (PEDOT) nanowire and button structures were physically grown onto fir (Pseudotsuga menziesii) wood veneers via in situ vapor-phase polymerization. This was identified as the thinnest and self-floating photothermal material based on wood to date, with a thickness of only 0.6 mm. The effective structural design helped prevent direct contaminant transfer with evaporating water vapor, thereby reducing the risk of secondary pollution. Cui et al. [225] also developed a bifunctional wood membrane incorporating MoS₂/covalent organic framework (COF) heterojunctions, utilizing the inherent porosity of wood, the thin structure of π-conjugated COFs, and the photochemical properties of MoS_2_. This architecture provided efficient solar-driven water evaporation (2.17 kg m^−2^ h^−1^) and simultaneously achieved organic dye waste degradation (removal efficiency > 99%). Combining photocatalysis with water evaporation limits the deposition of non-volatile contaminants on the evaporator surface, thereby minimizing the risk of secondary pollution in condensed water.

**Fig. 19:**
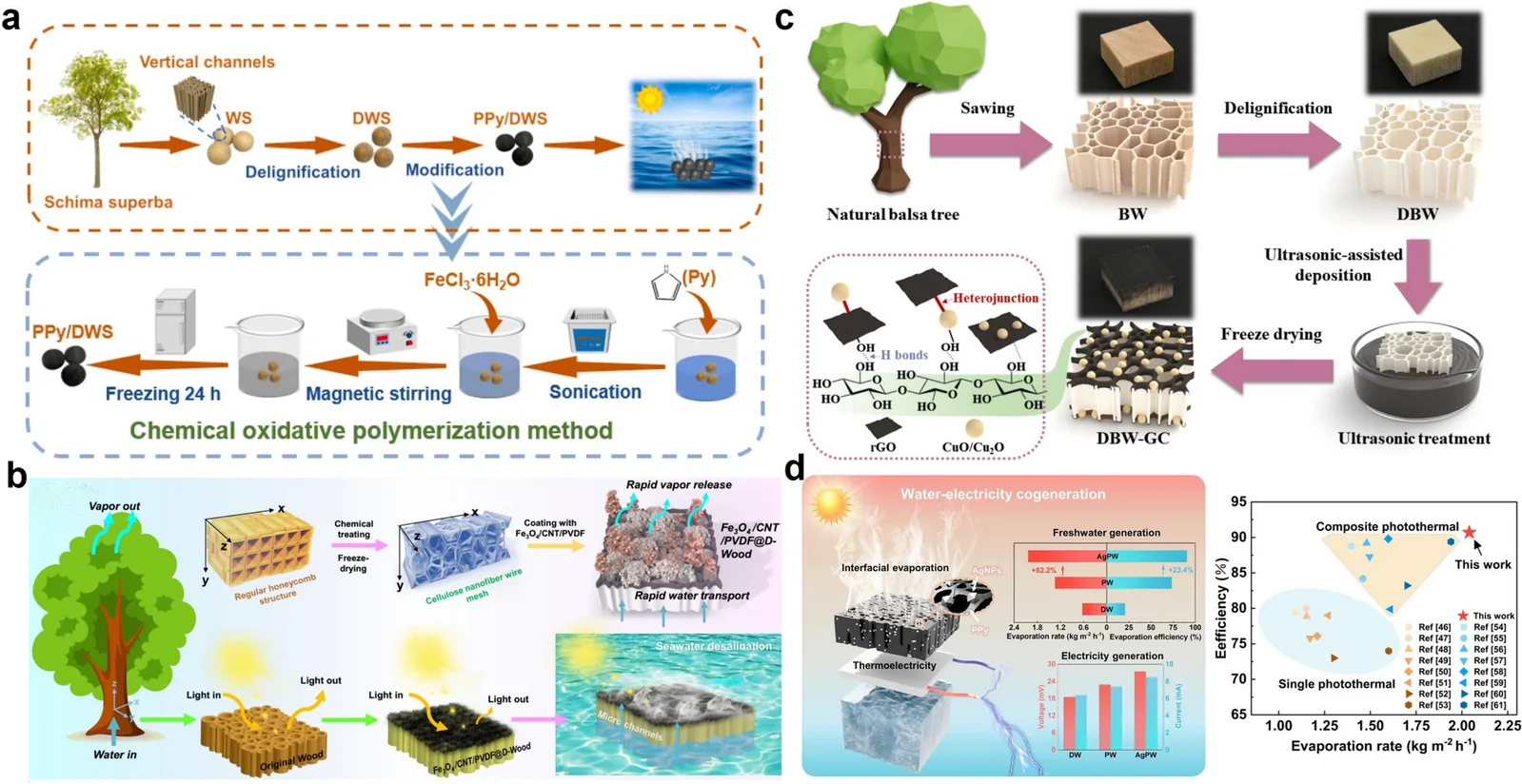
Functionalized wood-based evaporators for solar-powered water purification. **a** Preparation of PPy-modified delignified wood by chemical oxidative polymerization, which enhances solar absorption and interfacial heating and generates steam, reproduced from Ref. [108] with permission from Elsevier, copyright 2024. **b** Photothermal wood evaporator constructed using Fe_3_O_4_ and CNTs combined with carbonized wood for localized heating and high-efficiency seawater desalination, reproduced from Ref. [107] with permission from Elsevier, copyright 2025. **c** Composite evaporator constructed with CuCrO_2_ and graphene oxide deposited on delignified balsa wood (DBW), which was freeze-dried, allows for rapid water transport and light-to-heat conversion, reproduced from Ref. [64] with permission from Elsevier, copyright 2024. **d** Composite architectures composed of wood frames exhibit superior water evaporation rates and photothermal conversion efficiencies under one-sun illumination compared to single-material counterparts, reproduced from Ref. [141] with permission from Elsevier, copyright 2024

With respect to freshwater harvesting, wood-based solar evaporators have gained worldwide attention for seawater desalination, where the research focus has shifted significantly from the traditional goals of pollutant adsorption and catalytic degradation in wastewater treatment [141]. Seawater desalination aims to provide continuous and stable freshwater production while including high salinity tolerance, floating ability, self-cleaning, and resistance to salt crystallization through sustained evaporation [107]. Therefore, critical objectives include maintaining overall structural stability, ensuring long-term tolerance against salt, and achieving sustainable evaporation efficiency [226]. To meet these demands, researchers primarily regulate the porous architecture of wood, enhance buoyancy and mechanical support in design, incorporate anti-salt fouling interfaces, and develop self-cleaning or self-rotating functions. These strategies minimize salt accumulation and crystallization at the evaporation interface, enabling the system to operate stably and sustain evaporation even under prolonged or high-salinity conditions. For example, as shown in Fig. 19a, Wang et al. [108] designed and constructed a self-rotating, floating wood-based solar evaporator composed of delignified wood spheres (DWS) coated with PPy to enhance hydrophilicity and light absorption. The asymmetric design enabled autonomous rotation, preventing salt accumulation and maintaining evaporation rates of 2.43 kg m^−2^ h^−1^ in 3.5 wt% saline and 1.52 kg m^−2^ h^−1^ in 20 wt% saline, respectively. As illustrated in Fig. 19b, Zhang et al. [107] fabricated a multifunctional evaporative membrane by brush-coating delignified wood with a Fe_3_O_4_/CNT-PVDF composite. The membrane exhibited asymmetric wettability, magnetic positioning, and wind resistance, achieving an evaporation rate of 1.92 kg m^−2^ h^−1^ and 129.08% photothermal efficiency, while maintaining stability under salinity, oil fouling, and 6.6 m/s wind. As presented in Fig. 19c, Wo et al. [64] constructed an integrated evaporator (DBW-GC) using partially delignified wood loaded with reduced graphene oxide (rGO) and CuO/Cu_2_O nanocomposites. It maintained an evaporation rate of 1.79 kg m^−2^ h^−1^ in 20 wt% saline through a synergistic mechanism of salt diffusion, capillary transport, and self-cleaning during dark cycles, ensuring long-term operational stability. As observed in Fig. 19d, Lu et al. [141] prepared a Ag/PPy composite evaporator by in situ polymerization and AgNP deposition onto delignified wood. By combining dual photothermal mechanisms with efficient water transport, the device achieved an evaporation rate of 2.04 kg m^−2^ h^−1^ at 90.7% efficiency, and generated stable electricity output (27.5 mV), while exhibiting strong salt resistance, antibacterial activity, and environmental adaptability.

Wood-based materials have emerged as multifunctional platforms for water purification owing to their hierarchical porous architecture, tunable surface chemistry, and inherent sustainability, enabling integration of catalytic degradation, adsorption/filtration, and solar-driven interfacial evaporation within a single material framework. In catalytic systems, wood serves as a robust scaffold for transition-metal-based Fenton, photo-Fenton, and Fenton-like catalysts (e.g., Fe, Cu, Mn), facilitating the generation of reactive oxygen species (·OH, SO_4_^−^, ·O_2_^−1^, O_2_) from H_2_O_2_ or PMS under light or dark conditions, achieving rapid and deep oxidation of recalcitrant organic pollutants with high efficiency (e.g., > 97% removal of tetracycline within 60 min, complete ciprofloxacin degradation in 4 min). In adsorption–filtration interfaces, delignification and surface functionalization with hydrophobic coatings, MOFs–EDTA, or quaternary ammonium groups endow wood membranes with directional liquid transport, high separation efficiency for oil–water emulsions (> 99%), and selective removal of dyes, heavy metals, and anions, while maintaining high flux and reusability [108]. In solar-driven evaporation, photothermal functionalization with Fe_3_O_4_, graphene, MXene, PEDOT, or polydopamine enables high evaporation rates (up to 2.43 kg m^−2^ h^−1^) and solar-to-vapor conversion efficiencies (> 94%), combined with pollutant adsorption/degradation and anti-salt-fouling capabilities for stable long-term seawater desalination [202]. Collectively, these studies reveal that precise structural regulation, catalytic interface engineering, and multifunctional surface modification can transform wood into an integrated, high-performance, and sustainable water treatment platform capable of addressing diverse pollutant classes and operational scenarios [222].

### Energy Harvesting Using Functionalized Wood

#### Photothermal and Photovoltaic Energy Conversion Using Functionalized Wood

Interfacial solar steam generation (ISSG) is a proficient solar energy conversion technology that offers a cost-effective solution that alternative methods struggle to achieve in addressing the energy crisis and freshwater scarcity. The inherent porous structure of wood and its capacity for water transport provide essential features suitable for enhancing water evaporation and photothermal conversion in wood-based ISSG systems [227–231]. Recently, wood-based evaporators have been extensively engineered to maximize energy utilization, benefiting from the development of carbonized wood evaporators, MXene composites, and metal nanoparticles (e.g., Ag, Fe_3_O_4_, MgFe_2_O_4_), while 3D printing techniques have facilitated advanced structural designs. In addition, ISSG has been explored for integrated power generation, including thermoelectric conversion (Bi_2_Te_3_, Sb_2_Te_3_), steam-powered micro-turbines, and hydrovoltaic energy harvesting via ion gradient-initiated processes [232]. However, challenges remain to address photothermal conversion inefficiencies leading to energy losses, and to improve thermal management by optimizing nanoporous composites and thermoelectric recovery modules, thereby enhancing overall ISSG performance [233].

**Fig. 20:**
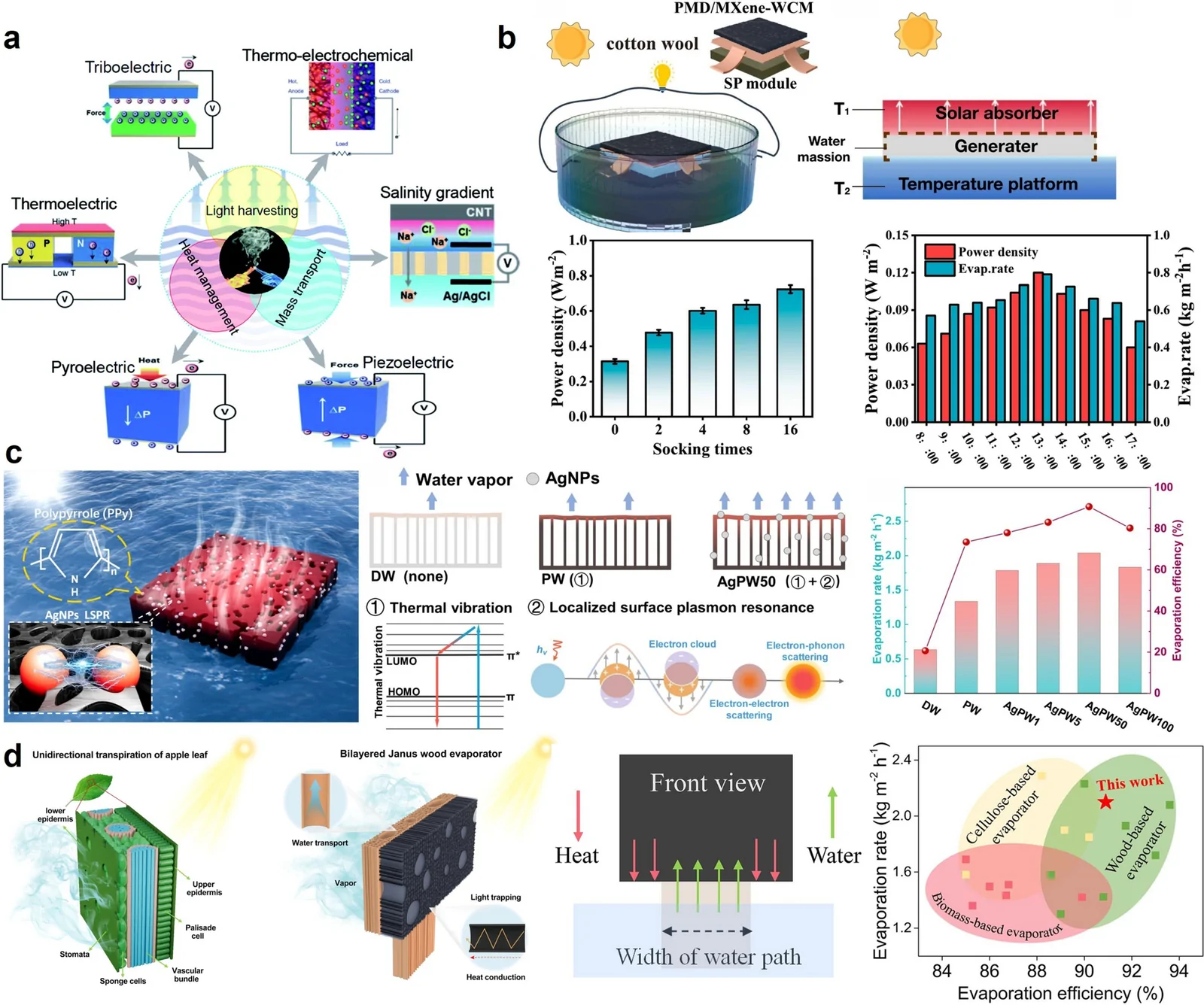
Wood-based systems for solar energy harvesting and electricity generation. **a** Schematic of integrated energy harvesting via thermoelectric, triboelectric, pyroelectric, piezoelectric, and salinity gradient effects enabled by wood-derived platforms, reproduced from Ref. [234] with permission from Royal Society of Chemistry, copyright 2024. **b** PMD/MXene-coated wood combined with thermoelectric modules achieves higher output through optimized soaking, reproduced from Ref. [101] with permission from Elsevier, copyright 2024. **c** AgNP-functionalized wood evaporators enhance photothermal conversion via localized surface plasmon resonance and thermal vibration, reproduced from Ref. [141] with permission from Elsevier, copyright 2025. **d** Bilayer Janus wood evaporator engineered with narrowed water channels reduces heat loss and achieves over 90% evaporation efficiency under 1 sun, reproduced from Ref. [235] with permission from Elsevier, copyright 2024

Photothermal conversion is the process by which energy from incident light is transformed into thermal energy within a material [231]. Photothermal materials play a critical role in this process, efficiently converting absorbed light into heat through photoexcitation. Ideally, such materials should exhibit broad-spectrum absorption across the solar spectrum to maximize thermal energy output [230]. Strong light absorption capabilities have been demonstrated by various materials, including carbon-based nanomaterials, plasmonic nanomaterials, and inorganic semiconductors. Generally, the main mechanisms behind their photoexcitation include nonradiative relaxation leading to molecular thermal vibration, plasmonic heating, and the generation and relaxation of electron–hole pairs. Six representative energy conversion pathways are illustrated in Fig. 20a [234]. Beyond simple heat generation, the thermal energy absorbed by photothermal materials can also be further converted into electricity through mechanisms within solar-driven steam generation systems, including triboelectric, piezoelectric, thermoelectric, thermoelectrochemical, and salinity gradient effects. In these systems, the triboelectric effect captures energy in the form of charges generated by the condensation of vapor or the movement of liquid droplets on surfaces. The piezoelectric effect produces an electric potential from material deformation caused by steam flow or droplet impacts. The thermoelectric effect enables the direct conversion of thermal gradients into electricity through carrier migration, typically via the Seebeck effect. Meanwhile, the thermoelectrochemical effect utilizes temperature gradients to drive redox reactions, enabling continuous power output. Lastly, the salinity gradient effect exploits localized differences in salt concentrations induced by evaporation to generate electric potentials across electrodes or ion-selective membranes. These mechanisms can function independently or synergistically, significantly enhancing the overall energy efficiency of solar steam generation systems that simultaneously produce freshwater and electrical energy.

For solar-driven evaporation systems, thermoelectric power generation plays a dominant role. A thermoelectric system exploits the Seebeck effect, where electron flow is driven by a temperature gradient, converting thermal energy directly into electrical energy. After absorbing solar radiation, thermoelectric materials generate thermal gradients by heating the interfacial water layer while maintaining a cooler bulk water temperature, supporting simultaneous vapor generation and electricity harvesting. The thermoelectric module is positioned across this gradient to promote electron migration and generate electric output. As can be seen in Fig. 20b,Wu et al. [101] proposed a flexible wood-based composite material (PMD/MXene-WCM), where MXene nanosheets were incorporated into the porous wood framework to enhance photothermal conversion efficiency. A thermoelectric generator (TEG) module was also attached to the bottom surface to recover waste heat through the Seebeck effect by utilizing the temperature difference between the evaporation interface and the bulk water. Under 1 kW m^−2^ solar irradiation, the PMD/MXene-WCM achieved an evaporation rate of 1.59 kg m^−2^ h^−1^, a photothermal conversion efficiency of 95.24%, and a thermoelectric power density of 0.71 W m^−2^, establishing a high-performance water-electricity co-generation system. As recorded in Fig. 20c, Lu et al. [141] developed a PPy/AgNPs-functionalized wood-based evaporator (AgPW) and utilized residual heat to drive a thermoelectric generator (TEG) for stable power generation. The AgPW was fabricated via in situ polymerization, enhancing light absorption, while the delignified wood’s vertical porous structure optimized water transport, achieving a synergistic thermal-water management system. Under 1 kW m^−2^ solar irradiation, the optimized AgPW50 exhibited an evaporation rate of 2.04 kg m^−2^ h^−1^ and a solar-to-vapor conversion efficiency of 90.7%, while the TEG module generated a maximum power density of 0.71 W m^−2^, realizing efficient water-electricity cogeneration. As presented in Fig. 20d, Dai et al. [235] inspired by the unidirectional transpiration mechanism of apple leaves, proposed a bilayer Janus wood evaporator (BJWE) that optimizes water-thermal transport by decoupling light absorption and water evaporation interfaces. The BJWE consists of a longitudinal wood layer (W-layer) for efficient water transport and a carbonized transverse wood layer (P-layer) to enhance photothermal conversion, with polydimethylsiloxane (PDMS) modification to improve thermal management. Under 1 kW m^−2^ solar irradiation, BJWE achieved an evaporation rate of 2.12 kg m^−2^ h^−1^and a solar-to-vapor conversion efficiency of 92.3%, outperforming conventional wood-based evaporators. Moreover, it demonstrated stable operation for 8 h in 3.5 wt% saline water, effectively purifying organic pollutants and oil–water emulsions.

Wood-based photothermal and photovoltaic energy conversion systems leverage their inherent multiscale porous architecture, efficient water transport capability, and functionalizable surfaces to achieve multimodal energy harvesting and conversion (light–heat–electricity) in interfacial solar steam generation (ISSG) [236]. Studies have demonstrated that carbonization, MXene integration, metal nanoparticle modification (e.g., Ag, Fe_3_O_4_, MgFe_2_O_4_), and biomimetic structural designs (such as bilayer Janus wood structures) can markedly enhance light absorption efficiency, interfacial thermal management, and evaporation rates (up to 2.12 kg m^−2^ h^−1^), attaining solar-to-vapor conversion efficiencies exceeding 90%. Moreover, coupling the photothermal system with thermoelectric (TEG) modules enables Seebeck effect-based power generation by exploiting the temperature gradient between the interfacial and bulk water, achieving a thermoelectric power density of up to 0.71 W m^−2^ for simultaneous water and electricity production. In addition, integrating plasmonic resonance, thermal conductivity regulation, and water/heat separation interfacial designs effectively reduces energy losses and extends operational stability in seawater and complex water treatment processes. These findings indicate that wood-based ISSG, through precise structural engineering, functional material hybridization, and multisource energy harvesting strategies, offers a high-efficiency, sustainable solar energy platform with light–heat–electricity multifunctional conversion capability, providing a promising pathway for solar-driven freshwater production and distributed energy supply [227, 228, 237].

#### Hydropower Generation

**Fig. 21:**
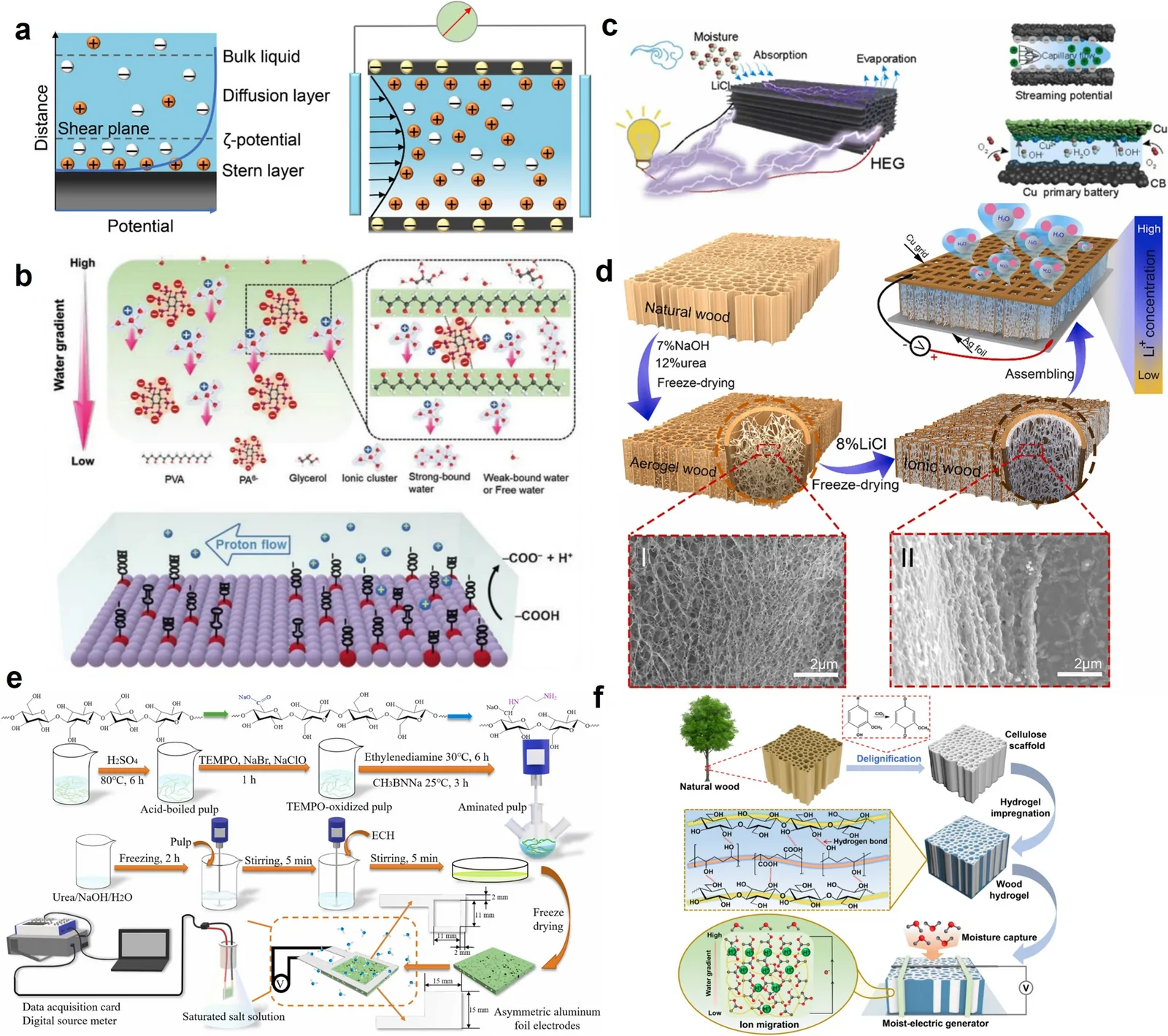
Wood-based materials for energy conversion, electrochemical interfaces, and solar desalination. **a** Electric double layer structure at electrode–electrolyte interfaces, highlighting ion distribution critical for capacitive enhancement in wood-derived electrodes, reproduced from Ref. [238] with permission from Wiley–VCH, copyright 2016. **b** Ion transport in hierarchical porous wood channels, with surface charge modulation enabling selective ion movement and enhanced electrochemical interaction, reproduced from Ref. [239, 243] with permission from Springer Nature, copyright 2018. **c** Solar steam generation device integrating holey graphene (HEG) and polyelectrolyte hydrogel with wood for improved light absorption, vapor transport, and desalination efficiency, reproduced from Ref. [246] with permission from American Chemical Society, copyright 2024. **d** Ionic thermoelectric material fabricated by freeze-drying delignified wood and assembling PEDOT:PSS onto aligned cellulose frameworks for selective ion transport, reproduced from Ref. [247] with permission from Wiley–VCH, copyright 2023. **e** Photoelectrocatalytic system combining wood-derived carbon and TiO_2_ nanosheets for efficient solar-driven pollutant degradation and energy harvesting, reproduced from Ref. [248] with permission from Elsevier, copyright 2022. **f** Wood–polymer–MXene composite designed for ion sieving and multifunctional solar energy conversion, utilizing the anisotropic porous structure and modifiable surfaces of wood, reproduced from Ref. [248] with permission from Elsevier, copyright 2022

As the global energy crisis and environmental pollution intensify, hydrovoltaic generation has emerged as a sustainable solution for harvesting electricity from water sources, including bulk water, flowing water, and humidity, without relying on external light, heat, or mechanical input [238]. Wood, with its hierarchical porous structure and hydrophilic cellulose nanofibrils, enhances ion transport and charge migration, optimizing hydrovoltaic energy conversion. As indicated in Fig. 21a, streaming potential, an electrokinetic phenomenon first described by Quincke in 1859, and ion gradient diffusion serve as key mechanisms. Streaming potential arises from charge migration at the water–solid interface due to the formation of an electric double layer (EDL), while ion gradient diffusion is driven by concentration gradients of dissociated ions [239–241]. As detailed in Fig. 21b, asymmetric humidity exposure or functional group gradients can further enhance ion diffusion and energy harvesting efficiency. The natural capillary action and reversible moisture adsorption of wood ensure stable power generation under varying humidity conditions, making it a promising, biodegradable, and cost-effective material for next-generation hydrovoltaic applications [242–245].

However, natural wood has many limitations in meeting the conditions of use, mainly in terms of low electrical conductivity, limited environmental adaptability, low power output, and long-term stability issues. Therefore, wood-based MENGs require modification through structural optimization, surface functionalization, or nanomaterial integration to enhance their electrical performance, durability, and energy conversion efficiency [149]. As reflected in Fig. 21c, in order to solve the problem of power output, Zhang et al. [249] modified the surface of wood by coating the surface of wood sponge with carbon black ink and using lithium chloride (LiCl) solution as a hygroscopic agent. After this treatment, the power output of the generator reached 216 μW. In addition, the performance of the generator was further enhanced by adding copper electrodes and constructing a primary battery system. Zhang et al. [250] immersed natural wood in Chinese ink and coated the surface of its microchannels with charged carbon nanoparticles, which could generate an open-circuit voltage of about 250 mV under ambient conditions and work stably for more than 72 h. In addition, an electronic calculator can be driven by connecting six WMEG devices in series. As documented in Fig. 21d, Li et al. [246] partially dissolved the wood cell walls using NaOH/urea treatment, followed by freeze-drying to reconstruct a nanostructured fiber network, thereby enhancing water transport capability. Subsequently, the wood was immersed in an 8 wt% LiCl solution, leading to the formation of spiderweb-like ionic bridges within the microchannels, which facilitated ion migration efficiency.

Apart from wood-based materials for moisture-electric generation, cellulose derived from wood can also be utilized for this purpose, leveraging its superior hydrophilicity and ionic conductivity. The ordered porous structure constructed by nanocellulose (CNF/CNC) effectively enhances water absorption and ion transport efficiency, thereby improving the performance of moisture-driven power generation. As indicated in Fig. 21e, Huang et al. [247] developed a moist-electric generator (MEG) based on oxidized and aminated regenerated cellulose (ORC/ARC), which exploits its high hydrophilicity and functional groups to achieve moisture-driven energy conversion. ORC and ARC were prepared via TEMPO oxidation and ethylenediamine amination, followed by cross-linking and freeze-drying to fabricate a porous aerogel, thereby enhancing the MEG’s performance. As illustrated in Fig. 21f, Zhang et al. [248] employed a delignification (DL) treatment on balsa wood, exposing more hydrophilic cellulose fibers. Furthermore, they incorporated a polyvinyl alcohol (PVA)/polyacrylic acid (PAA) ionic hydrogel to enhance ionic conductivity and moisture absorption capacity, thereby improving the overall efficiency of moisture-electric conversion.

**Fig. 22:**
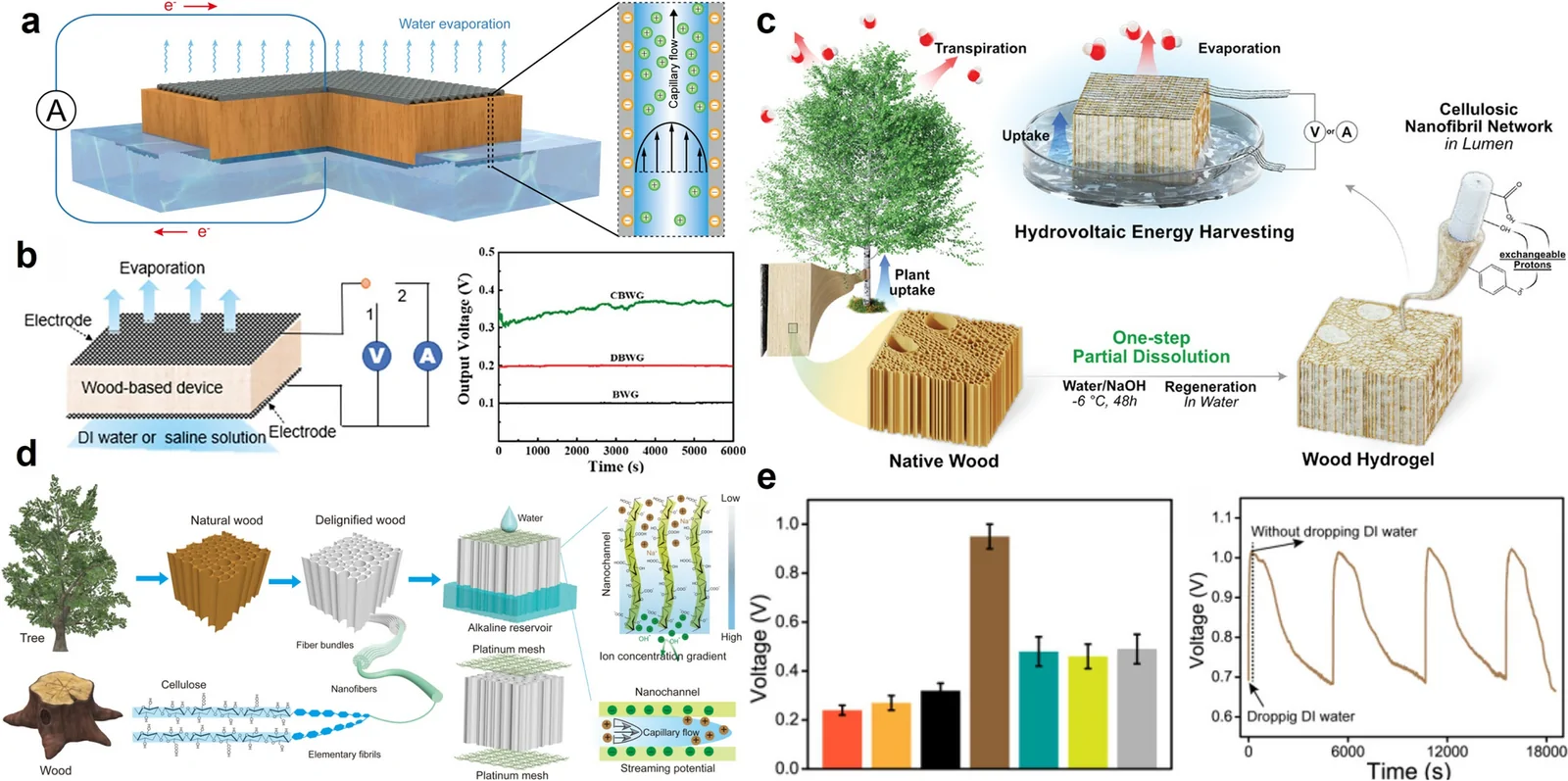
Wood-based strategies for hydrovoltaic and ionic thermoelectric energy harvesting. **a** Evaporation-driven electricity generation using wood channels for capillary water transport and ion diffusion, reproduced from Ref. [251] with permission from American Chemical Society, copyright 2020. **b** Stable voltage output from a wood-based device tested in different aqueous solutions under ion gradient conditions, reproduced from Ref. [252] with permission from American Chemical Society, copyright 2020. **c** Hydrovoltaic energy harvesting achieved by converting natural wood into a cellulose nanofiber hydrogel via one-step partial dissolution, reproduced from Ref. [244] with permission from Wiley–VCH, copyright 2022. **d** Fabrication of a delignified wood nanocomposite embedded with polyanionic polymers and hydrogels to construct an ionic thermoelectric generator, reproduced from Ref. [103] with permission from Elsevier, copyright 2022. **e** Comparison of output voltages from various wood-based ionic thermoelectric materials and voltage response under moisture variation, reproduced from Ref. [103] with permission from Elsevier, copyright 2022

As revealed in Fig. 22a, the microchannel structure of natural wood enables ion migration driven by water evaporation, facilitating energy harvesting. The wood’s microchannels absorb water through capillary action and allow evaporation at the top, establishing a continuous water flow process [253]. Due to the hydroxyl (-OH) groups on the wood cell walls, which dissociate to form negatively charged surfaces in aqueous solutions, cations (e.g., H^+^, Na^+^) migrate along the flow direction under the electric double-layer effect, while anions are retained within the channels, leading to charge separation between the two ends of the wood, generating a streaming potential. This electrokinetic effect is sustained during continuous water evaporation, driving steady DC current output [103, 254].

Studies have shown that microchannel size and alignment significantly impact power generation efficiency, with optimal diameters between 5 and 40 μm. Longitudinally aligned channels facilitate water transport and ion migration, enhancing energy output. Furthermore, citric acid modification improved the zeta potential of the wood surface, increasing the hydrophilicity and charge separation, thereby enhancing electrical output. A single wood-based nanogenerator achieved an open-circuit voltage (V_oc_) of 300 mV and a short-circuit current (I_sc_) of 10 μA [251]. As depicted in Fig. 22b, Piao et al. [252] employed balsa wood as a porous substrate and conducted in-situ polymerization of PPy within the wood microchannels to enhance water transport and ion migration capabilities. Optimizing wood porosity and evaporation interfaces further improves energy conversion efficiency. Lin et al. [104] developed an all-wood evaporation-induced electricity generator (WEIG), utilizing delignified wood (DBW) and delignified-hemicellulose-removed wood (CBW) to optimize charge transport and power output. The CBW-based WEIG (CBWG) achieved a V_oc_ of 0.4 V in deionized (DI) water, significantly surpassing DBWG (0.2 V) and untreated wood (BWG, 0.1 V). Moreover, both I_s_c and maximum power output (P) were substantially enhanced with increased modification levels. As demonstrated in Fig. 22c, Garemark et al. [244] treated wood with 8 wt% NaOH solution at –6 C for 48 h, increasing its specific surface area from 1–2 to 210 m^2^ g^−1^. The functionalized wood achieved a V_oc_ of 140 mV in DI water, a tenfold increase compared to untreated wood, and further improved to 550 mV in pH 13.4 alkaline solution. Additionally, carboxyl (-COOH) and phenolic hydroxyl (-OH) groups on the wood surface underwent charge dissociation at different pH levels, shifting the zeta potential from -33 to -48 mV, enhancing charge separation and power generation efficiency. As presented in Fig. 22d, e, Zhang et al. [103] developed a wastewater-driven ionic gradient energy harvesting system. In deionized water, the system achieved a V_oc_ of 0.25 V, which increased to 1.1 V in 4 M NaOH solution, with a maximum short-circuit current (I_sc_) of 320 μA and a maximum power density of 6.75 μW cm^−2^. By regulating water evaporation rates and ion concentration gradients, the generator demonstrated long-term stability in highly alkaline wastewater environments (e.g., black liquor from pulp production), providing power to electronic devices.

Wood-based hydrovoltaic energy harvesting systems exploit the hierarchical porous structure and hydrophilic cellulose nanofibrils of wood to facilitate efficient ion transport and charge separation via streaming potential and ion-gradient diffusion [255], enabling sustainable electricity generation from bulk water, flowing water, and ambient moisture without reliance on light, heat, or mechanical input. Through structural optimization (e.g., delignification, hemicellulose removal, microchannel alignment), surface functionalization (e.g., citric acid modification, charged carbon nanoparticle coating, ionic hydrogels), and nanomaterial integration (e.g., carbon black, PPy, MXene), these systems achieve significant performance enhancements, with open-circuit voltages up to 1.1 V, short-circuit currents of 320 μA, and maximum power densities of 6.75 μW cm^−2^ [103]. Functionalized designs not only improve hydrophilicity, zeta potential, and evaporation-driven ion migration efficiency but also deliver long-term operational stability in challenging environments such as high-salinity or alkaline wastewater (e.g., black liquor), enabling direct powering of low-energy electronics. These advances position wood-based hydrovoltaic platforms as biodegradable, low-cost, and high-efficiency candidates for next-generation distributed energy systems [247].

#### Wood-Based Triboelectric Nanogenerators

Triboelectric nanogenerators (TENG) have become an important technological choice for implantable and wearable electronic devices due to their self-powered capability, efficient energy conversion and stable power output [256, 257, 278–280]. Since Wang et al. [258] proposed TENG based on contact charge effect and electrostatic induction effect in 2012, the technology has been widely used in the fields of wearable devices, sports medicine, and smart home by virtue of its high efficiency of low-frequency energy capture, abundant material choices, flexible structural design, and low cost. In recent years, TENG, as an efficient mechanical-to-electrical energy conversion technology, combines the coupling effect of friction charging and electrostatic induction to realize a low-cost and simple structure of self-powered energy system, which can be integrated with energy storage devices to provide sustainable power supply for microelectronic devices [259]. In addition, TENG, based on Maxwell's principle of displacement current, can efficiently harvest mechanical energy and operate independently without an external power source, increasing the spatial flexibility of the system [260]. Its applications have expanded to include self-powered sensors such as pressure, haptic and motion sensing, and it has shown great potential in the fields of sensor networks, artificial intelligence and the Internet of Things (IoT) [261]. However, conventional TENGs are mostly made of metal or polymer materials that are difficult to recycle and degrade, and their long-term use may pose environmental pollution problems [262]. Therefore, the use of wood as a friction electric layer explores the sustainable application of TENG in the field of green energy with its natural biodegradable and non-polluting properties. Wood is not only a common and widely used decorative material, but also plays an important role in interior design, providing TENG with both functional and eco-friendly material options [263–266, 281–284].

**Fig. 23:**
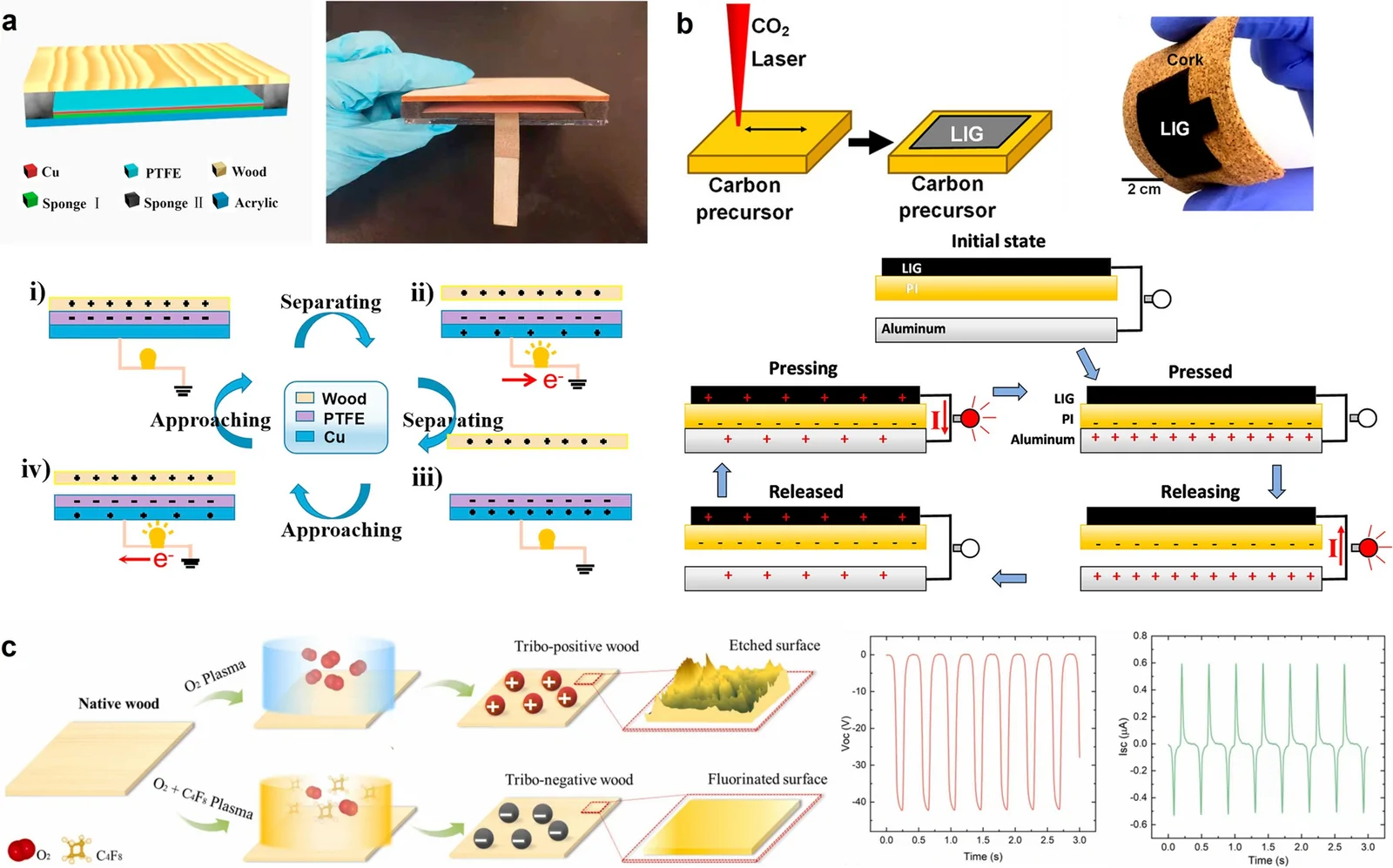
Structural designs, fabrication processes, and surface modification strategies of wood-based triboelectric nanogenerators (W-TENG). **a** Single-electrode W-TENG using New Zealand Pine/PTFE triboelectric layers and Cu electrode, showing device structure and contact–separation working principle. Reproduced from Ref. [265] with permission from Elsevier, copyright 2020. **b** LIG-based TENGs prepared by laser irradiation of cork or lignin/PLLA composites, enhancing conductivity, sustainability, and charge storage capacity. Reproduced from Ref. [267] with permission from the American Chemical Society, copyright 2019. **c** Surface modification of wood via plasma treatment and chemical functionalization to tune triboelectric properties and improve output performance. Adapted from Ref. [268] with permission from The Author(s), copyright 2022, under Creative Commons CC BY license

The wood-based triboelectric nanogenerator (W-TENG) is an environmentally friendly, renewable, and easily manufacturable self-powered device. As shown in Fig. 23a, Hao et al. [265] developed a single-electrode mode W-TENG utilizing New Zealand Pine and polytetrafluoroethylene (PTFE) as the triboelectric layers, with copper (Cu) as the electrode. The device, with dimensions of 8 cm × 8 cm, achieves an open-circuit voltage of 220 ± 20 V and a short-circuit current of 5.8 ± 0.5 μA at a frequency of 2 Hz, with a maximum power density of 158.2 mW m^−2^, capable of driving 42 commercial LEDs. Based on W-TENG, the introduction of laser-induced graphene (LIG) further improves the performance of TENG, while enhancing its green and sustainability. As a metal-free, highly conductive electrode material with porous structure, LIG can not only replace the traditional metal electrodes and reduce resource consumption and environmental pollution, but also reduce the fabrication cost, as well as enhance the flexibility and degradability of the device. As shown in Fig. 23b, Stanford et al. [267] carbonized the surface of cork by CO_2_ laser irradiation to directly generate LIG layers with high conductivity and porous structure. The resulting LIG/cork composite has a LIG layer thickness of ~ 300 μm, a surface resistance of ~ 115 Ω sq^−1^, and exhibits a typical graphene 2D peak (~ 2690 cm^−1^) in Raman spectroscopy, which demonstrates its successful conversion to LIG. Based on this, the researchers constructed a single-electrode model TENG (STENG) in which LIG serves as the conducting layer while retaining the natural porous properties of cork to enhance charge storage capacity. The TENG achieves an open-circuit voltage of 35 to -105 V and a maximum power density of about 0.76 W m^−2^ at 2 N force. Furthermore, Funayama et al. [269] fabricated conductive graphitic carbon structures on degradable lignin/poly(lactic acid) (PLLA) composite films by femtosecond laser pulse irradiation. The prepared TENG exhibited a power density of 1.98 mW m^−2^ at a load resistance of 200 MΩ at a frequency of 1 Hz with 1 N contact pressure. The TENG is also capable of harvesting electrical energy from natural resources, such as water droplet contact and plant leaf touch, demonstrating its potential for application in environmental energy harvesting. Surface modification In addition to laser-induced graphene, as shown in Fig. 23c, Sun et al. [268] used oxygen (O_2_) plasma and C_4_F_8_ + O_2_ hybrid plasma to treat wood to modulate the friction electrical properties of wood. The maximum output voltage was up to 227 V with a current of 4.8 μA. Liao et al. [270] used 3-aminopropyltriethoxysilane (APTES) and fluorinated silane (PFDTMS) to chemically modify wood to enhance its hydrophobicity and optimize its friction charge storage capacity.

**Fig. 24:**
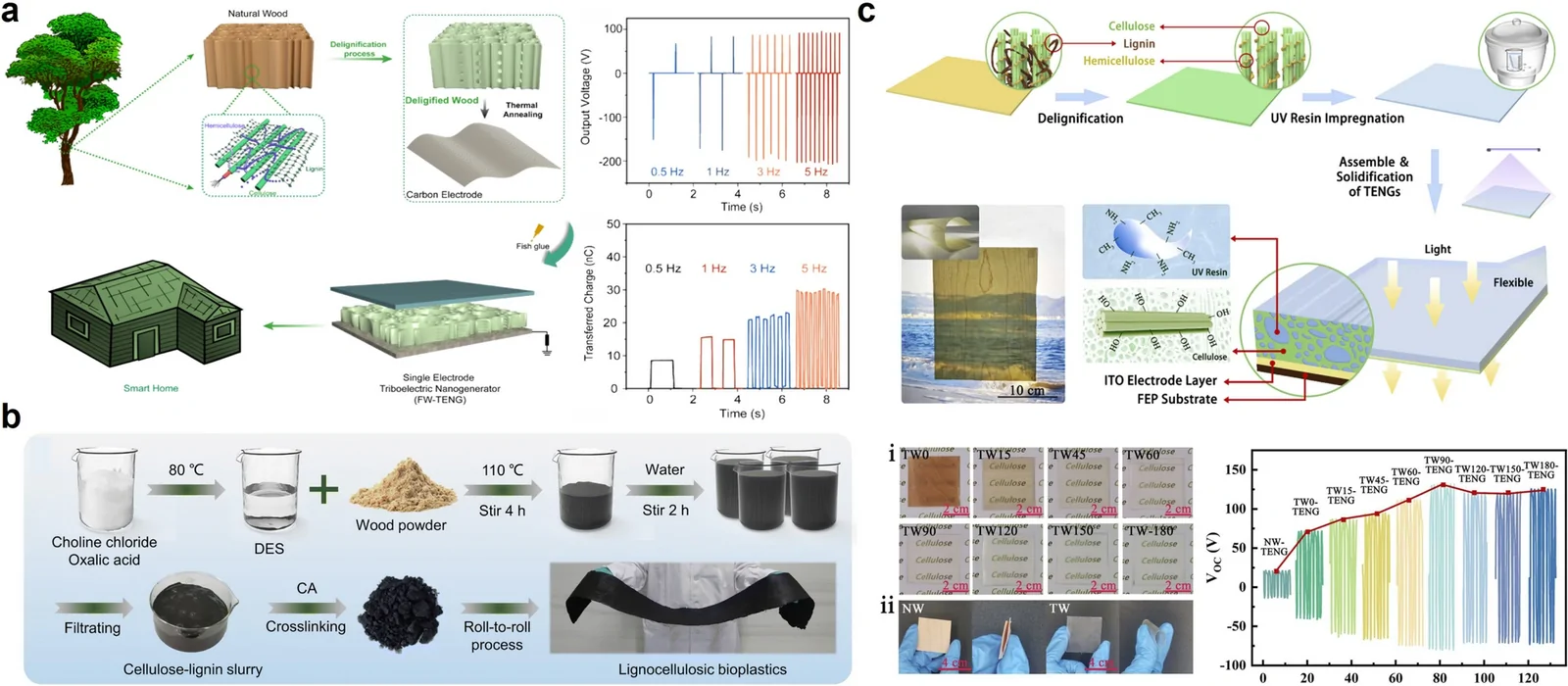
Structural modification strategies for enhancing the performance of wood-based triboelectric nanogenerators (W-TENG). **a** Delignification and carbonization of eucalyptus wood to increase porosity and conductivity, significantly improving voltage output and charge transfer capability of W-TENG. Reproduced from Ref. [257] with permission from Springer Nature, copyright 2024. **b** Deep eutectic solvent (DES)-based in-situ lignin regeneration and cross-linking modification to form degradable lignocellulosic composites with enhanced mechanical strength and triboelectric performance. Reproduced from Ref. [271] with permission from Royal Society of Chemistry, copyright 2023. **c** Preparation of transparent wood-based TENGs via delignification and UV-curable resin impregnation, achieving high transparency and improved triboelectric output. Reproduced from Ref. [272] with permission from Elsevier, copyright 2024

The application of wood as TENG is not only limited to surface modification, but further optimizing its performance through structural modification has become an important direction of current research. By modulating the cell wall structure, delignification can effectively increase the porosity of wood and improve its charge storage capacity, thus improving the friction electrical characteristics. As shown in Fig. 24a, Ma et al. [257]modified eucalyptus wood by delignification through NaOH/Na_2_SO_3_ treatment for 12 h and heat treatment at 100 °C for 5 h to form a highly porous structure to enhance the charge storage and transport capacity. Subsequently, a highly conductive wood-based carbon electrode was prepared by carbonization at 800 °C, and a single-electrode TENG was constructed, which showed that the open-circuit voltage of the FW-TENG reached 208 V at 5 Hz, much higher than that of the unlignified eucalyptus TENG of 28 V, and the amount of charge transferred was increased to 30 nC, which was a significant enhancement compared with that of natural wood. This study demonstrates that delignification can effectively optimize the charge regulation ability of wood and enhance the energy conversion efficiency of TENG. As shown in Fig. 24b, Shi et al. [271] used the deep eutectic solvent (DES) method for in-situ regeneration and chemical cross-linking modification of lignin by employing a DES system consisting of bile chloride and oxalic acid to dissolve the lignin in the wood, thereby breaking the hydrogen bonds between cellulose, and lemon as a natural cross-linking agent to form stable lignin-cellulose composite networks suitable for degradable friction nanogenerators. Luo et al. [266] treated the wood with NaOH/Na_2_SO_3_ solution for 7 h to partially remove lignin and hemicellulose and successfully optimized the microstructure of the wood, which was modified to exhibit 7.5-fold mechanical strength enhancement and a 71% increase in the surface charge density, which helped to enhance the friction charge storage capacity. These findings highlight the crucial role of structural modifications, such as partial delignification and polymer impregnation, in optimizing the performance of wood-based TENGs. As shown in Fig. 24c, Cheng et al. [272] further advanced this concept by developing a transparent wood-based triboelectric nanogenerator (TW-TENG), integrating delignification with UV-curable resin impregnation to achieve a synergistic enhancement in transparency, triboelectric output, and aesthetic appeal, resulting in a 6.5-fold voltage increase and 88.8% light transmittance.

**Fig. 25:**
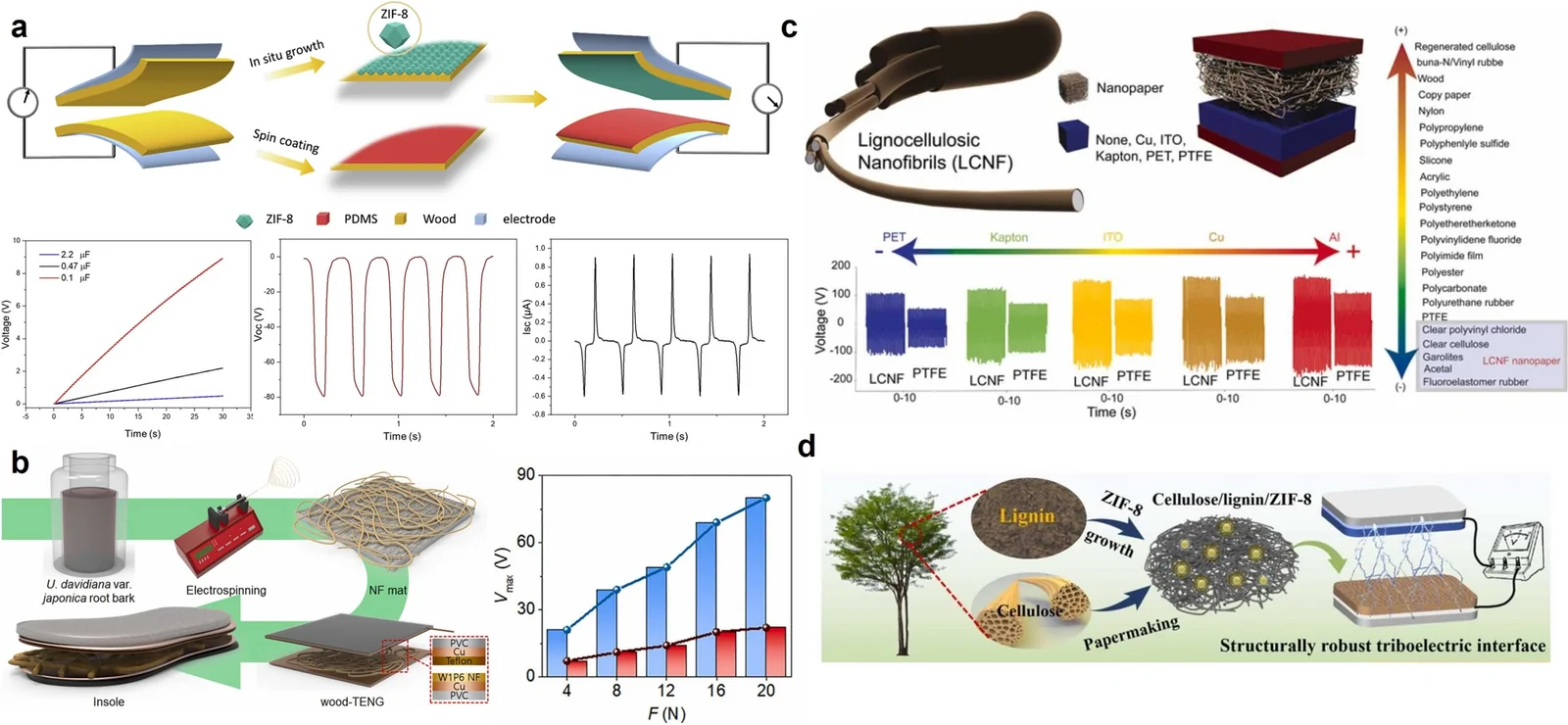
Wood-based composites and wood-derived materials for enhanced triboelectric nanogenerator (TENG) performance. **a** Single-electrode FW-TENG integrating in-situ grown ZIF-8 for tribo-positive polarity and PDMS coating for tribo-negative polarity, forming a high-polarity contrast interface to boost voltage and current output. Reproduced from Ref. [273]with permission from Elsevier, copyright 2021. **b** Electrospun wood-derived nanofiber mats (NFs) modified with wood-derived epoxy (WDE)/PCL composite to increase surface area, hydrophilicity, and energy conversion efficiency. Reproduced from Ref. [274] with permission from Elsevier, copyright 2022. **c** Lignin-retained cellulose nanofibers (LCNF) with hydrophobic lignin-rich surfaces acting as efficient tribo-negative materials, markedly enhancing TENG voltage and current output over PTFE. Reproduced from Ref. [262] with permission from Elsevier, copyright 2022. **d** Abrasion-resistant cellulose-based composite (CLZ) with ZIF-8 nanoparticles deposited via layer-by-layer assembly for improved durability and high power density in self-powered sensing and human–machine interfaces. Reproduced from Ref. [256] with permission from Elsevier, copyright 2025

In addition to improving the porosity of wood, the overall performance and application potential of TENG can be enhanced by compositing with functional materials to form wood-based composites or wood-derived materials, thereby optimizing charge transfer efficiency, mechanical durability and energy conversion properties. As shown in Fig. 25a, Sun et al.[273] constructed a single-electrode mode TENG (FW-TENG) by growing ZIF-8 in situ to impart friction-positive polarity to wood, spin-coating PDMS to enhance its friction-negative polarity, and forming a friction interface with high polarity contrasts, FW-TENG can charge a 0.1 µF capacitor up to 8.9 V in 30 s, and outputs 79.6 V and 0.94 mA at a scale of 10 cm × 8 cm, which is significantly better than that of unfunctionalized wood and demonstrates great potential for large-scale applications. As shown in Fig. 25b, the wood-derived nanofiber mats (NFs) prepared by Park et al. [274] electrospinning were optimized for friction initiation characteristics by enhancing the specific surface area and hydrophilicity through the composite modification of WDE and PCL, so that the output voltage of wood-TENG was significantly higher than that of PCL-TENG, up to 80 V, which demonstrated the excellent energy conversion performance. Additionally, beyond direct wood modification, utilizing wood-derived nanofibers as functional components further expands the potential of TENGs. By retaining lignin and optimizing the nanostructure, these materials enhance surface properties, charge transfer efficiency, and overall device performance. As shown in Fig. 25c, Tanguy et al.[262] prepared lignin-retained cellulose nanofibers (LCNF) by alkaline treatment of cedar bark with NaOH to remove the extracts, which were hydrolyzed and swollen, and then mechanically dissociated by milling at 1500 rpm for 20 cycles. LCNF exhibited enhanced hydrophobicity due to the lignin-rich surface, reached a contact angle of 67°, and acted as an efficient friction-negative material in TENG. Compared to conventional PTFE materials, LCNF-TENG increases output voltage by 160% to 360 V and short-circuit current by 120% to 28 µA. As shown in Fig. 25d, Li et al.[256] proposed an abrasion-resistant enhanced cellulose-based friction electronic material (CLZ composite) for high-performance self-powered sensors and human–machine interfaces. ZIF-8 nanoparticles were grown in a homogeneous system by methanol-extracted lignin (MeOH-lignin) as a soft template and deposited on a cellulose network by a layer-by-layer assembly (LBA) method, and the optimized CL_7_Z_8_ TENG showed a wear rate reduction of 64.96% after 5,000 friction cycles, the optimized CL7Z8 TENG achieved a 64.96% reduction in wear rate after 5,000 friction cycles, and the TENG achieved a maximum instantaneous power density of 346.41 mW m^−2^, which is 21 times higher than that of the pure cellulose TENG.

**Fig. 26:**
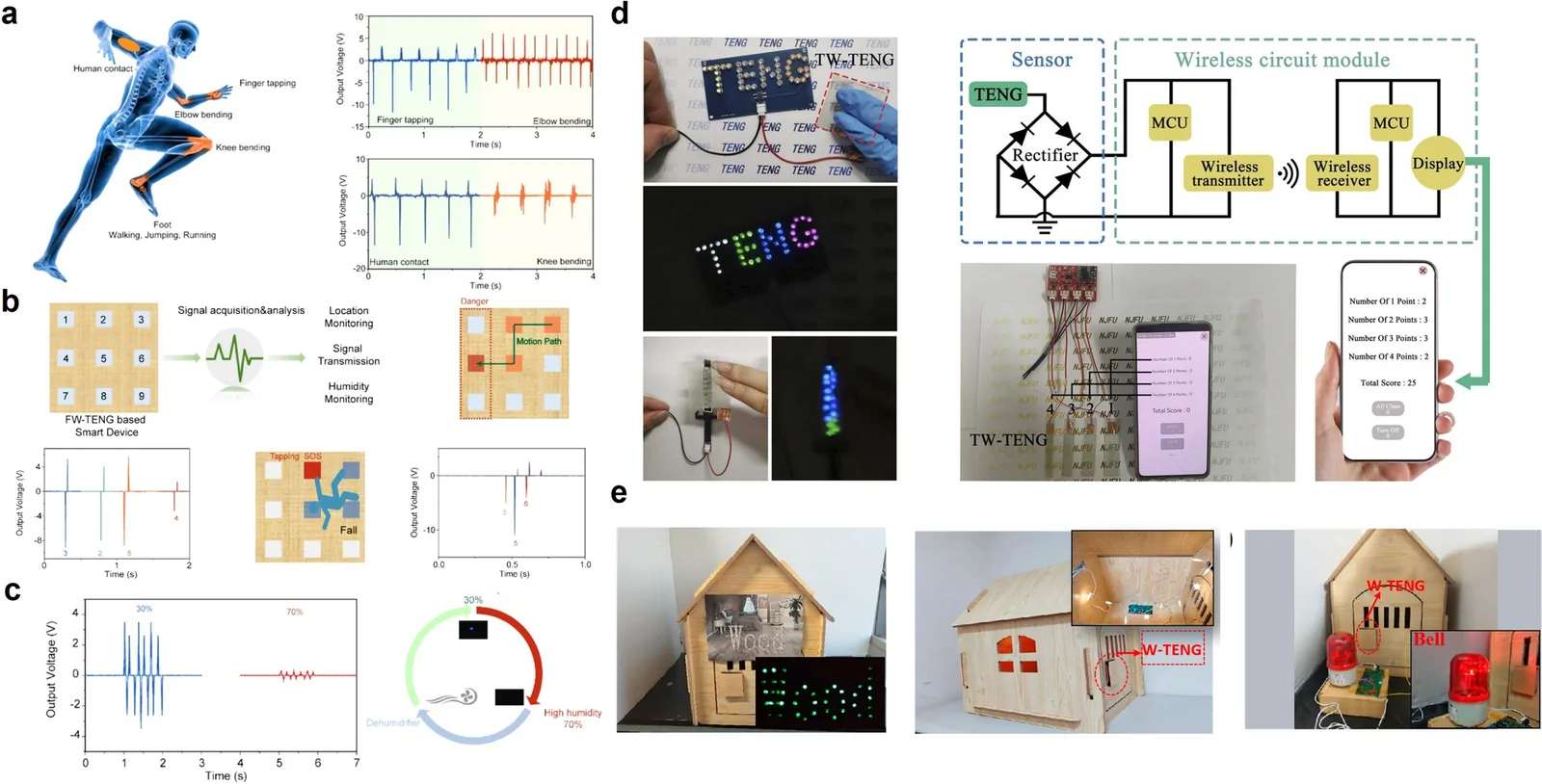
Applications of wood-based triboelectric nanogenerators (W-TENG) in smart home systems. **a** FW-TENG harvesting mechanical energy from human activities (walking, running, bending, etc.) for energy storage and short-term power supply. Reproduced from Ref. [257] with permission from Springer Nature, copyright 2024. **b** Intelligent floor sensor array based on FW-TENG for real-time walking trajectory monitoring and fall detection. Reproduced from Ref. [257] with permission from Springer Nature, copyright 2024. **c** Humidity sensing capability of FW-TENG for adaptive environmental control. Reproduced from Ref. [257] with permission from Springer Nature, copyright 2024. **d** TW-TENG for smart lighting, luminous signage, and wireless target shooting systems with real-time monitoring and scoring. Reproduced from Ref. [272] with permission from Elsevier, copyright 2024. **e** W-TENG for self-powered sensing applications in smart home and floor monitoring. Reproduced from Ref. [265] with permission from Elsevier, copyright 2020

Smart home has attracted much attention due to its significant advantages in enhancing the quality of human life. As the core components of smart home systems, electronic devices need to be environmentally friendly, with excellent stability and efficient energy conversion performance. In this context, friction nanogenerators (TENG) are one of the most promising solutions to this problem due to their ability to efficiently convert low-frequency mechanical energy into electrical energy. As shown in Fig. 26a, the all-wood friction nanogenerator (FW-TENG) developed by Ma et al. [257] can efficiently collect the mechanical energy generated by human body movements (e.g., walking, jumping, knocking, bending, etc.) and convert it into electrical energy. For example, FW-TENG can generate an output voltage of 210 V when walking and 300 V when running, and it can also be used for energy storage, as experimental data show that a 1 μF capacitor can be charged to 3 V in 10 s, and a 47 μF capacitor can be charged to 0.2 V, which can be used to provide short-term power supply for small electronic devices. As shown in Fig. 26b, In terms of intelligent sensing, an intelligent floor sensor array based on FW-TENG is constructed, which can monitor the user's walking trajectory in real time and automatically trigger an alarm when an abnormal fall is detected. In addition, FW-TENG can also be used for environmental monitoring. As shown in Fig. 26c, Experiments show that when the humidity increases from 30% to 80%, the TENG output voltage decreases by 82%, and this feature can be used in the automatic humidity adjustment system of the smart home to realize adaptive dehumidification control. As shown in Fig. 26d, Cheng et al. [272] utilized the high transparency of TW-TENG to enable the LED light source to penetrate through its structure for smart lighting or luminous signage applications such as emergency escape routes. In addition, TW-TENG can also charge energy storage devices and drive LED bulbs. Experimental data showed that the 0.47 μF capacitor was charged to 1.83 V in 50 s, while the 10 and 100 μF capacitors were charged to 0.53 and 0.18 V, respectively, which further validated its energy storage and power supply capabilities. Meanwhile, the study also demonstrated the application of TW-TENG in a smart target shooting system, which can realize remote real-time monitoring and smart scoring by transmitting wireless signals to cell phones. As shown in Fig. 26e, Hao et al. [265] Natural wood-based friction nanogenerator (W-TENG) for self-powered sensing in smart homes and floors.

Wood-based triboelectric nanogenerators (W-TENG) integrate the intrinsic biodegradability, renewability, and hierarchical porous structure of wood with advanced triboelectric and structural engineering strategies to achieve sustainable, high-efficiency mechanical-to-electrical energy conversion for self-powered systems [273, 275]. Through surface modification (e.g., oxygen plasma, chemical functionalization, laser-induced graphene), structural optimization (e.g., delignification, carbonization, polymer impregnation), and functional material integration (e.g., ZIF-8, PDMS, wood-derived nanofibers, lignin-retained cellulose nanofibers), W-TENG exhibit significant enhancements in voltage output (up to 360 V), current (up to 0.94 mA), and power density (up to 0.76 W m^−2^), with greatly improved durability (e.g., ~ 65% wear reduction after 5000 cycles). These advancements enable diverse smart home applications—including energy harvesting from human motion, intelligent floor sensing for gait and fall detection, humidity monitoring for adaptive climate control, and transparent TENG-based smart lighting and signage—while maintaining environmental compatibility [276]. Collectively, W-TENG research demonstrates that precise microstructural tailoring, hybrid material assembly, and application-driven design can transform wood into a high-performance, multifunctional, and eco-friendly energy platform for next-generation IoT and smart living systems [277].

## Summary and Prospects

In this review, we systematically summarize the structural characteristics, performance advantages, and multidimensional functionalization pathways of wood as a natural and renewable material. As a fundamental step in functionalization, mechanical processing—including pulverization, rotary cutting, precision sawing, and compression—can increase the specific surface area, expose more active sites, and preserve the natural porous structure and cellulose orientation, thereby optimizing mechanical and conductive properties while providing an ideal substrate for subsequent treatments. On this basis, carbonization transforms wood into carbon-based materials with high electrical conductivity and hierarchical porosity, which, when combined with the deposition of metal oxides (e.g., MnO_2_, Co(OH)_2_), can achieve high specific capacitance (up to 3,395 mF cm^−2^) and excellent cycling stability (88.6% capacitance retention). Laser-induced graphene (LIG) technology enables the direct fabrication of highly conductive micro/nanopatterns on wood surfaces, offering a low-cost, biodegradable platform for flexible electronics and sensors. Delignification selectively removes lignin, significantly enhancing optical transmittance, hydrophilicity, and interfacial bonding capacity. Nanocomposite strategies—based on in situ growth, surface compositing, and structural regulation—integrate metals, metal oxides, carbon-based, and polymeric nanomaterials into the multiscale architecture of wood, markedly improving its electrochemical, optical, and mechanical performance. Benefiting from these functionalization strategies, wood-based materials have demonstrated outstanding performance and sustainability potential in diverse applications, including energy storage devices (lithium/sodium-ion batteries, metal–air batteries), water treatment (photocatalytic degradation, adsorption–filtration, solar-driven evaporation), solar energy harvesting (photovoltaic and photothermal conversion), hydrovoltaic power generation (maximum power density of 6.75 μW cm^−2^), and triboelectric nanogenerators (output voltage up to 360 V, power density of 0.76 W m^−2^). These advances have accelerated the transformation of wood from a traditional structural material into a high-performance, multifunctional, and cross-disciplinary green technology platform.

Despite these advancements, several fundamental and practical challenges remain. Although functionalized wood integrates structural versatility, renewable origin, and multifunctional capabilities, its practical deployment is still constrained by a series of intrinsic and process-related limitations. These include the need for precise control over wood’s microstructure and chemical uniformity during processing, as natural variability in species and growth conditions often leads to performance inconsistency. Furthermore, the long-term mechanical robustness and functional stability of functionalized wood under fluctuating environmental conditions—such as humidity, temperature, and mechanical stress—require systematic evaluation. Scalability also presents a bottleneck, as many current fabrication methods are energy-intensive or involve hazardous chemicals. Therefore, it is critical to develop green, scalable, and reproducible processing routes that align with sustainable manufacturing principles and lifecycle safety. Additionally, wood’s intrinsic anisotropy complicates its integration into conventional device architectures, requiring innovative strategies in interfacial design and material coupling to ensure consistent performance. Addressing these bottlenecks will be key to translating the conceptual advantages into deployable, high-impact solutions. Nevertheless, the convergence of material sustainability, structural versatility, and multifunctional capability positions functionalized wood as a transformative candidate in the future landscape of green technologies. As science and technology continue to advance, functionalized wood is expected to assume a greater role in shaping a sustainable and intelligent world. Beyond its conventional structural use, wood will serve as an active material foundation—integrating ecological wisdom with technological innovation—to help build a greener, more resilient, and harmonized planet.

Building on this potential, functionalized wood is finding opportunities across multiple frontiers where its unique structural features and tunable properties can be harnessed for advanced applications. In biomedical engineering, wood-derived scaffolds with aligned channels and controllable porosity may serve as promising candidates for tissue engineering, drug delivery, and biosensing, by mimicking vascular architectures and supporting biocompatibility. In the domain of electronics, functionalized wood can be engineered into dielectric substrates, ionic conductors, and carbonized components, enabling the development of flexible, biodegradable devices such as transient sensors and bioresorbable circuits. Additionally, the anisotropic thermal and electrical properties of wood offer opportunities for passive cooling and directed signal transport. In environmental science, the material’s high porosity and surface modifiability make it suitable for applications including solar-driven water purification, pollutant removal, and carbon capture, particularly when integrated with photocatalytic or bioactive functionalities. While much work remains to fully realize these applications, we are optimistic that continued advances in nanoengineering and functional integration will further enhance the role of functionalized wood as a cross-disciplinary platform to address pressing global challenges.
